# Risk Factors for Acute Rheumatic Fever: Literature Review and Protocol for a Case-Control Study in New Zealand

**DOI:** 10.3390/ijerph16224515

**Published:** 2019-11-15

**Authors:** Michael G Baker, Jason Gurney, Jane Oliver, Nicole J Moreland, Deborah A Williamson, Nevil Pierse, Nigel Wilson, Tony R Merriman, Teuila Percival, Colleen Murray, Catherine Jackson, Richard Edwards, Lyndie Foster Page, Florina Chan Mow, Angela Chong, Barry Gribben, Diana Lennon

**Affiliations:** 1 Department of Public Health, University of Otago, Wellington 6021, New Zealand; jason.gurney@otago.ac.nz (J.G.); jane.oliver@unimelb.edu.au (J.O.); nevil.pierse@otago.ac.nz (N.P.); richard.edwards@otago.ac.nz (R.E.); 2 School of Medical Sciences, University of Auckland, Auckland 1010, New Zealand; n.moreland@auckland.ac.nz; 3 Microbiological Diagnostic Unit Public Health Laboratory, Department of Microbiology & Immunology, University of Melbourne at The Doherty Institute for Infection and Immunity, Melbourne 3010, Australia; deborah.williamson@unimelb.edu.au; 4 Green Lane Paediatric and Congenital Cardiac Services, Starship Children’s Hospital, Auckland District Health Board, Auckland 1023; New Zealand; nigelw@adhb.govt.nz; 5 Department of Paediatrics, University of Auckland, Auckland 1142, New Zealand; d.lennon@auckland.ac.nz; 6 Biochemistry Department, University of Otago, Dunedin 9054, New Zealand; tony.merriman@otago.ac.nz; 7 School of Population Health, University of Auckland, Auckland 1142, New Zealand; t.percival@auckland.ac.nz; 8 KidzFirst Children’s Hospital, Auckland 1640, New Zealand; Florina.ChanMow@middlemore.co.nz; 9 Faculty of Dentistry, University of Otago, Dunedin 9054, New Zealandlyndie.fosterpage@otago.ac.nz (L.F.P.); 10 Auckland Regional Public Health Service, Auckland District Health Board, Auckland 0622, New Zealand; catherine.jackson@northlanddhb.org.nz; 11 CBG Health Research Ltd, Auckland 0651, New Zealand; Angela.chong@cbg.co.nz (A.C.); barry.gribben@cbg.co.nz (B.G.); 12 Starship Children’s Hospital, Auckland District Health Board, Auckland 1023, New Zealand

**Keywords:** acute rheumatic fever, rheumatic heart disease, case-control, risk factors, group A streptococcus, housing, crowding, environmental tobacco smoke, health care access, sore throat, skin infection

## Abstract

Acute rheumatic fever (ARF) and its sequela, rheumatic heart disease (RHD), have largely disappeared from high-income countries. However, in New Zealand (NZ), rates remain unacceptably high in indigenous Māori and Pacific populations. The goal of this study is to identify potentially modifiable risk factors for ARF to support effective disease prevention policies and programmes. A case-control design is used. Cases are those meeting the standard NZ case-definition for ARF, recruited within four weeks of hospitalisation for a first episode of ARF, aged less than 20 years, and residing in the North Island of NZ. This study aims to recruit at least 120 cases and 360 controls matched by age, ethnicity, gender, deprivation, district, and time period. For data collection, a comprehensive pre-tested questionnaire focussed on exposures during the four weeks prior to illness or interview will be used. Linked data include previous hospitalisations, dental records, and school characteristics. Specimen collection includes a throat swab (Group A Streptococcus), a nasal swab (Staphylococcus aureus), blood (vitamin D, ferritin, DNA for genetic testing, immune-profiling), and head hair (nicotine). A major strength of this study is its comprehensive focus covering organism, host and environmental factors. Having closely matched controls enables the examination of a wide range of specific environmental risk factors.

## 1. Introduction

In 2012, the New Zealand (NZ) Government announced that one of the top ten goals of the public sector was to reduce the incidence of first episode acute rheumatic fever (ARF) by two-thirds, to 1.4 per 100,000 by mid-2017 [1]. To achieve this goal, the Government expanded the Rheumatic Fever Prevention Programme (RFPP), which was established in 2011. However, there are important gaps in knowledge about the aetiology, pathogenesis and risk factors for ARF that currently limit the ability to develop and implement effective interventions [2].

The Health Research Council of New Zealand (HRC) subsequently released a Rheumatic Fever Research Partnership request for proposals “to purchase high quality research that will investigate Rheumatic Fever in NZ and support policy makers and practitioners to reduce the incidence and impact of this condition” [3]. In response, a cross-institution research group secured funding from the HRC to conduct a study to identify risk factors for ARF in NZ. The study sought to identify important organism, host and environmental factors—in particular, those that are modifiable, those contributing to the large ethnic inequalities in incidence, and those that can provide important insights into the causal pathways of ARF.

ARF is an auto-immune disease triggered in response to infection caused by Group A Streptococcus (GAS) [4,5,6]. This exposure is considered to be largely from pharyngitis, though skin infection may also have a role [7,8,9]. There is also suggestive evidence that non-Group A Streptococcal infections (Group C and G) may be able to trigger this auto-immune response [10,11]. The autoimmune mechanisms producing ARF involve the production of antibodies and T-cells that react with self-antigens. Antibodies have been observed to be deposited in the myocardium and valves of ARF patients following death [12]; interstitial immune nodules (known as Aschoff bodies) are a hallmark of rheumatic carditis [13]; CD4^+^ and CD8^+^ T cells have been shown to be attached to rheumatic heart valve endothelium [14].

Symptoms of ARF include fever, joint pain, and in some cases, skin manifestations (erythema marginatum, subcutaneous nodules), and chorea. Up to 80% of ARF cases have carditis found by echocardiogram at diagnosis. These changes may persist or develop into chronic rheumatic heart disease (RHD), whereby cardiac valves are permanently damaged [15]. Recurrent bouts of ARF greatly increase the chances of worsening RHD [5,16]. Following the initial episode of ARF, individuals are at high risk of recurrences [16,17]. Recurrences can be largely prevented by secondary prophylaxis with intramuscular injection of benzathine penicillin G (BPG) every 28 days [16,18].

ARF and RHD continue to cause a large global burden of morbidity and mortality, predominantly in low-income countries [19]. In high-income countries, improvements in healthcare and living conditions throughout the 20th century are thought to have led to ARF virtually disappearing [20]. Exceptions are indigenous and Pacific peoples in Australia and NZ where ARF has persisted, with marked ethnic and socioeconomic disparities in occurrence [21,22].

This paper presents the protocol for a study to identify risk factors for ARF in NZ, including a description of relevant aspects of ARF epidemiology in NZ, a literature review of the current knowledge of ARF risk and protective factors, and a description of the study’s aims, hypotheses, and methods.

## 2. Epidemiology and Impact of ARF in New Zealand

The epidemiology of ARF in NZ had been described in several sources up to 2014 when this study commenced [21,23,24,25,26]. ARF epidemiology has also been described for some specific regions of NZ [27,28,29]. These analyses showed several key features of the epidemiology that affect the design of research studies investigating this disease.

We performed an additional descriptive analysis to inform the development of the protocol for this study. For the purpose of this brief analysis, we largely used hospitalisation data for the 5-year period 2010 to 2014 leading up to the start of this study, although some analyses covered the 20-year period of 1995 to 2014. These data were filtered and analysed using a standard method adopted by the NZ Ministry of Health for estimating the incidence of initial ARF hospitalisations (using principal diagnosis of ARF and excluding any cases previously assigned a principal or additional diagnosis of ARF or RHD) [25]. This method inevitably has some error in case classification as hospitalisations tend to over-count the number of diagnosed cases by around 25–33% [27,28]. Additionally, at least 10% of true cases may go undetected [30].

### 2.1. Incidence of ARF

**Figure 1:**
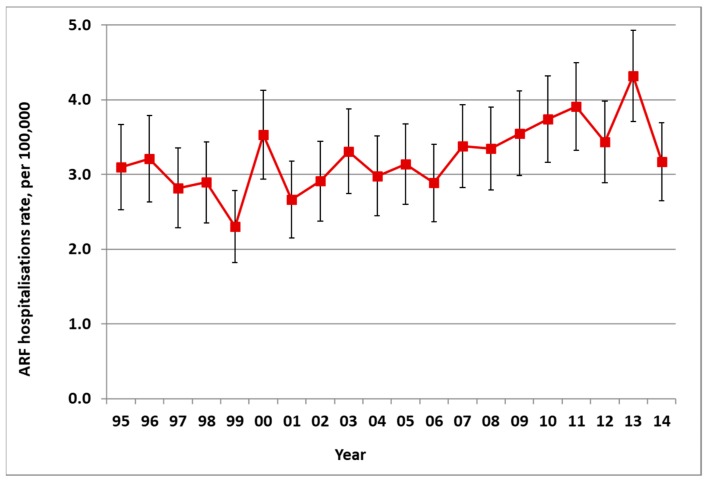
Acute rheumatic fever (ARF) incidence by year, initial hospitalisation rate per 100,000 with 95% CIs, 1995 to 2014.

There was an average of 172 new cases of ARF hospitalised in NZ each year from 2010 to 2014, ranging from 149 to 200 (average rate of 3.9 per 100,000). This was a significant increase in incidence from the 1990s (with an average of 111 cases a year in 1995–1999, a rate of 3.0 per 100,000) (Figure 1). In addition to these first admissions, there were also recurrences of ARF that caused additional hospitalisations.

**Figure 2:**
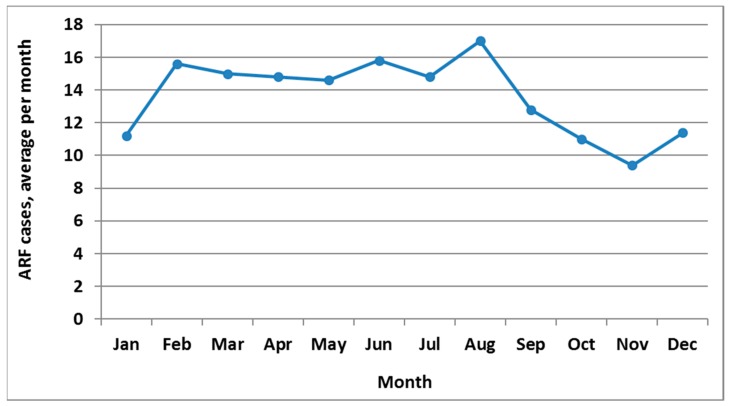
ARF incidence by month, initial hospitalisation numbers, average for 2010 to 2014.

ARF is a moderately seasonal disease with peak incidence in August (Figure 2). The highest incidence season is winter (June to August, with 29.0% of cases), closely followed by autumn (27.0%), then summer (23.6%) and spring (20.4%).

### 2.2. Distribution of ARF

#### 2.2.1. Geographic Distribution

**Figure 3:**
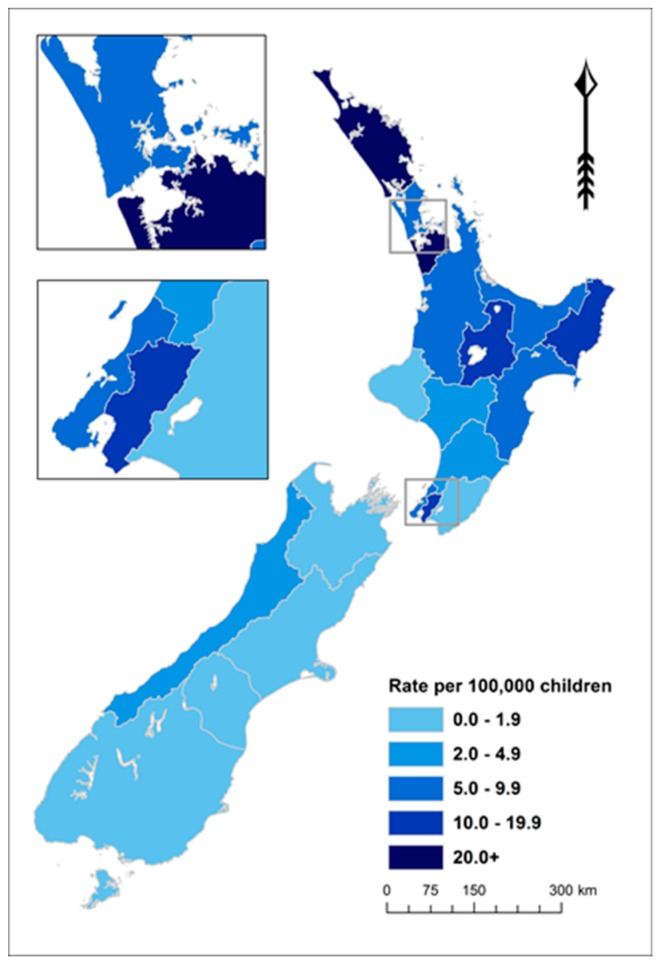
ARF incidence by District Health Board (DHB) for children aged <20 years, average annual initial hospitalisation rate per 100,000, 2010–2014.

ARF is concentrated in the North Island of NZ (97.7% of cases in those aged <20 years over this period). The 11 District Health Boards (DHBs) with rates of 5.0 per 100,000 or higher accounted for 94.8% of these cases over this 5-year period (Figure 3 and Supplementary Table S1).

#### 2.2.2. Age and Sex Distribution

**Figure 4:**
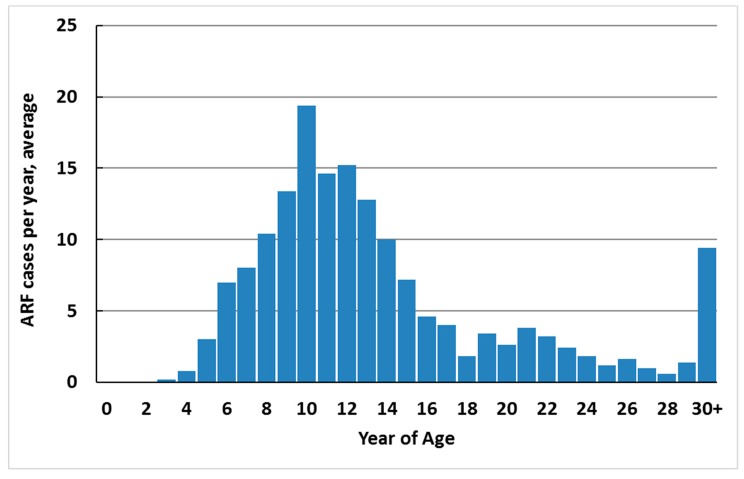
ARF incidence by single year of age, average annual initial hospitalisation number, 2010–2014.

ARF cases occur predominantly in school-aged children (79.1% of total new cases in 2010–2014 were aged 5–17 years) (Figure 4). Over this period, 82.6% of cases were less than 20 years of age (12.2 per 100,000). The median age of onset was 12 years. Cases were unusual in children less than 4 years of age (one case was 3 years old during 2010–2014, and no cases were younger). The upper age limit is not defined, but first hospitalised cases 20 years of age and over are likely to include a high proportion of recurrences who had ARF earlier in life and were asymptomatic, did not seek care, or were misdiagnosed or miscoded in hospital data.

A small but significant majority of cases were male (56.7% during the 2010–2014 period).

#### 2.2.3. Ethnic Distribution

**Figure 5:**
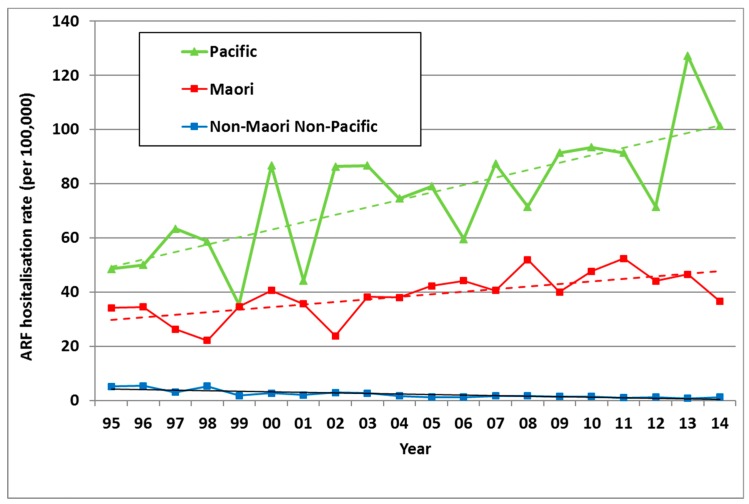
ARF incidence by prioritised ethnicity and year, initial hospitalisation rate per 100,000, 5–14 year olds, 1995 to 2014.

In NZ, ARF risk has become highly concentrated in Māori and Pacific children. Of all ARF cases aged <20 years in the 2010–2014 period, 51.7% were Māori and 43.4% were Pacific, with only 4.9% belonging to European and other ethnic groups. Even after adjusting for age and deprivation, rate ratios (RR) remain high for Māori (14·5) and Pacific children (20·6) compared with European/other children (based on the 2010–2013 period) [26]. This elevated ARF risk for Māori and Pacific children is far higher than that seen for other infectious diseases [31]. These ethnic inequalities increased from 1995 to 2014 (Figure 5). Note that this analysis is based on prioritised ethnicity where respondents indicating membership of more than one ethnic group are allocated to a single ethnic group based on a prioritised order of Māori, Pacific, Asian and European/other. Consequently, the European/other ethnic group effectively refers to non-Māori, non-Pacific, and non-Asian people, in other words, primarily Europeans [32].

#### 2.2.4. Distribution by Deprivation

**Figure 6:**
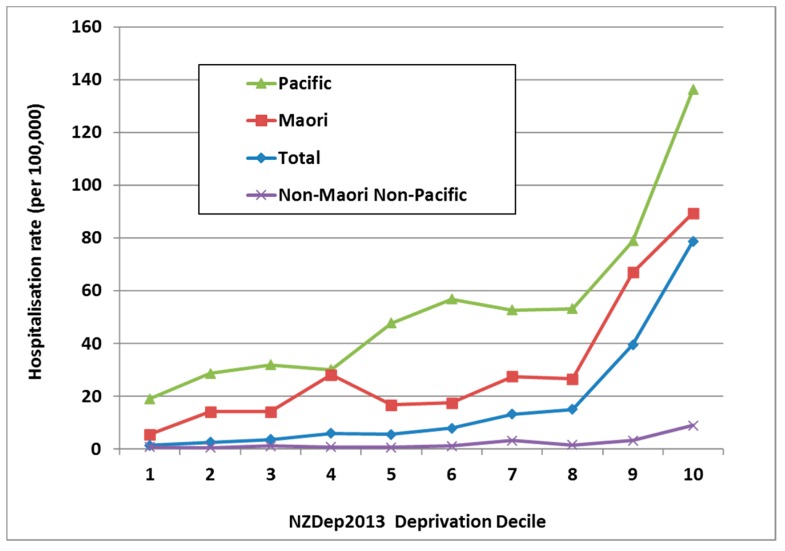
ARF incidence by prioritised ethnicity and deprivation level, initial hospitalisation rate per 100,000, 5–14 year olds, average for 2010–2014.

In NZ, it is common to use an area-based measure of social deprivation, known as NZDep [33]. It uses a decile scale, with NZDep10 neighbourhoods containing the most deprived 10% of the population [34]. ARF is strongly associated with living in a more deprived neighbourhood with 71.2% of cases aged 5–14 years living in the most deprived quintile (i.e., NZDep 9 and NZDep 10). This effect is independent of ethnicity (Figure 6).

### 2.3. Impact on RHD, RHD Mortality and Healthcare Costs

**Figure 7:**
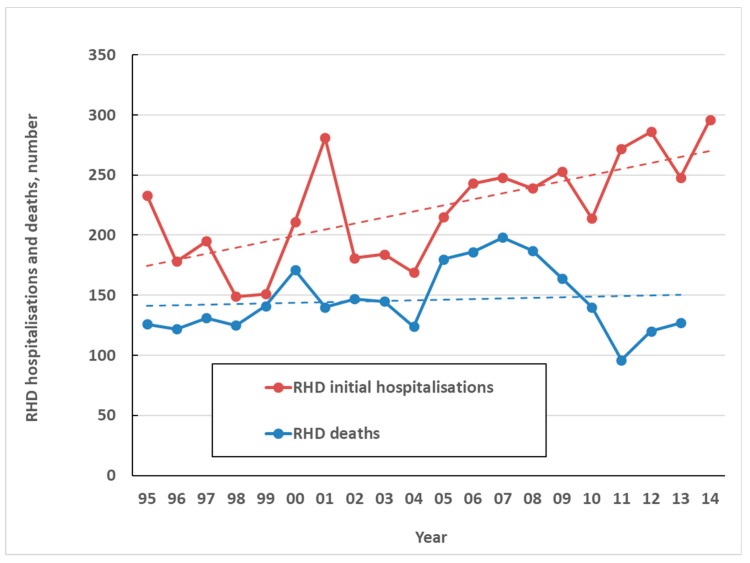
Coded RHD initial hospitalisations and deaths by year, 1995 to 2014.

The high health impact of ARF comes primarily from is sequela, RHD. In NZ only one death was attributed to ARF directly over the five-year period from 2010 to 2014. By contrast, over the same period, there were an average of 263 initial hospitalisations coded for RHD per year (6.0 per 100,000) and an average of 125 coded RHD deaths a year (2.8 per 100,000) (Figure 7). This high burden of chronic disease and death resulting from ARF, along with large ethnic inequalities, make it one of New Zealand’s most important infectious diseases for prevention and control. A limitation of this analysis is that the validity of RHD coding is uncertain, particularly in older age groups, and requires further research.

The direct annual cost to NZ of ARF and RHD is estimated to be about $12 million for hospitalizations (71% as heart valve surgery) [35].

## 3. Existing Knowledge about Risk and Protective Factors for ARF and RHD

### 3.1. Causal Pathway for GAS Exposure, ARF and RHD

**Figure 8:**
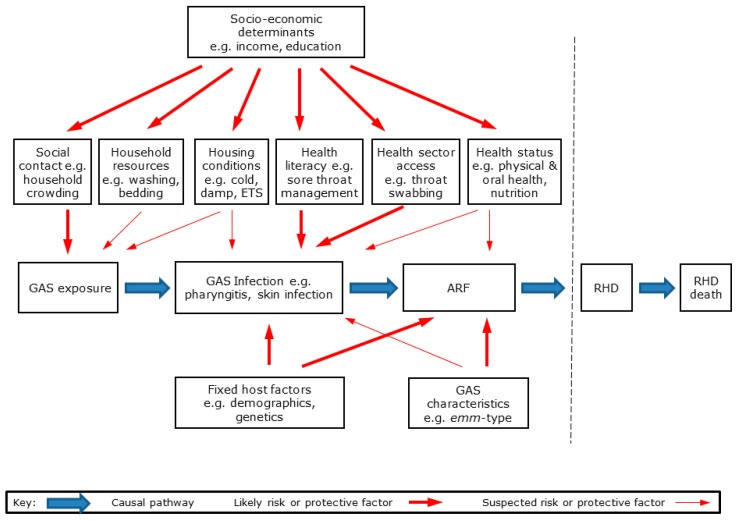
Causal pathway from GAS exposure to ARF and RHD showing major hypothesised groups of risk and protective factors.

RHD is a multi-stage disease initiated by GAS infection, leading to ARF and then RHD [5]. The likely causal pathways from GAS exposure to RHD are shown in Figure 8.

As with most infectious diseases, a range of environmental, host, and organism factors interact to influence disease risk and outcomes [36]. These hypothesised risk and protective factors are shown in Figure 8. For the purpose of this study, we grouped these factors into seven broad categories:

- **Preceding GAS infections** of throat and skin, which may initiate ARF;
- **Environmental risk factors**, notably the number of social contacts, household crowding and bed sharing, household resources, including those for washing, teeth cleaning, and laundry, housing conditions, including damp, and cold, environmental tobacco smoke (ETS) exposure, exposure to biting insects and skin injuries;
- **Healthcare factors**, notably health literacy and healthcare access;
- **Health and nutrition factors**, including health status, oral health status and services, nutrition;
- **Social determinants**, including income, education and housing tenure;
- **Predisposing host factors**, including demographic, inherited and early development;
- **Organism factors**, including molecular typing data.

The current knowledge about the potential role of these factors has informed our decisions about hypotheses to test in this study and is summarised in the following sections.

### 3.2. Knowledge of Risk and Protective Factors for ARF from Previous Epidemiological Studies

There are considerable gaps in knowledge about the aetiology, pathogenesis and risk factors for ARF that currently limit our ability to develop and implement effective interventions for this disease [5,17,37,38]. Key organism, host and environmental risk factors that have been associated with the development of this complex disease are discussed in greater detail in the next sections.

A literature review was carried out to identify studies reporting factors associated with an increased risk of ARF and RHD (Supplementary Table S2). We focused on published epidemiological studies of risk factors for ARF and RHD, based on individual-level data (i.e., cohort, case-control and cross-sectional studies where ARF or RHD was the primary outcome).

These studies include 24 reports that had ARF incidence as an outcome [39,40,41,42,43,44,45,46,47,48,49,50,51,52,53,54,55,56,57,58,59,60,61,62]. They were based on 16 different study populations because eight reports used reanalysis of the same or overlapping datasets (in Yugoslavia [42,43,45,46], Bangladesh [48,49,50], Turkey [51,52,53] and NZ [61,62]). A further 22 reports had RHD incidence, prevalence or death as the outcome [57,60,63,64,65,66,67,68,69,70,71,72,73,74,75,76,77,78,79,80,81,82]. Two of these reports included both ARF and RHD outcomes [57,60]. Two reports included RHD outcomes but were focussed on ARF so they are not included in the RHD summary [61,62].

The ARF studies include 20 case-control studies [39,40,42,43,44,45,46,48,49,50,51,52,53,54,55,56,57,58,59,60], two cross-sectional studies [41,47] and two cohort studies [61,62]. Most of the studies, particularly the more recent ones, were conducted in low- and middle-income countries with only a minority in high-income countries. All were relatively small, and most were of poor quality, with only a minority reporting results of multivariate analyses [45,46,48,49,50,60,61,62].

The 20 RHD studies were predominantly cross-sectional and based on echocardiographic screening programmes [60,63,65,67,68,69,71,73,74]. There were also 10 case-control studies [57,64,70,72,75,76,78,80,81,82], two of which were nested within screening programmes. Two studies were retrospective cohorts [66,77] and one was a prospective cohort [79]. Most of the studies were conducted in low- and middle-income countries where RHD remains prevalent. Again, most were of relatively poor quality, with only a minority reporting results of multivariate analyses [67,71,73,76,79]. We also summarised literature on GAS infections (throat and skin) in the text.

Most of the studies examined a set of socio-economic and environmental risk factors, notably housing conditions. A few included nutritional factors. Some focussed only on genetic markers. Two specifically investigated associations with psychiatric disorders. Findings are discussed further under specific groups of risk factors in the sections that follow. In addition, the published literature includes a number of ecological studies that have explored the relationship between ARF rates and neighbourhood characteristics. These studies included research conducted in the United States [83] and NZ [84], both of which found that high ARF rates were strongly associated with living in poor neighbourhoods and crowded households. Ecological studies have inherent weaknesses and are given less weight in this review.

### 3.3. Preceding GAS Infection of Throat and Skin as the Initiator of ARF

A preceding GAS infection is assumed to be a necessary condition for development of ARF. Half to two thirds of ARF cases report a preceding sore throat presumed to be caused by GAS pharyngitis [10,85]. As the latent period following GAS pharyngitis before the onset of ARF symptoms is typically about 21 days [86], the absence of a positive culture at the time of ARF symptom onset is not uncommon. In a NZ randomised trial of improved access to sore throat management using school clinics, episodes of sore throats preceded development of ARF (with appropriate raised streptococcal serology) in 14 of 19 (74%) cases presenting with ARF [10]. A Yugoslavian case-control study identified frequently experiencing a sore throat as a risk factor for ARF [45].

There is evidence that GAS skin infections (impetigo) can also initiate autoimmune processes, leading to ARF [8,9]. In Australian aboriginal populations, streptococcal skin infections are far more commonly associated with ARF than streptococcal pharyngitis, supporting the view that skin infections may be an initiator of ARF in this population [8,87,88]. In NZ, streptococci temporally associated with and obtained from endemic ARF cases during 1984–1992 had M-types more typical of skin, rather than throat, infections circulating in the community [7]. More recently, *emm*-typing of GAS strains obtained from ARF cases in NZ (2006–2014) showed a strong association with strains usually identified in pyoderma cases, again suggesting a possible role for skin infection in ARF [89]. The incidence of hospitalised skin infections in NZ is higher in Māori and Pacific children, although the magnitude of this inequality is far less than that seen for ARF [90]. However, skin infections in children in one South Auckland community at high risk of ARF appeared considerably less common than GAS positive throat infections [91,92]. If GAS skin infections are important for initiating ARF, then other causes of skin trauma, such as eczema and insect bites, may also be plausible risk factors [93].

Scabies infections may be important as a site of GAS co-infection. This infection is common in aboriginal and Pacific Island populations who experience the highest rates of ARF [94]. There is growing evidence concerning the molecular mechanisms that may allow scabies infestations to facilitate GAS infection of skin lesions [95]. A recent NZ data linkage study reported a strong association between ARF and scabies infection [62].

### 3.4. Environmental Risk Factors for ARF

#### 3.4.1. Number of Social Contacts

GAS pharyngitis is a highly infectious disease spread via salivary and nasal droplet transmission [96]. Almost half of the siblings of cases with GAS pharyngitis become infected [97]. Being in close proximity to others is a known risk factor for GAS transmission, with outbreaks well documented in schools, daycare centers, military barracks, and crowded homes [98]. GAS transmission occurs rapidly in cramped living conditions, which was considered to be a key factor mediating ARF outbreaks in US military camps [99,100,101].

Humans are an established reservoir for GAS, so having a large social network is a plausible risk factor for ARF as it increases the effective reproduction number for infection. ARF incidence peaks in school-aged children, so it is conceivable that the patterns of high social contact in that age group influences disease risk. Environmental risk factors, which influence the survival and transmission of GAS organisms, are also likely to be important. It is plausible that a cough caused by another respiratory infection could facilitate GAS transmission by generating an aerosol containing GAS organisms, so might be a risk factor if occurring in close contacts.

#### 3.4.2. Household Crowding, Including Bed Sharing

Household crowding is also a highly plausible risk factor for ARF as it increases the effective reproduction number for infections in the home. Household crowding can manifest in a range of ways, including high household occupancy, a deficit of bedrooms or space for the number of residents, and bed sharing. Household crowding has been identified as a risk factor for other bacterial diseases in NZ, notably meningococcal disease [102], pneumonia in children [103] and tuberculosis [104].

A small number of cohort and cross-sectional studies have investigated the relationship between household crowding and the risk of GAS throat and skin infection. A cohort study in South Africa found no association between household crowding (persons per bedroom) and throat swab culture for GAS [105]. Another study in India found no association between the area per person and the number of episodes of GAS positive sore throats per year [106]. By contrast, a cohort study in Singapore found a positive association between household crowding (persons per room) and GAS incidence [107]. A cross-sectional study in Bangladesh found that GAS prevalence was higher in children from large families [108], whereas a cross-sectional study of school children aged 6–11 years in Thailand found no association between GAS detection in throat swabs and household size or bedroom sharing [109]. A cross-sectional study of a Jewish population in a London borough found a significant positive association between crowding (children per bedroom) and pharyngeal GAS carriage (OR 1.95, 95% CI 1.25–3.01). This was one of the few well designed studies that also used multivariate analyses [110].

Some households in remote Australian communities at risk of ARF have been intensively studied to measure the acquisition of pharyngitis and pyoderma [87,111]. One of these cohort studies found a correlation between the number of cases of pyoderma per household and the number of people per bedroom [87]. Another found a correlation between *emm* subtype acquisition and household size in some communities [111].

Household crowding has been one of the factors most consistently examined by risk factor studies of ARF and RHD. Several ARF studies have reported an association between disease risk and measures of household crowding, although based on relatively small size and univariate results, in Australia [40], Hawaii [54], and Bangladesh [60]. A higher quality case-control study in Yugoslavia in the 1980s found significant associations with reduced living space (<5 m^2^ per person and ≥2 people per room), but these associations were no longer significant in the multivariate analysis [42,45]. Similarly, a case-control study in Bangladesh in the 1990s reported positive associations with small dwelling size and large families, but these associations were not significant in the multivariate analysis [48].

There have been several cross-sectional studies of RHD in low- and middle-income countries that have reported on univariate associations of RHD with measures of household crowding, including in South Africa [63], Kenya [65], Ethiopia [68], Pakistan [69], Yemen [74], and Fiji [75]. Findings from these studies were inconsistent, with some evidence of an increased risk associated with crowding only reported in the studies from South Africa [63] and Yemen [74].

Four higher quality studies have reported multivariate associations between RHD, based on echocardiographic screening, and measures of household crowding. A cross-sectional study in Congo found a significant association between larger household size (>8 people) and RHD [67]. By contrast, a cross-sectional study in India did not find an association with household crowding on multivariate analysis [73]. A case-control study in Uganda identified an association with reduced space per person (<90 square feet) [76]. A prospective cohort study in New Caledonia found that RHD persistence was associated with having ≥3 people per bedroom [79].

There have been two reported retrospective analyses of risk factors for RHD in high-income countries. One cohort study in the UK found no association between measured household crowding as a child and death from RHD in later life [66]. Another cohort study in Finland found that growing up in large households was associated with an increased risk of occurrence and death from RHD, based on univariate results [77].

We identified one study that reported on the association of ARF with bed sharing. This Yugoslavian case-control study found an association with bed sharing (≥2 people per bed) which disappeared in the multivariate analysis [42,43,45].

In NZ, an ecological study found that the risk of ARF was associated with neighbourhood deprivation, household crowding, and the proportion of 5–14 year olds in the area [84]. The pilot for the NZ risk factors study in 2012–2013 found that household crowding was common, with 58% of participants experiencing a bedroom deficit of one or more, including 35% with a bedroom deficit of two or more (severe crowding) [112]. This level was markedly higher than that reported for Māori and Pacific children (in the 2013 census, 23% of Māori children experienced a bedroom deficit of at least one, and 42% of Pacific children). In addition, the pilot study found that 49% of ARF cases shared their bed with one or more other people.

#### 3.4.3. Household Resources, including those for Washing and Laundry

A lack of washing facilities and resources may contribute to an increase in bacterial load on the skin of household members or on inanimate objects, resulting in increased transmission and associated skin and pharyngeal infections. GAS has been reported to survive on inanimate objects for more than six months [113]. Removing dust, handwashing, and disinfecting surfaces are used as control measures in hospitals affected by GAS outbreaks [114,115,116]. It therefore seems plausible that an absence of these measures, a lack of laundry facilities, and low frequency of bedding changes could potentially increase infection risk.

Hygiene is well established as an important determinant of GAS pyoderma (a potential cause of ARF). An intervention study in squatter settlements in Pakistan found that improved handwashing and the use of soap was associated with a decline in impetigo compared with control neighbourhoods [117]. Regular bathing, including swimming in chlorinated pools, may also be protective [118].

Risk factor studies have not reported on the role of household washing and laundry facilities in ARF or RHD. One case-control study in Bangladesh found an association between RHD and the use of poorer quality surface water compared with ground water supply [60].

#### 3.4.4. Housing Conditions, including Tenure, Damp and Cold

In NZ, rental housing is in relatively poor condition compared with owner-occupied housing [119]. Poor housing conditions (e.g., cold, damp, mould) could potentially contribute to an indoor environment that increases the risk of GAS transmission. Due largely to the strong sociodemographic pattern of ARF incidence, an aetiological link has been drawn between ARF development and poverty (with its associated risk factors, including poor housing conditions and overcrowding).

A small number of studies have reported on the relationship between housing conditions and the distribution of GAS. A cohort study of primary school students in Singapore found that GAS incidence was significantly higher in social housing compared with private housing [107]. A cohort study in India of children aged 5–15 years living in peri-urban slums found a significantly higher incidence of GAS in households that lacked a kitchen compared with those that had a kitchen [106]. In an outbreak of GAS in a UK boarding school, the GAS attack rate was significantly higher in poorly ventilated dormitories compared with those that were well-ventilated [120].

Three higher quality case-control studies of ARF that reported multivariate results investigated aspects of housing conditions. One study carried out in Yugoslavia identified several housing risk factors that were significantly associated with ARF, including home dampness and a change in place of residence in the last five years [45]. A Bangladesh study found that ‘substandard housing’ was associated with a greater than three-fold elevated risk of ARF in the multivariate analysis [48]. Another Bangladesh case-control study identified an association with building materials (brick walls) and urban residence for both ARF and RHD [60]. One RHD study found an association with ‘substandard housing’ which became non-significant in multivariate analyses [73].

Cold, damp and mouldy homes have been associated with poor respiratory health [121]. This environment could potentially support the transmission of bacterial pathogens such as GAS [122]. Contributing factors could include lack of insulation, heating and ventilation and the use of unflued gas heaters [123]. The pilot for the NZ risk factors study found that the majority (75%) of cases lived in rental housing, a markedly higher proportion than the general population [112]. In addition, the pilot study reported that most ARF cases (76%) were exposed in the 12 months preceding their ARF diagnosis to at least one of the following: damp walls or ceilings; mould; or a musty smell in the bedrooms or living areas of their home [112]. Most (82%) reported one or more indicators of exposure to cold housing and some had measures of fuel poverty (e.g., 22% had power cut off or prepaid meters running out).

#### 3.4.5. Environmental Tobacco Smoke Exposure

Environmental tobacco smoke (ETS) is an established risk factor for respiratory infections and is a plausible risk factor for ARF [124]. There are several hypotheses around how exposure to ETS may increase ARF risk in susceptible individuals. For example, smokers harbour increased quantities of bacterial pathogens, including GAS, in their oropharyngeal cavities, so may be more exposed to this organism [125]. ETS does not appear to have been investigated as a risk factor for ARF in any published studies. The pilot study investigating housing conditions of ARF cases in NZ in 2012–2013 showed that the majority of cases (71%) lived in homes with at least one smoker [112].

#### 3.4.6. Exposure to Animals, Biting Insects and Skin Injuries

Humans are the natural hosts and reservoir of GAS infection [126]. However, domestic cats and dogs have, on rare occasions, been identified as GAS carriers [127], although no studies have firmly identified exposure to them as a risk factor for GAS infection or ARF [128]. One study identified GAS carriage in the eye secretions of two of 61 pets living closely with people recently diagnosed with GAS infection (pharyngitis and skin infections). In these cases, both the pet and the human GAS isolates were of the same T-type, implying that the animals (a dog and a cat) may have been the source of the infection or may have contracted it from their owners [129]. Another study involving 149 domestic cats and dogs failed to identify GAS carriage in any of the 371 swabbed body sites [130]. A cross-sectional study in the UK identified contact with cattle and drinking unpasteurised milk as protective against ARF [47]. Insect bites, including from fleas, are a potential contributor to GAS exposure through breaks in the skin. However, no studies investigating GAS transmission in relation to insect exposure or bites were identified in the literature.

### 3.5. Healthcare Access and Risk of ARF

#### 3.5.1. Health Literacy

Knowledge of ARF and appropriate use of health services for treatment of sore throat infections is widely accepted in the literature as being an important aspect of primary prevention [131,132,133,134,135,136]. A Yugoslavian case-control study found that low-education level of the mother was associated with ARF [45]. Similarly, RHD (but not ARF) was associated with maternal illiteracy in a Bangladesh case-control study [60].

Increasing awareness of the need for primary prevention in children with symptoms of pharyngitis, both in communities where children face a high-risk of ARF and among the health professionals who work with them, was a major focus of the NZ Rheumatic Fever Prevention Programme (RFPP) [137]. Various public health campaigns have promoted messages such as ‘sore throats matter’ and ‘sore throats can break a heart’ through a variety of media. The school-based programme has also contributed to increased public understanding of ARF and its causes [92]. It is difficult to disentangle the effects of awareness raising strategies from that of other aspects of the RFPP when considering its impact on ARF incidence.

Enhancing awareness of ARF and its prevention was a major aspect of the Cuban intervention programme that occurred from 1986–1996, during which period ARF incidence declined 7.4-fold [138]. Martinique and Guadeloupe also received a ten-year ARF control and prevention intervention which included educating healthcare professionals on ARF and emphasising the importance of primary prevention in schoolchildren. This programme also coincided with a significant (>70%) reduction in ARF incidence [139].

Generally, health education can empower people to take responsibility for their own wellbeing. Logically this may be an important determinant of ARF. A systematic review which aims to evaluate the effectiveness of health education in regard to ARF prevention is currently underway [140].

#### 3.5.2. Healthcare Access

Preceding GAS infections are known initiators of ARF. Thus, effective treatment of such infections has the potential to interrupt the development of this illness. It is logical to conclude that access to suitable primary care services that diagnose and treat GAS throat and skin infections should be a protective factor for ARF [141]. Poor access to healthcare is firmly associated with low SES [142], which itself is associated with ARF.

The widespread availability of comprehensive care clinics in Baltimore [143], Cuba [138], and Costa Rica [144] coincided with significant reductions in ARF incidence rates documented in ecological evaluations. ARF remains relatively common in many populations where access to healthcare is a known public health problem [37,145], including NZ [146].

Effective management with penicillin of presumed or proven GAS pharyngitis in populations at high-risk of developing ARF is considered a key evidence-based strategy in ARF prevention. Treatment of GAS pharyngitis with injectable long-acting penicillin was established in randomised controlled trials as a means to prevent ARF in closed populations, such as military barracks in the 1950s and 1960s. A meta-analysis of such trials found ARF was reduced by 80% (RR 0.20, 95% CI 0.11–0.3) when treatment was provided [147]. Another meta-analysis also identified a two-third reduction in the occurrence of ARF in the month following pharyngitis when antibiotic treatment was provided (RR 0.27, 95% CI 0.12–0.60) [148].

In community settings, there has been a lack of robust evidence to support treatment of GAS pharyngitis, either with injectable or oral penicillin, to prevent first presentation ARF. Improved access to primary healthcare clinics over a decade was considered responsible for the ARF decline in a formerly high risk inner city US setting [143]. Other evidence is ecological with school-based and/or community-based before and after interventions, suggesting that ARF control is possible using antibiotics as a primary prevention [37]. In NZ, a cluster randomised trial of improved access to oral penicillin treatment of GAS positive sore throats using school clinics conducted between 1998 and 2001 identified a non-significant decline in ARF cases [149].

Two randomised clinical trials have produced evidence indicating that prompt treatment of GAS pharyngitis with antibiotics may actually suppress the immune response, leaving the host more susceptible to GAS pharyngitis relapse and recurrence [150,151]. According to one theory, prompt antibiotic therapy may remove the pathogen before an immune response capable of effective and prolonged protection is generated [128]. However, these observations have been challenged and other trials have not replicated these findings [128,152].

As noted in the introduction, the NZ Government developed the RFPP with the goal of reducing the incidence of ARF by two-thirds, to 1.4 per 100,000 by mid-2017 (based on initial ARF hospitalisations) [153]. The major component of the RFPP is the school-based sore throat management programme, which aims to prevent ARF through timely diagnosis and treatment of GAS pharyngitis. This component is delivered to children attending primary and intermediate schools (aged 5–13 years) in areas with the highest rates of ARF. Operation of the school-based programme, including the child population coverage and the level of local general practitioner involvement, varies across the North Island of NZ.

The second major component of the RFPP is improved sore throat management in primary care. This component includes provision of ‘sore throat clinics’ and education for health practitioners. The RFPP has other elements, including improving health literacy for youth and families concerning sore throats and rapid referral to services designed to improve housing conditions for families at high risk of ARF. National guidelines for the treatment of pharyngitis in primary care settings were made available (www.heartfoundation.org.nz).

An evaluation of the school-based RFPP did not demonstrate a significantly decreased ARF incidence in children exposed to the programme overall [137]. During the period of operation of the RFPP, the NZ rate of ARF declined from 4.0 per 100,000 in 2012 to 3.4 per 100,000 in 2017 (Ministry of Health website, http://www.health.govt.nz/about-ministry/what-we-do/strategic-direction/better-public-services/previous-bps-target-reduce-rheumatic-fever). However, a published before-and-after evaluation of school clinics in South Auckland (Counties Manukau DHB) which contains the highest concentration of ARF cases (Supplementary Table S1) reported a 58% (*p* < 0.008) decline in first presentation ARF incidence following two years of the school clinic programme. This was in a geographically distinct area where approximately 90% of high-risk children had access to a clinic. A parallel decline in cross-sectional pharyngeal GAS prevalence was also demonstrated [149].

Treatment of skin infections, including impetigo and scabies, offers a potential intervention for reducing the risk of ARF but needs further investigation [154,155,156].

### 3.6. Health Status, Nutrition and Risk of ARF

#### 3.6.1. Health Status

Only a few aspects of physical health history have been identified as being associated with an increased risk of ARF. One suggested area is being under-weight as a consequence of poor nutrition (see ‘Nutrition’ below). A related area is poor oral health (see ‘Oral health status and services’ below). There are also some associations with perinatal factors (see ‘Pregnancy and birth’ below). Two case-control studies in Brazil also found an association between ARF and mental health, notably obsessive-compulsive spectrum disorder [55] and generalised anxiety disorder [56]. These associations suggest related underlying etiologic mechanisms common to both conditions.

#### 3.6.2. Oral Health Status and Services

Some observational studies have found an association between dental caries and ARF, including a case-control study in Philadelphia carried out in 1949 [39]. In 1938, a Canadian dentist noted that around 95% of ARF and bacterial endocarditis cases presented with advanced dental caries [157]. Both RHD and poor oral health are more prevalent in deprived populations. They are likely to occur together, and it is possible that they share a common bacterial aetiology. GAS have been isolated in dental plaque [158]. Oral health and dental microbiota are linked to endocarditis [159] and a multitude of systemic diseases [160]. A review of blood cultures in a case series of endocarditis cases has identified oral GAS as pathogens involved in some cases [161,162].

It has been suggested that the association between poor oral health and ARF is linked to a common exposure, namely sugar [163]. A cohort study of 20,333 children in Auckland who were free of RHD at enrolment were followed for a mean of five years. A total of 96 developed ARF or RHD. Those with five or more primary teeth affected by caries were 57% (95% CI: 20% to 106%) more likely to develop ARF or RHD compared with those who were caries-free [61]. There is some biological plausibility for high-sugar intake being a risk factor for ARF. GAS organisms can ferment sucrose (table sugar) and fructose (which, along with glucose, forms the disaccharide sucrose) [164,165]. A high sucrose intake may well enhance conditions that promote the growth of GAS in the oral cavity, increasing the likelihood of developing GAS pharyngitis and thus ARF [163]. A study in Bangladesh identified not brushing teeth after a meal as being significantly associated with ARF [60].

However, dental caries is a multifactorial condition that does not correlate perfectly with sugar intake. Early childhood caries is strongly associated with low socioeconomic status (SES) of parents [166], which may also contribute to ARF risk through multiple mechanisms. At an ecological level, the incidence of dental caries and ARF/RHD show an inconsistent association. In 12-year old children, experiencing dental caries is most common in South America, Eastern Europe, India, parts of Africa and the Middle East [167]. Despite this distribution, reported cases of ARF in South America and Eastern Europe have declined dramatically over the previous century, yet ARF continues to be prevalent in India, Africa and much of the Middle East. South East Asia demonstrates a considerable burden from ARF [168], but generally shows a slightly lower incidence of dental caries compared with other low to middle income regions [167].

If oral health is an important factor for ARF, then access to oral health services is relevant. There are, however, no published studies demonstrating that these services influence ARF rates [163].

#### 3.6.3. Nutrition

Several aspects of nutrition could potentially contribute to ARF risk, including overall nutritional status and inadequate intake of micronutrients, such as vitamin D. Two higher quality case-control studies have reported multivariate analyses of nutritional risk factors for ARF, one in Yugoslavia [45,46] and one in Bangladesh [49,50]. Both reported an association with low body weight. A cross-sectional study of RHD in the Congo identified both low BMI and low birthweight as associated with the risk of ARF in multivariate analyses [67]. Other cross-sectional studies of RHD in India [73] and Fiji [71] identified low height, weight, and BMI as significant risk factors in the univariate, but not multivariate analyses.

The Bangladeshi ARF case-control study reported an association of ARF with reduced albumin and iron stores [50]. It also observed an association with low consumption of certain foods, notably eggs, which persisted in the multivariate analyses. This study also documented an increased risk of ARF in children with a reduced upper arm circumference which is considered indicative of protein-energy malnutrition [49].

The immunological roles of Vitamin D are increasingly recognised [169]. There are no reports of its association with ARF, but one study found an association between serum vitamin D levels less than 20 ng/mL and recurrent GAS tonsillopharyngitis (OR 1.62, 1.51–1.76) [170]. An earlier study noted an association between the prevalence of an allele of a Vitamin D binding protein (Gc2) polymorphism and ARF in an Arab population [44]. In NZ low Vitamin D levels are more common in Māori than non-Māori [171]. Iron deficiency is also more prevalent in Māori than non-Māori [171]. In NZ, the dominant nutritional concern for children with the highest rates of ARF (Māori, Pacific) has become obesity [172,173]. Obesity can occur alongside micronutrient deficiencies in situations where there is over consumption of relatively poor quality food.

### 3.7. Wider Modifiable Social Determinants, including Income and Education

Socioeconomic position is a key determinant that influences multiple potential risk factors for ARF and RHD (Figure 8). This determinant can be measured on the basis of SES, income poverty, living standards, and deprivation [34]. The most widely used measure of social deprivation in NZ is the area-based NZDep. The NZDep13 index is based on nine variables from the 2013 census which reflect eight dimensions of deprivation [33]. NZDep provides a deprivation score for each meshblock in NZ (median of approximately 81 people in 2013). In addition, an index of individual deprivation (NZiDep) has also been developed [174]. This index is based on eight simple questions that can be administered using a questionnaire.

At an ecological level, the distribution of ARF and RHD is clearly associated with socioeconomic deprivation across Africa, the Americas, Asia, Europe and the Pacific [19,21,168,175,176,177,178,179]. Studies of risk factors for ARF and RHD have also generally found an association between socioeconomic factors and ARF and RHD (Supplementary Table S2). Among the higher quality case-control studies, ARF has been found to be associated with low maternal education in Yugoslavia [45]. Case-control studies of RHD found an association with maternal illiteracy in Bangladesh [60] but an inconsistent association with education level in Uganda [76]. A cross-sectional study of RHD reported an association of RHD with lower SES in the Democratic Republic of the Congo [67], whereas a cohort study in New Caledonia found that the association with maternal education disappeared in the multivariate analysis [79]. It has been suggested that the threshold where higher SES would be associated with lower RHD prevalence has not been reached in low-income countries [180].

### 3.8. Predisposing Host Factors that Are Inherited or Act during Early Development

Host factors include those that are largely fixed (such as demographics, ancestry and genetics) and those that are influenced by perinatal events and early childhood exposures.

#### 3.8.1. Demographic Risk Factors

The risk of ARF is strongly influenced by specific demographic factors, particularly age and ethnicity (which is discussed further under ‘ancestry and genetic factors’ below). ARF is rare in children under four years of age, incidence rises to a peak at around nine to 12 years, and then declines in those over 20 years of age [181]. This very specific age-group vulnerability to ARF suggests a strong contribution from maturation processes in the immune system [182]. Some studies reported a higher risk for females, particularly for RHD in low- and middle-income countries, which may be at least partly associated with healthcare seeking behaviours resulting from pregnancy [60,68,69].

#### 3.8.2. Ancestry and Genetic Factors

Inherited genetic variants are likely to be important in ARF susceptibility but are poorly understood [183]. Familial rheumatic fever has been described for more than a century [37]. An association with family history was observed in a case-control study conducted in Yugoslavia in 1982 [45]. Further evidence for a genetic component comes from the finding that the pooled proband-wise concordance risk for ARF is 44% in monozygotic twins and 12% in dizygotic twins, with an estimated heritability of 60% [183].

The high degree of variation in ARF/RHD incidence in relation to ethnicity suggests that genetic factors may affect susceptibility, along with the inter-generational legacy of colonisation. The elevated risk for Māori and Pacific children in NZ is very marked, even after stratifying for deprivation [26] (see Figure 6). Countering the view that genetic factors are the dominant determinant of ARF distribution is the observation that high rates of ARF were observed in all ethnicities earlier in the 20th century (and before) internationally [20] and in NZ [184].

A number of genetic polymorphisms have been significantly associated with ARF and RHD. The genes identified include IFN-*γ*, ACE, FCN, Fc*γ*RIIA, TLR-2, and HLA (supplementary Table S2) [51,52,57,59,70]. Different HLA class II antigen associations with ARF have been observed in several populations. The HLA class II genes encode cell-surface proteins that present antigen to the T-cell receptor (TCR) and trigger adaptive immune responses. One previous study associating HLA haplotypes with ARF and RHD found that a minor increase in HL-A3 and -A8 in disease [64]. The HLA class II region is strongly associated with a wide spectrum of autoimmune disorders [185], including rheumatoid arthritis, where a specific group of HLA-DRB1 alleles (called the ‘shared epitope’) increases risk two-to-three-fold. In individuals exposed to smoking (and positive for anti-cyclic-citrullinated peptide antibodies), the increased risk mediated by HLA-DRB1 is magnified to 20-fold [186,187,188,189,190]. This widely replicated gene–environment interaction has allowed insight into the aetiology of rheumatoid arthritis—smoking increases risk by causing citrullination of proteins, which are better able to activate the immune response in the presence of HLA-DRB1 shared epitope [188,189]. Considering that ARF, like rheumatoid arthritis, has an autoimmune aetiology it is possible that an HLA class II gene–environment interaction could be present that might contribute to the disparity in ARF incidence between Māori/Pacific and other NZ ethnic groups.

A small study (204 RHD cases, 116 rheumatoid arthritis controls) comprised of participants with NZ Māori and Pacific ancestry reported an association of a genetic variant in the IL6 promoter (rs1800797 (-597G/A)) with RHD, and association of an IL1RN variant (rs447713) with the severity of carditis [80]. A study conducted in Pakistan also identified associations with IL6 [78].

To overcome the problems of small datasets, international multi-country trans-ethnic genome-wide association study (GWAS) meta-analyses are now underway to identify genetic determinants of RHD susceptibility. The first published GWAS of RHD was based on 2852 individuals recruited in eight Oceanian countries. It identified a novel susceptibility signal in the immunoglobulin heavy chain locus [82]. More recently, a GWAS in the Australian Aboriginal population identified the HLA-DQ locus as being the strongest genetic marker associated with RHD, with the data supporting a role for cross-reactivity with GAS epitopes in aetiology [81].

#### 3.8.3. Pregnancy and Birth

There is evidence that the prenatal period may be important in terms of future susceptibility to some infectious diseases [191]. Preterm newborns have suppressed immune function [192]. In a cohort of more than 10,000 newborns, both low birthweight and preterm birth were associated with a near-70% increased risk of subsequent hospitalisation with an infectious disease during childhood [193]. Similar observations were found in a large Swedish study in which children born both preterm and with a low birth weight had about a 50% increased risk of hospitalisation with an infectious disease during adolescence or early adulthood [194]. A Danish study found that children born preterm with low birth weight, or a low Apgar score were substantially more likely to be hospitalised with pneumococcal disease [195]. In the United Kingdom, low birth was associated with a doubling of enteroviral meningitis risk [196]. A large Danish case-control study observed that children born preterm were at increased risk of meningococcal disease in the first year of life, while those with low birth weight had increased risk of this disease throughout their childhood [191].

Few studies have reported on perinatal risk factors for rheumatic fever. A Finnish case-control study identified umbilical cord length as a significant risk factor for RHD. The authors postulated that increased length puts more stress on the foetal heart, making mitral valves more vulnerable to rheumatic processes, with an increased risk of occurrence and death from RHD demonstrated [77].

#### 3.8.4. Autoimmunity

ARF is an autoimmune response to GAS infection [38]. Despite the relatively well-documented histopathology, the mechanisms that trigger ARF remain poorly understood. The prevailing hypothesis is that molecular mimicry exists between GAS antigens and host tissue that generates cross-reactive antibodies and T cells [197]. However, there continues to be on-going debate as to the real role mimicry has in ARF pathogenesis [198]. There is still no consensus on which antigens initiate the autoimmune response, nor a clear understanding of the immune cell profile in ARF. There is a lack of studies that have applied contemporary, high-definition immune-profiling technologies to ARF [199]. The application of such technologies should provide a better understanding of pathogenesis, which is crucial to the development of new interventions for ARF, both preventative and therapeutic [2].

It is thought that multiple repeated exposures to GAS ‘prime’ the immune system prior to development of ARF; however, empirical evidence for this hypothesis is limited. A small laboratory study conducted in NZ used GAS T-antigens to investigate this theory [200]. T-antigens are type-specific antigens that fall into 18 major clades or T-types. This study found that in each of the ARF case sera tested, at least two distinct GAS exposures were detected and no cases shared the same pattern of T-type reactivity. These findings provide some support for the immune-priming hypothesis. However, multiple factors could theoretically affect development and priming of the immune system resulting in ARF.

### 3.9. Organism Factors, including Infectious Co-Factors

#### 3.9.1. Exposure to Group A Streptococcus (GAS)

Exposure to GAS is considered necessary for the development of ARF and evidence for this exposure is a prerequisite for ARF diagnosis [201]. GAS is commonly typed by sequencing the 5’ end of the *emm-*gene, which encodes the M-protein, with molecular epidemiological studies having identified over 200 *emm*-types to date. Historically, certain *emm*-types were epidemiologically associated with epidemic ARF, including *emm* 1, *emm* 3, *emm* 5, *emm* 6, *emm* 14 and *emm*18 [202,203,204]. This observation resulted in the concept of ‘rheumatogenicity’, whereby strains associated with outbreaks of ARF (in the US in particular) were thought to have a greater propensity to cause disease than other strains.

However, contemporary studies in settings with high endemic ARF disease burden have suggested that a diverse array of *emm*-types are likely to play a role in the epidemiology of ARF [10,205,206,207]. A recent study of GAS isolates obtained from ARF cases in NZ found few of the so-called rheumatogenic types, and a diverse range of *emm*-types previously associated with pyoderma in other settings [89]. This finding suggested that skin infections may have a role in development of ARF. This conclusion was further supported by a contemporary study of skin and throat isolates from high-risk children in NZ that found the *emm*-types associated with skin infections were similar to those from ARF cases [208]. However, the restriction of genomic analysis to the *emm*-gene is somewhat limiting and with such a diverse range of ARF associated *emm*-types now identified, understanding the rheumatogenic potential of GAS will require further investigation. Future work using whole genome sequencing may enable potential genomic associations between ARF-linked GAS strains and ARF to be elucidated.

#### 3.9.2. Exposure to Potential Infectious Co-factors

Other common bacterial infections could potentially increase the risk of either GAS infection or ARF. *Staphylococcus aureus* infection is commonly found in association with GAS as a cause of skin infection. The epidemiology of skin infections, many caused by *S. aureus*, has some parallels with ARF, with particularly high rates in Māori and Pacific children living in relatively deprived neighbourhoods [209].

Although not extensively studied, there is little evidence of viral infection acting as a synergist in the development of ARF [210]. It is possible that eradication of normal oral microflora, especially α-haemolytic Streptococci, from the oral cavity (perhaps due to prior *β*-lactam antibiotic use) increases susceptibility to GAS infection. The presence of such microflora has been shown (although not consistently) to help protect against GAS infection through bacterial interference [128]. A recent trial of the use of the oral probiotic *S. salivarius* K12 (K12) found that it was associated with only a modest non-significant decline in GAS culture-positive sore throats when given at school [211].

## 4. Study Aims and Research Questions

The primary aim of this study was to identify potentially modifiable risk factors for ARF with the ultimate goal of producing robust evidence to support policies and programmes to decrease rates of ARF in high-risk NZ populations. The specific aims were as follows:

1. Identify potentially modifiable environmental risk factors for ARF, notably household crowding and bed-sharing, poor housing conditions, and ETS.
2. Establish whether access to healthcare, including sore throat treatment and related health literacy, is protective for ARF.
3. Establish whether current or recent skin infection is associated with an increased risk of ARF.
4. Establish whether poor oral health is associated with an increased risk of ARF.
5. Identify potentially modifiable host and nutritional factors for ARF, such as vitamin D deficiency, anaemia and high consumption of sugar-sweetened beverages.
6. Contribute to identifying immunological factors associated with an increased risk of ARF.
7. Establish whether a positive family history and the HLA-DRB1 locus or other plausible genetic markers are associated with ARF.
8. Establish whether specific GAS organisms are associated with ARF.

The aims and study design allow us to investigate the following research questions:

- Are there modifiable environmental exposures contributing to an increased risk of ARF, notably household crowding, bed sharing, poor indoor environments (e.g., cold, damp, mouldy), fuel poverty, tobacco smoke exposure, limited resources for washing and teeth cleaning, inadequate protection from insect bites and fleas?
- Are there modifiable host and nutritional factors contributing to an increased risk of ARF, notably vitamin D deficiency, high consumption of sugar-sweetened beverages, low consumption of fruit and vegetables?
- Is ARF associated with skin infections, which might suggest the need to treat such infections as part of ARF prevention programmes?
- Are there knowledge, attitudes and behaviours associated with a decreased risk of ARF, notably health literacy around treatment of sore throats and skin infections?
- Is good access to health services protective for ARF?
- Is there a large proportion of ARF cases with a history of sore throat who did not receive treatment for this, suggesting the potential for improved pharyngitis treatment?
- Is participating in school-based sore throat management programme protective of ARF?
- Does the distribution of GAS *emm*-types that we observe to be associated with ARF differ from those *emm*-types circulating in the wider child population?
- Are there specific immunological markers of increased susceptibility to ARF?
- Is family history of ARF/RHD a risk factor for ARF?
- Do environmental exposures interact with inherited factors (e.g., HLA-DRB1 locus) in a non-additive (multiplicative) way to explain disease distribution?

## 5. Study Design

The study design is a prospective population-based case-control study. Considerable effort was put into considering potential risks to the study effectiveness and minimising these risks (see Supplementary Table S4).

### 5.1. Study Population

Since the vast majority of ARF cases occur in the North Island of NZ, this study will be restricted to that island. Specifically, cases and controls will be recruited from the 11 DHBs in which we might expect five or more cases of ARF over a two-year period (see Epidemiology section).

Cases will meet the standard NZ case-definition for new cases of ARF [212] (Table 1). Comparison with matched controls will be used to investigate risk factors that might explain why some similar individuals (same ethnicity, age, sex, deprivation level, DHB) develop ARF and others do not. Cases will also be compared with community controls (from the New Zealand Health Survey (NZHS)) to give an understanding of the contribution of major socio-demographic factors to the risk of ARF.

### 5.2. Cases

#### 5.2.1. Case Recruitment

The case recruitment process is shown in Supplementary Figure S1. Cases will be identified by the diagnosing paediatrician or adult physician at hospitals within the participating study areas. These clinicians will be encouraged to approach eligible cases or their parent/caregiver (if the case is less than 16 years old) and seek preliminary consent for the study team to make contact regarding the study. The interviewing service (CBG Health Research Ltd.) organise a face-to-face meeting where the study is discussed, concerns addressed, and written consent obtained.

A range of additional mechanisms will be used to maximise recruitment and ensure this is was done in a timely manner. These measures included: a part-time Recruitment Coordinator (FCM) employed specifically to assist with participant recruitment in the Auckland region; a monthly electronic newsletter about the study disseminated widely across the health sector; periodic review of case recruitment numbers compared with surveillance data (ARF is a notifiable condition) to identify non-referral patterns and act on them.

#### 5.2.2. Case Inclusion and Exclusion Criteria

Cases will be assigned diagnostic categories based on 2014 NZ guidelines which are essentially a modified version of the Jones Criteria [212] (Table 1). The only change made for this study will be to switch to using the upper limit of normal (ULN) cut-off criteria for streptococcal titre levels (antistreptolysin O (ASO) and anti-deoxyribonuclease B (ADB)) that are used in Australia and internationally and which are lower than those in the NZ Guidelines for Rheumatic Fever [213].

A summary of the case inclusion and exclusion criteria for the study is shown in Table 2. Cases presenting only with chorea will be excluded. The rationale is that these cases have a very different time course from other cases of ARF. Chorea may present many months after the acute GAS infection, instead of a few weeks like most ARF cases, and may sometimes be the only presenting symptom of ARF [214,215]. For similar reasons, cases presenting with indolent carditis will also be excluded. This is ‘carditis of insidious onset and slow progression with evidence of inflammatory disease as distinguished from chronic RHD’ [212]. Given the increased likelihood of recall bias caused by this delay, the inclusion of such cases is unlikely to provide additional useful knowledge about recent environmental exposures contributing to the risk of ARF.

#### 5.2.3. Case Review

Patient data will be collected at intervals during the study by a clinically trained researcher and compiled into a spreadsheet. All cases will subsequently be reviewed by a case review panel of clinicians (paediatric infectious diseases, general paediatricians and a paediatric cardiologist) experienced with ARF diagnosis, in order to categorise patients by diagnostic certainty [212,216]. Based on these data and in line with ARF diagnostic categories (Table 1) and the study inclusion and exclusion criteria (Table 2), the case review panel will categorise cases as eligible for inclusion in the study or otherwise.

### 5.3. Controls

The study has two control groups: matched controls and community controls.

#### 5.3.1. Matched Controls

These will be children and young adults who are matched to the cases for their socio-demographic characteristics, location, and month of recruitment. Matched controls have participated in the NZHS and have consented to further follow-up. This pool of potential controls is continually replenished from the NZHS, which is a rolling population-based survey that includes a sample of 14,000 participants each year [217]. Those consenting to follow-up remain in the recruitment ‘pool’ for up to two years after their initial survey. When an ARF case has been identified and given consent to participate, matching controls will be identified. They will be randomly matched to each case by age (within one-to-two years), ethnicity (prioritised), gender, deprivation (NZDep decile) and DHB. They will also effectively be matched by time-period as the control interviews will be conducted within one-to-four weeks of the case interview to control for possible seasonal effects. Controls that have ever had ARF or RHD will be excluded. Age matching uses a calliper approach (i.e., ± two years) rather than an age band.

Occasionally there may be a lack of controls that match cases according to all strata. If so, the matching criteria will be relaxed until suitable controls are found. Criteria will be relaxed sequentially in the following manner: gender; age group to ± four years; NZDep criteria to ± two NZDep deciles, then ± three NZDep deciles; DHB to allow controls from any DHB in the same general region. We will not loosening the ethnicity criteria (prioritised level-one ethnicity, i.e., Māori, Pacific, Asian, European/other).

The matched controls will be recruited at a 3:1 ratio of controls to cases, giving an estimated final sample size of at least 120 cases and 360 controls. Once matched controls are identified, the Study Coordinator will organise a face-to-face meeting where the study is discussed, concerns addressed and written consent obtained.

#### 5.3.2. Community Controls

These controls consist of the NZHS dataset and allow the study to investigate how ARF cases compare with NZ children and young people more generally. The NZHS aims to interview a weighted sample of approximately 14,000 adults and 5000 children each year. It uses ‘a multi-stage, stratified, probability-proportional-to-size (PPS) sampling design’ and ‘a dual-frame approach’ with respondents selected from an area-based sample and a list-based electoral roll sample [217]. This method is used to increase the sample sizes for Māori, Pacific and Asian ethnic groups.

For these controls, we will use the sample of all children aged 4–19 years who were surveyed as part of the NZHS over four successive times periods from July 2013 to June 2017 (i.e, 2013–2014, 2014–2015, 2015–2016, 2016–2017). This control group includes an estimated 19,500 children. The content of these surveys varies from year to year, with some core sections and some additional modules added in different years (e.g., the 2013–2014 survey included additional modules on housing, NZiDep, second-hand smoke, and long-term health conditions). These interviews have already taken place and the data are part of the NZHS. Use of this set of controls allows us to investigate the association of ARF with the socio-demographic factors and also give us large numbers and hence greater power to investigate associations with exposures which were measured with the NZHS.

## 6. Data Sources and Specimen Collection

Once recruited into the study, cases and matched controls (or their parent/caregiver if aged less than 16 years old) will be interviewed using a study questionnaire, as described below. Blood, throat and nasal swabs, and hair specimens will also be obtained on a sub-sample of study participants, as described below. The scope of data collected by the study is shown in Supplementary Table S3.

### 6.1. Study Questionnaire

#### 6.1.1. Content

The study questionnaire was developed to obtain data on key study variables and exposures (Supplementary Table S3). Considerable effort was invested into content development, with the rationale for each question discussed and debated within the study team. The study questionnaire drew on existing questionnaires where appropriate to maximise comparability. In particular, questions used in the NZHS [218], Southern Hemisphere Influenza Vaccine Effectiveness Research and Surveillance (SHIVERS) study [219], the Health of Occupants in Mouldy Environments (HOME) study *(Dr. Caroline Shorter, University of Otago, 12 February 2015, personal communication)*, the Youth 12 Survey [220] and a recent survey of housing conditions in hospitalised children [221]. A component of the study questionnaire (focused on housing conditions and service utilisation) was tested in a pilot study with a sample of 55 ARF cases (and their caregivers) who were interviewed by phone [112].

It was important that the questions be designed to minimise bias, notably various forms of information bias. For example, particular emphasis was placed on ensuring that (wherever possible) the design and operation of the study would not introduce differential recall of key exposures by cases and controls.

#### 6.1.2. Pre-testing and Pilot Testing

Cognitive testing was carried out for key questions, particularly where language or sensitive subject matter was involved. The draft questionnaire was pilot-tested on a sample of cases (*n* = 10) and controls (*n* = 16) to assess its performance, including interview length. The final version was modified accordingly to ensure that the interview was no more than an hour in length.

#### 6.1.3. Selection of Proxy Interview Subject for Children

Consent to take part in the study for those under 16 years of age will be provided by a parent or other legal guardian. This process needs to ensure that the main caregiver is selected and interviewed in a similar way for cases and controls. This issue is particularly important for questions in which we specifically elicit characteristics of the interview subject rather than the child; for example, caregiver education level.

#### 6.1.4. Selection of Housing Environment for Children

The interview process needs to ensure that the questions relating to the subject’s home environment select the house in a consistent way across cases and controls. This is mainly an issue for children in a ‘shared-care’ situation where they move between two (or more) places. The focus should ideally be on the house where they spend most time and where their main caregiver lives. We have included a question to identify those children who regularly stay at more than one house so that we can assess the impact of this potential area of exposure misclassification.

#### 6.1.5. Selection of Time-period

Most questions in the study questionnaire ask about exposures that are relatively stable (i.e., not changing over periods of a few weeks to months) or ask about ‘usual’ rather than ‘specific’ exposures. A small number of questions ask about exposures that may vary, such as the number of people in the house. It was necessary to create a specific reference period for cases and controls to answer these questions: for cases, this is the period before they (or their child) got sick with ARF, while for controls, this is the period before the interview. This approach does produce potential for recall bias, since cases and controls are not being asked to recall information for the same time-period. In addition, cases benefit from having a memorable event to assist with their recall (i.e., their illness), which the controls do not have. This difference may also be a source of recall bias—one which is difficult to avoid. We have attempted to minimise these biases by (a) not including cases who were hospitalised more than four weeks prior to the interview and (b) minimising the number of questions that ask for recall within this specific time-period.

#### 6.1.6. Household Composition

The definition of household membership was given careful consideration during questionnaire development. The general approach in the NZHS is to ask for ‘the initials, age, and gender for all persons usually residing in the house’. Any person who usually resides in the household, even if they are away on the day of the interview (e.g., temporarily overseas), is recorded on the household list. However, people who are currently away from home and will not return home in the next four weeks are excluded. We have chosen to follow this protocol for the current study.

### 6.2. Biological Specimens

The specimens that are collected for the study from cases and controls and the associated measurement(s) of interest are shown in Table 3. For logistical reasons (including the timely transportation of specimens from collection locations), collection of these bio-specimens will be restricted to participants from the Northland, Auckland, Waikato and Wellington regions (which include seven out of the 11 DHBs participating in this study)**.** In addition, specimens are only collected from one control per case for logistic and budgetary reasons

### 6.3. Linked Health Information

In addition to questionnaire and specimen data, we also seek permission from participants to obtain linked information from the following sources (Table 4) to investigate exposures more thoroughly and reduce the effects of recall bias.

### 6.4. Data Collection

#### 6.4.1. Interviewer Selection, Training and Quality Assurance

The study interviewers were selected and received specific training for this study, including the content of the questionnaire and methods of sample collection (throat and nasal swabbing, hair specimen collection). A one in 10 sample of participants will be re-interviewed by the Study Coordinator (AC) as a quality check. It is not possible to blind interviewers to the case or control status of subjects as interviews for these two groups generally will take place in different settings and the questionnaires contained different content in some specific areas.

Interviewers will be ethnically matched to the cases so that Māori participants will be interviewed by Māori interviewers and Pacific participants by Pacific interviewers. This step was decided as it is thought that ethnically-matched interviewers help to create a culturally safe interview environment that improves the experience for the subject and the quality of information provided [222].

#### 6.4.2. Interviews

These will be conducted in face-to-face settings, with data entered directly onto a portable computer. Show cards with predetermined response categories will be used to assist respondents. These cards included photographic images to help subjects recognise some specific items (types of skin infection, scabies, ventilation strips and vents, unflued gas heaters). Case interviews will usually occur in hospital immediately after consent is obtained or at home if the ARF case has already been discharged from hospital).

#### 6.4.3. Specimen Collection

The methods used to collect specimens for the study differ slightly between cases and controls. For cases, study interviewers collect nasal swabs and hair samples. The nasal swab will be delivered to a local specimen collection room within 24 hours of the interview. The hair specimens will be stored and subsequently tested. Blood specimens for the study will be taken during routine blood collection rounds while the cases will be taken in hospital, or when the case visits a local specimen collection room (within four weeks from the ARF diagnosis) if already discharged from hospital. It is routine clinical practice to collect a throat swab for GAS culture on hospital admission whenever ARF is suspected. Throat swabbing results are affected by the administration of antibiotics, and thus, the admission swab is likely to provide the most valid indication of the presence of GAS prior to admission.

For matched controls, interviewers will collect throat swabs, nasal swabs and hair samples. Interviewers will also give controls packs containing specimen collection tubes and corresponding request forms to enable the collection of blood samples from local collection rooms.

As mentioned above, specimen collection will be restricted to cases and controls from seven of the 11 DHBs. An exception to this is throat swabs, where efforts are made to obtain specimen from all cases to assist efforts to understand the most common GAS *emm*-types that occur among ARF cases in NZ.

#### 6.4.4. Specimen Storage

A ‘track and trace’ procedure will used to record the physical location of each specimen using an online database. Once specimens are at their final destination, any residual identifiable information is destroyed and only their unique study identifier retained. Study manager (JG) and Principal Investigator (MGB) will retain access to the database via a secure password.

All specimens, except those used for ferritin and Vitamin D assays and hair samples (which will all be destroyed following analysis), will be stored for a period of at least 10 years. Subsequently, blood specimens used for immunological analysis will be stored at the School of Biological Sciences, University of Auckland. Blood specimens used for genetic analysis will be stored at the Department of Biochemistry, University of Otago, Dunedin. GAS isolates and extracted microbial DNA will be stored at ESR at minus 80 °C. The GAS strain collection will subsequently be available to any researchers in NZ who are working in the field of GAS pathogenesis.

### 6.5. Steering Committees and Ethical Review

The study has both a Māori and a Pacific Steering Group each comprised of a cross-section of key stakeholders, including researchers, public health workers, clinicians and community representatives (members are listed in acknowledgements section). These groups reviewed the study questionnaire and protocol to ensure cultural appropriateness. They will also provide culturally appropriate advice as issues arise during the study and will be involved in interpretation and dissemination of research findings.

Participant Information Sheets and consent forms have been translated from English into Te Reo Māori, Cook Island Māori, Samoan, and Tongan.

The Ministry of Health’s Northern-A Health and Disability Ethics Committee (HDEC) approved this study (reference number 14/NTA/53). The study protocol and operation included the requirement for informed consent to participate from participants (or their parent or legal guardian in the case of children under 16).

## 7. Data Analysis

### 7.1. Approach to Data Analysis

The goal of the data analysis is to support effective investigation of the aims and research questions (as described above). There is a particular focus on identifying important modifiable risk factors (as listed in Supplementary Table S3). The analysis attempts to produce ‘…the most accurate (valid and precise) effect estimates obtainable from the data…rather than simply improve the fit…’ [223]. The approach to the analysis is described below.

#### 7.1.1. Descriptive Analysis

Descriptive analyses of the prevalence of each investigated exposure within case and matched control groups will be performed in the first instance. Using univariate analysis, we will compare the prevalence of exposure between cases and matched controls, with suitable statistical testing to detect significant differences (for example, a Chi-square test for categorical variables). Cross-tabulations will be created to present these results.

We will also perform descriptive analyses comparing prevalence of exposure between cases and community controls, for those exposures measured using interview questions that are common to both the current study questionnaire and the NZHS. Again, we will compare the prevalence of exposure between cases and community controls with a suitable statistical test (e.g., Chi-square). These comparisons will be presented as cross-tabulations.

#### 7.1.2. Primary Exposure Analysis

Given that the main aim of this study is to identify potentially modifiable risk factors for ARF, our primary exposures are defined based on whether they could be modified in the short, medium or long term. As such, for the most important categories of risk factors (summarised in supplementary Table S3) we have a priori identified one (or in some cases two) variables which will serve as primary exposures in our analysis for this given category. This primary analysis will use conditional logistic regression to investigate the independent association between these primary exposures and odds of ARF development. We will calculate both crude and adjusted odds ratios (ORs) for each primary exposure, with those factors that are a priori identified as potential confounders of the given relationship included as covariates in these models. For example, measures of socioeconomic deprivation are likely to act as confounders of the potential relationship between household crowding and ARF. All primary exposure analyses will be performed using only data from cases and matched controls. Crude and adjusted odds ratios will be presented as cross-tabulations, with the matched (and community, where applicable) controls as the reference group.

#### 7.1.3. Secondary Exposure Analysis

Each category of risk factors (Supplementary Table S3) has a large number of additional exposures that will be included in the secondary analysis. For these variables, we will first calculate crude ORs. In order to calculate adjusted ORs, a data-driven stepwise approach (with backward elimination) will be taken to determine the variables that should be included in each respective model. Crude and adjusted odds ratios will be presented as cross-tabulations, with the matched (and community, where applicable) controls as the reference group.

#### 7.1.4. Population Attributable Fraction (PAF)

Finally, we will estimate the proportion of ARF in the study population (cases and matched controls) that is attributable to specific exposures, by calculating population attributable fractions (PAFs) for each primary and secondary exposure [224].

### 7.2. Study Sample and Power

Based on a sample size of 100 cases and 300 matched controls (i.e., a 3:1 ratio), there will be sufficient statistical power (at least 80%) at a confidence level of 95% to detect an OR of 2.0 for common exposures (50% prevalence in controls) and an OR of 2.0–3.0 if these exposures are somewhat more common (e.g., 2.2 if 70% prevalence among controls). If we are able to recruit more cases than 100 (and subsequently more controls than 300) within available resources (including time), our study power will increase further: for example, if we achieve a sample size of 120 cases and 360 controls, we will have the power to detect an OR of 1.8 for common exposures (50% prevalence in controls) and an OR of 2.0–3.0 if these exposures are somewhat more common (e.g. 2.0 if 70% prevalence among controls).

### 7.3. Study Operation

Major decisions about the direction of the study will be made by the Principal Investigator (MGB) in consultation with the co-investigators. This process is supported by periodic meetings of the investigators, usually by video or telephone conference. Day to day operation of the study is guided by a Study Management Group consisting of the Principal Investigator (MGB), Study Manager (JG), and Specimen Coordinator (JO). Coordination of interviewers will be carried out by the Study Coordinator (AC) based at CBG Health Research Ltd.

## 8. Dissemination

The investigators will use multiple methods to disseminate study findings. These methods will include the peer-reviewed literature, presentations and discussions in a range of settings and through associated media coverage to inform the wider public.

Research findings will be communicated in a clear, concise, and culturally appropriate manner to Māori and Pacific communities. This process will draw on the expertise of the Māori and Pacific Steering Groups and include press releases and interviews with Māori and Pacific media.

The investigators are already closely involved with delivery of the RFPP and providing public health advice to the Ministry of Health and other health agencies. They include recognised opinion leaders in clinical aspects of ARF and RHD management who will be able to disseminate findings through their clinical networks.

## 9. Conclusions

This study will quantify the association between ARF and a range of potentially modifiable risk factors including: adverse environmental exposures (notably household crowding, bed sharing, poor indoor environments, tobacco smoke exposure); limited resources for personal care (notably washing, teeth cleaning); poor nutrition (notably sugar sweetened beverages); and poor oral health (decayed, missing, and filled teeth).

It will also assess the potential protective effect of good access to primary health care services, including general practitioners, school-based sore throat management services, and oral health care. The study will investigate the association of ARF with preceding treatable infections (notably sore throats, skin infections and scabies) as well as host factors that might assist in targeting ARF prevention services (notably family history of ARF/RHD). All of this information can be used in the short to medium term to guide improved prevention measures and refine current programmes.

In addition, this study is seeking to provide information to better understand the role of circulating GAS types, immunological markers, inherited factors and early life exposures. Such knowledge will assist medium to longer term strategies, including the development of improved diagnostic markers for ARF and an effective GAS vaccine. The results will also contribute to international understanding about the pathophysiology of ARF.

A major strength of this study is that it is taking a comprehensive approach covering organism, host and environmental factors that may be associated with ARF. It has also started with a well-developed model of how these factors may influence the risk of ARF, based on an extensive review of published literature on ARF aetiology. Having closely matched controls as well as community controls will enable the study to examine a wide range of specific environmental risk factors.

The study has important limitations, including potential selection bias of matched controls, information (particularly recall) bias, and only modest precision due to its relatively small size (because ARF is uncommon). Steps have been taken to minimise these sources of error. A major driver in the design of this study has been to ensure that it is highly responsive to the needs of Māori and Pacific peoples.

There are very few high-quality studies that have investigated the aetiology of ARF. The current study aims to fill some of the considerable knowledge gaps that are currently preventing an evidence-informed approach to the prevention of this disease. We are using the methods outlined in this protocol paper to achieve this aim and look forward to reporting our findings in due course.

## Figures and Tables

**Table 1:** Categories of acute rheumatic fever (ARF)—New Zealand (NZ)-modified version of the Jones Criteria.

Diagnosis	Requirements	Category
Initial Episode of ARF	Chorea, or 2 major or 1 major and 2 minor manifestations plus evidence of a preceding GAS infection *	Definite ARF
Initial Episode of ARF	1 major and 2 minor with the inclusion of evidence of a preceding GAS infection* as a minor manifestation (Jones, 1956)	Probable ARF
Initial Episode of ARF	Strong clinical suspicion of ARF, but insufficient signs and symptoms to fulfil diagnosis of definite or probable ARF	Possible ARF
Recurrent ARF	ARF in a case with known past history of ARF or RHD	Recurrent ARF (not eligible for study)

From NZ Guidelines for Rheumatic Fever 2014 [212]. Major manifestations: Carditis (including evidence of subclinical valvulitis/carditis on echocardiogram), Polyarthritis or aseptic monoarthritis (with or without a history of NSAID use), Chorea (can be stand-alone for ARF diagnosis), Erythema marginatum, Subcutaneous nodules. Minor manifestations: Fever, Raised ESR or CRP, Polyarthralgia, Prolonged P-R interval on ECG. * Elevated or rising antistreptolysin O or other streptococcal antibody is sufficient for a diagnosis of definite ARF. A positive throat culture or rapid antigen test for GAS alone is less secure as 50% of those with a positive throat culture will be carriers only. Therefore, a positive culture alone demotes a case to probable or possible ARF.

**Table 2:** Inclusion/exclusion criteria for ARF RISK study.

**Inclusion**
• Definite and probable ARF using NZ criteria (Table 1);
• Recruited within four weeks of hospital admission;
• Aged under 20 years at time of diagnosis;
• Normally resident in study area (one of the 11 North Island DHBs in the study).
**Exclusion**
• Cases presenting only with chorea or indolent carditis;
• Cases with a previous diagnosis of ARF (i.e., recurrent ARF) or RHD;
• Cases outside age group, study area or hospitalised more than four weeks prior to recruitment.

**Table 3:** Specimens collected for study and associated exposure(s) of interest.

Specimen	Measurement(s) of Interest
Throat Swab	Presence/absence of GAS; *emm*-gene typing
Nasal (Anterior Nares) Swab	Presence/absence of *S. aureus*
Blood Samples	Vitamin D; serum ferritin levels; Immunophenotypic-profiling DNA for genetic testing
Head Hair (–2cm Long, Proximal Section)	Nicotine exposure

**Table 4:** Linked data sources and associated measurements and records of interest.

Data Sources and Linking Method	Measurement(s) of Interest
National Minimum Dataset (NMDS) held by the NZ Ministry of Health, linked via NHI	Previous hospitalisations (dates, diagnostic codes) total and for infectious diseases (respiratory, skin)
Maternity Collections held by the NZ Ministry of Health, linked via NHI	Early life exposures, e.g., Low birth weight, pre-term delivery, Apgar score
Clinical data held by DHBs on ARF cases, linked via NHI	Clinical information for case review (clinical record, laboratory results, cardiac ultrasound) Height, weight and BMI
Dental records held by DHBs and other service providers, linked via name	Decayed, Missing, Filled Teeth (dmft/DMFT) scores obtained from dental service providers
Housing records from a national housing and valuation database, linked via address	Age and floor area of the house
Census data and NZDep, linked via home address	NZDep of home meshblock, Population and density of home meshblock
NZ Ministry of Education schools data, linked via school name	School size
Record of schools participating in RFPP held by NZ Ministry of Health, linked via school name	Attendance at a school that provided a school-based throat swabbing programme at the time of illness or interview
Controls	Date recruited and interviewed for NZHS Height, weight, BMI

## Supplementary Materials

The following are available online at https://www.mdpi.com/1660-4601/16/22/4515/s1, Table S1: Incidence of ARF hospitalisations by DHB, 2010–2014; Table S2: Summary of previous ARF and RHD risk factor studies; Table S3: Factors investigated in the ARF Risk Factors Study; Table S4: Potential risks to study effectiveness and their management; Figure S1: Case recruitment algorithm used by clinicians.

## References

[1] New Zealand Ministry of Health Progress on the Better Public Services Rheumatic Fever Target Wellington. http://www.health.govt.nz/about-ministry/what-we-do/strategic-direction/betterpublic-services/progress-better-public-services-rheumatic-fever-target.

[2] Carapetis J.R., Zuhlke L.J. (2011). Global research priorities in rheumatic fever and rheumatic heart disease. Ann. Pediatric Cardiol..

[3] Health Research Council of New Zealand (2013). Rheumatic Fever Research Partnership.

[4] Allison V.D., Gunn W. (1932). The Epidemiology of Streptococcal Infections. Proc. R. Soc. Med..

[5] Carapetis J.R., Beaton A., Cunningham M.W., Guilherme L., Karthikeyan G., Mayosi B.M., Sable C., Steer A., Wilson N., Wyber R. (2016). Acute rheumatic fever and rheumatic heart disease. Nat. Rev. Dis. Primers.

[6] Bright P.D., Mayosi B.M., Martin W.J. (2016). An immunological perspective on rheumatic heart disease pathogenesis: More questions than answers. Heart.

[7] Martin D.R., Voss L.M., Walker S.J., Lennon D. (1994). Acute rheumatic fever in Auckland, New Zealand: Spectrum of associated group A streptococci different from expected. Pediatric Infect. Dis. J..

[8] McDonald M., Currie B.J., Carapetis J.R. (2004). Acute rheumatic fever: A chink in the chain that links the heart to the throat?. Lancet Infect. Dis..

[9] Parks T., Smeesters P.R., Steer A.C. (2012). Streptococcal skin infection and rheumatic heart disease. Curr. Opin. Infect. Dis..

[10] Lennon D., Stewart J., Farrell E., Palmer A., Mason H. (2009). School-based prevention of acute rheumatic fever: A group randomized trial in New Zealand. Pediatric Infect. Dis. J..

[11] O’Sullivan L., Moreland L.J., Webb R.H., Upton A., Wilson N.J. (2017). Acute rheumatic fever following Group a Steptococcus pyoderma and Group G Streptococcus pharyngitis. Pediatric Infect. Dis. J..

[12] Kaplan M.H., Bolande R., Rakita L., Blair J. (1964). Presence of Bound Immunoglobulins and Complement in the Myocardium in Acute Rheumatic Fever. Association with Cardiac Failure. N. Engl. J. Med..

[13] Saphir O. (1959). The Aschoff nodule. Am. J. Clin. Pathol..

[14] Roberts S., Kosanke S., Terrence Dunn S., Jankelow D., Duran C.M., Cunningham M.W. (2001). Pathogenic mechanisms in rheumatic carditis: Focus on valvular endothelium. J. Infect. Dis..

[15] Lennon D.R., Cherry J., Demmler-Harrison G.J., Kaplan S.L., Steinbach W.J., Hotez P. (2013). Acute Rheumatic Fever. Feigin & Cherry’s Textbook of Pediatric Infectious Diseases.

[16] Spinetto H., Lennon D., Horsburgh M. (2011). Rheumatic fever recurrence prevention: A nurse-led programme of 28-day penicillin in an area of high endemnicity. J. Paediatr. Child Health.

[17] Bryant P.A., Robins-Browne R., Carapetis J.R., Curtis N., Bryant P.A., Robins-Browne R., Carapetis J.R., Curtis N. (2009). Some of the people, some of the time: Susceptibility to acute rheumatic fever. Circulation.

[18] Thornley C., McNicholas A., Baker M., Lennon D. (2001). Rheumatic fever registers in New Zealand. N. Z. Public Health Rep..

[19] Watkins D.A., Johnson C.O., Colquhoun S.M., Karthikeyan G., Beaton A., Bukhman G., Forouzanfar M.H., Longenecker C.T., Mayosi B.M., Mensah G.A. (2017). Global, Regional, and National Burden of Rheumatic Heart Disease, 1990–2015. N. Engl. J. Med..

[20] Steer A.C. (2015). Historical aspects of rheumatic fever. J. Paediatr. Child Health.

[21] Milne R.J., Lennon D.R., Stewart J.M., Vander Hoorn S., Scuffham P.A. (2012). Incidence of acute rheumatic fever in New Zealand children and youth. J. Paediatr. Child Health.

[22] Australian Institute of Health and Welfare (2013). Rheumatic Heart Disease and Acute Rheumatic Fever in Australia: 1996–2012. Cardiovascular Disease Series. Cat. No. CVD 60.

[23] Jaine R., Baker M., Venugopal K. (2008). Epidemiology of acute rheumatic fever in New Zealand 1996–2005. J. Paediatr. Child Health.

[24] Webb R., Wilson N. (2013). Rheumatic fever in New Zealand. J. Paediatr. Child Health.

[25] Oliver J., Pierse N., Baker M.G. (2015). Estimating rheumatic fever incidence in New Zealand using multiple data sources. Epidemiol. Infect..

[26] Gurney J.K., Stanley J., Baker M.G., Wilson N.J., Sarfati D. (2016). Estimating the risk of acute rheumatic fever in New Zealand by age, ethnicity and deprivation. Epidemiol. Infect..

[27] Siriett V., Crengle S., Lennon D., Stonehouse M., Cramp G. (2012). The epidemiology of rheumatic fever in the Tairawhiti/Gisborne region of New Zealand: 1997–2009. N. Z. Med. J..

[28] Robin A., Mills C., Tuck R., Lennon D. (2013). The epidemiology of acute rheumatic fever in Northland, 2002–2011. N. Z. Med. J..

[29] Pennock V., Bell A., Moxon T.A., Reed P., Maxwell F., Lennon D. (2014). Retrospective epidemiology of acute rheumatic fever: A 10-year review in the Waikato District Health Board area of New Zealand. N. Z. Med. J..

[30] Moxon T.A., Reed P., Jelleyman T., Anderson P., Leversha A., Jackson C., Lennon D. (2017). Is a rheumatic fever register the best surveillance tool to evaluate rheumatic fever control in the Auckland region?. N. Z. Med. J..

[31] Baker M.G., Telfar Barnard L., Kvalsvig A., Verrall A., Zhang J., Keall M., Wilson N., Wall T., Howden-Chapman P. (2012). Increasing incidence of serious infectious diseases and inequalities in New Zealand: A national epidemiological study. Lancet.

[32] New Zealand Ministry of Health (2017). HISO 10001:2017 Ethnicity Data Protocols.

[33] Atkinson J., Salmond C., Crampton P. (2014). NZDep2013 Index of Deprivation.

[34] Salmond C.E., Crampton P. (2012). Development of New Zealand’s deprivation index (NZDep) and its uptake as a national policy tool. Can. J. Public Health.

[35] Milne R.J., Lennon D., Stewart J.M., Vander Hoorn S., Scuffham P.A. (2012). Mortality and hospitalisation costs of rheumatic fever and rheumatic heart disease in New Zealand. J. Paediatr. Child Health.

[36] Oliver J., Pierse N., Baker M.G. (2014). Improving rheumatic fever surveillance in New Zealand: Results of a surveillance sector review. BMC Public Health.

[37] Kerdemelidis M., Lennon D.R., Arroll B., Peat B., Jarman J. (2010). The primary prevention of rheumatic fever. J. Paediatr. Child Health.

[38] Azevedo P.M., Pereira R.R., Guilherme L. (2012). Understanding rheumatic fever. Rheumatol. Int..

[39] Entine M. (1949). A survey of dental diseases as a diagnostic aid in rheumatic fever. J. Am. Dent. Assoc..

[40] Grave P.E. (1957). Social and environmental factors in the aetiology of rheumatic fever. Med. J. Aust..

[41] Coburn A.F. (1960). The concept of egg yolk as a dietary inhibitor to rheumatic susceptibility. Lancet.

[42] Adanja B., Vlajinac H., Jarebinski M. (1988). Socioeconomic factors in the etiology of rheumatic fever. J. Hyg. Epidemiol. Microbiol. Immunol..

[43] Vlajinac H., Adanja B., Jarebinski M. (1989). Socio-economic factors and rheumatic fever occurrence. Differences between patients with and without frequent sore throat. J. Hyg. Epidemiol. Microbiol. Immunol..

[44] Bahr G.M., Eales L.J., Nye K.E., Majeed H.A., Yousof A.M., Behbehani K., Rook G.A. (1989). An association between Gc (vitamin D-binding protein) alleles and susceptibility to rheumatic fever. Immunology.

[45] Vlajinac H., Adanja B., Marinkovic J., Jarebinski M. (1991). Influence of socio-economic and other factors on rheumatic fever occurrence. Eur. J. Epidemiol..

[46] Adanja B.J., Vlajinac H.D., Marinkovic J.P., Jarebinski M.S. (1991). Rheumatic fever and diet. Isr. J. Med. Sci..

[47] Thomas D.R., Salmon R.L., Kench S.M., Meadows D., Coleman T.J., Morgan-Capner P., Morgan K.L. (1994). Zoonotic illness—Determining risks and measuring effects: Association between current animal exposure and a history of illness in a well characterised rural population in the UK. J. Epidemiol. Community Health.

[48] Zaman M.M., Yoshiike N., Chowdhury A.H., Jalil M.Q., Mahmud R.S., Faruque G.M., Rouf M.A., Haque K.M., Tanaka H. (1997). Socio-economic deprivation associated with acute rheumatic fever. A hospital-based case-control study in Bangladesh. Paediatr. Perinat. Epidemiol..

[49] Zaman M.M., Yoshiike N., Chowdhury A.H., Nakayama T., Yokoyama T., Faruque G.M., Rouf M.A., Haque S., Tanaka H. (1998). Nutritional factors associated with rheumatic fever. J. Trop. Pediatric.

[50] Zaman M.M., Yoshiike N., Rouf M.A., Haque S., Chowdhury A.H., Nakayama T., Tanaka H. (1998). Association of rheumatic fever with serum albumin concentration and body iron stores in Bangladeshi children: Case-control study. BMJ.

[51] Berdeli A., Celik H.A., Ozyurek R., Aydin H.H. (2004). Involvement of immunoglobulin FcgammaRIIA and FcgammaRIIIB gene polymorphisms in susceptibility to rheumatic fever. Clin. Biochem..

[52] Berdeli A., Celik H.A., Ozyurek R., Dogrusoz B., Aydin H.H. (2005). TLR-2 gene Arg753Gln polymorphism is strongly associated with acute rheumatic fever in children. J. Mol. Med..

[53] Berdeli A., Tabel Y., Celik H.A., Ozyurek R., Dogrusoz B., Aydin H.H. (2006). Lack of association between TNF α gene polymorphism at position-308 and risk of acute rheumatic fever in Turkish patients. Scand. J. Rheumatol..

[54] Kurahara D.K., Grandinetti A., Galario J., Reddy D.V., Tokuda A., Langan S., Tanabe B., Yamamoto K.S., Yamaga K.M., Kurahara D.K. (2006). Ethnic differences for developing rheumatic fever in a low-income group living in Hawaii. Ethn. Dis..

[55] Hounie A.G., Pauls D.L., do Rosario-Campos M.C., Mercadante M.T., Diniz J.B., De Mathis M.A., De Mathis M.E., Chacon P., Shavitt R.G., Curi M. (2007). Obsessive-compulsive spectrum disorders and rheumatic fever: A family study. Biol. Psychiatry.

[56] Seixas A.A., Hounie A.G., Fossaluza V., Curi M., Alvarenga P.G., De Mathis M.A., De Mathis M.E., Vallada H., Pauls D., de Braganca Pereira C.A. (2008). Anxiety disorders and rheumatic Fever: Is there an association?. CNS Spectr..

[57] Messias-Reason I.J., Schafranski M.D., Kremsner P.G., Kun J.F. (2009). Ficolin 2 (FCN2) functional polymorphisms and the risk of rheumatic fever and rheumatic heart disease. Clin. Exp. Immunol..

[58] Walker K.G., Cooper M., McCabe K., Hughes J., Mathiassen W., Lawrenson J., Wilmshurst J.M. (2011). Markers of susceptibility to acute rheumatic fever: The B-cell antigen D8/17 is not robust as a marker in South Africa. Cardiol. Young.

[59] Col-Araz N., Pehlivan S., Baspinar O., Oguzkan-Balci S., Sever T., Balat A. (2012). Role of cytokine gene (IFN-gamma, TNF-alpha, TGF-beta1, IL-6, and IL-10) polymorphisms in pathogenesis of acute rheumatic fever in Turkish children. Eur. J. Pediatric.

[60] Riaz B.K., Selim S., Karim M.N., Chowdhury K.N., Chowdhury S.H., Rahman M.R. (2013). Risk factors of rheumatic heart disease in Bangladesh: A case-control study. J. Health Popul. Nutr..

[61] Thornley S., Marshall R.J., Bach K., Koopu P., Reynolds G., Sundborn G., Ei W.L. (2017). Sugar, dental caries and the incidence of acute rheumatic fever: A cohort study of Maori and Pacific children. J. Epidemiol. Community Health.

[62] Thornley S., Marshall R., Jarrett P., Sundborn G., Reynolds E., Schofield G. (2018). Scabies is strongly associated with acute rheumatic fever in a cohort study of Auckland children. J. Paediatr. Child Health.

[63] McLaren M.J., Hawkins D.M., Koornhof H.J., Bloom K.R., Bramwell-Jones D.M., Cohen E., Gale G.E., Kanarek K., Lachman A.S., Lakier J.B. (1975). Epidemiology of rheumatic heart disease in black shcoolchildren of Soweto, Johannesburg. BMJ.

[64] Caughey D.E., Douglas R., Wilson W., Hassall I.B. (1975). HL-A antigens in Europeans and Maoris with rheumatic fever and rheumatic heart disease. J. Rheumatol..

[65] Anabwani G.M., Amoa A.B., Muita A.K. (1989). Epidemiology of rheumatic heart disease among primary school children in western Kenya. Int. J. Cardiol..

[66] Coggon D., Barker D.J., Inskip H., Wield G. (1993). Housing in early life and later mortality. J. Epidemiol. Community Health.

[67] Longo-Mbenza B., Bayekula M., Ngiyulu R., Kintoki V.E., Bikangi N.F., Seghers K.V., Lukoki L.E., Mandundu M.F., Manzanza M., Nlandu Y. (1998). Survey of rheumatic heart disease in school children of Kinshasa town. Int. J. Cardiol..

[68] Oli K., Porteous J. (1999). Prevalence of rheumatic heart disease among school children in Addis Ababa. East Afr. Med. J..

[69] Rizvi S.F., Khan M.A., Kundi A., Marsh D.R., Samad A., Pasha O. (2004). Status of rheumatic heart disease in rural Pakistan. Heart.

[70] Chou H.T., Tsai C.H., Tsai F.J. (2004). Association between angiotensin I-converting enzyme gene insertion/deletion polymorphism and risk of rheumatic heart disease. Jpn. Heart J..

[71] Steer A.C., Kado J., Wilson N., Tuiketei T., Batzloff M., Waqatakirewa L., Mulholland E.K., Carapetis J.R. (2009). High prevalence of rheumatic heart disease by clinical and echocardiographic screening among children in Fiji. J. Heart Valve Dis..

[72] Azevedo P.M., Bauer R., Caparbo Vde F., Silva C.A., Bonfa E., Pereira R.M. (2010). Interleukin-1 receptor antagonist gene (IL1RN) polymorphism possibly associated to severity of rheumatic carditis in a Brazilian cohort. Cytokine.

[73] Saxena A., Ramakrishnan S., Roy A., Seth S., Krishnan A., Misra P., Kalaivani M., Bhargava B., Flather M.D., Poole-Wilson P.P. (2011). Prevalence and outcome of subclinical rheumatic heart disease in India: The RHEUMATIC (Rheumatic Heart Echo Utilisation and Monitoring Actuarial Trends in Indian Children) study. Heart.

[74] Ba-Saddik I.A., Munibari A.A., Al-Naqeeb M.S., Parry C.M., Hart C.A., Cuevas L.E., Coulter J.B.S. (2011). Prevalence of rheumatic heart disease among school-children in Aden, Yemen. Ann. Trop. Paediatr..

[75] Dobson J., Steer A.C., Colquhoun S., Kado J. (2012). Environmental factors and rheumatic heart disease in Fiji. Pediatric Cardiol..

[76] Okello E., Kakande B., Sebatta E., Kayima J., Kuteesa M., Mutatina B., Nyakoojo W., Lwabi P., Mondo C.K., Odoi-Adome R. (2012). Socioeconomic and environmental risk factors among rheumatic heart disease patients in Uganda. PLoS ONE.

[77] Eriksson J.G., Kajantie E., Phillips D.I., Osmond C., Thornburg K.L., Barker D.J. (2013). The developmental origins of chronic rheumatic heart disease. Am. J. Hum. Biol..

[78] Rehman S., Akhtar N., Saba N., Munir S., Ahmed W., Mohyuddin A., Khanum A. (2013). A study on the association of TNF-α (−308), IL-6 (−174), IL-10 (−1082) and IL-1Ra(VNTR) gene polymorphisms with rheumatic heart disease in Pakistani patients. Cytokine.

[79] Mirabel M., Fauchier T., Bacquelin R., Tafflet M., Germain A., Robillard C., Rouchon B., Marijon E., Jouven X. (2015). Echocardiography screening to detect rheumatic heart disease: A cohort study of schoolchildren in French Pacific Islands. Int. J. Cardiol..

[80] Azevedo P.M., Merriman T.R., Topless R.K., Wilson N.J., Crengle S., Lennon D.R. (2016). Association study involving polymorphisms in IL-6, IL-1RA, and CTLA4 genes and rheumatic heart disease in New Zealand population of Maori and Pacific ancestry. Cytokine.

[81] Gray L.A., D’Antoine H.A., Tong S.Y.C., McKinnon M., Bessarab D., Brown N., Remenyi B., Steer A., Syn G., Blackwell J.M. (2017). Genome-wide analysis of genetic risk factors for rheumatic heart disease in Aboriginal Australians provides support for pathogenic molecular mimicry. J. Infect. Dis..

[82] Parks T., Mirabel M.M., Kado J., Auckland K., Nowak J., Rautanen A., Mentzer A.J., Marijon E., Jouven X., Perman M.L. (2017). Association between a common immunoglobulin heavy chain allele and rheumatic heart disease risk in Oceania. Nat. Commun..

[83] Gordis L., Lilienfeld A., Rodriguez R. (1969). Studies in the epidemiology and preventability of rheumatic fever. II. Socio-economic factors and the incidence of acute attacks. J. Chronic Dis..

[84] Jaine R., Baker M., Venugopal K. (2011). Acute rheumatic fever associated with household crowding in a developed country. Pediatric Infect. Dis. J..

[85] Dajani A.S. (1991). Current status of nonsuppurative complications of group A streptococci. Pediatric Infect. Dis. J..

[86] Catanzaro F.J., Rammelkamp C.H., Chamovitz R. (1958). Prevention of rheumatic fever by treatment of streptococcal infections. II. Factors responsible for failures. N. Engl. J. Med..

[87] McDonald M.I., Towers R.J., Andrews R.M., Benger N., Currie B.J., Carapetis J.R. (2006). Low rates of streptococcal pharyngitis and high rates of pyoderma in Australian aboriginal communities where acute rheumatic fever is hyperendemic. Clin. Infect. Dis..

[88] Bowen A.C., Mahe A., Hay R.J., Andrews R.M., Steer A.C., Tong S.Y., Carapetis J.R. (2015). The Global Epidemiology of Impetigo: A Systematic Review of the Population Prevalence of Impetigo and Pyoderma. PLoS ONE.

[89] Williamson D.A., Smeesters P.R., Steer A.C., Steemson J.D., Ng A.C., Proft T., Fraser J.D., Baker M.G., Morgan J., Carter P.E. (2015). M-Protein Analysis of Streptococcus pyogenes Isolates Associated with Acute Rheumatic Fever in New Zealand. J. Clin. Microbiol..

[90] O’Sullivan C.E., Baker M.G., Zhang J. (2011). Increasing hospitalizations for serious skin infections in New Zealand children, 1990–2007. Epidemiol. Infect..

[91] Tupai-Firestone R., Tsai J.Y., Anderson P., Broome L., McKee T., Lennon D.R. (2016). Antimicrobial stewardship using pharmacy data for the nurse-led school-based clinics in Counties Manukau District Health Board for management of group A streptococcal pharyngitis and skin infection. N. Z. Med. J..

[92] Anderson P., King J., Moss M., Light P., McKee T., Farrell E., Stewart J., Lennon D. (2016). Nurse-led school-based clinics for rheumatic fever prevention and skin infection management: Evaluation of Mana Kidz programme in Counties Manukau. N. Z. Med. J..

[93] O’Sullivan C.E., Baker M.G. (2010). Proposed epidemiological case definition for serious skin infection in children. J. Paediatr. Child Health.

[94] Romani L., Steer A.C., Whitfeld M.J., Kaldor J.M. (2015). Prevalence of scabies and impetigo worldwide: A systematic review. Lancet Infect. Dis..

[95] Fischer K., Holt D., Currie B., Kemp D. (2012). Scabies: Important clinical consequences explained by new molecular studies. Adv. Parasitol..

[96] Richardson M., Elliman D., Maguire H., Simpson J., Nicoll A. (2001). Evidence base of incubation periods, periods of infectiousness and exclusion policies for the control of communicable diseases in schools and preschools. Pediatric Infect. Dis. J..

[97] Danchin M.H., Rogers S., Kelpie L., Selvaraj G., Curtis N., Carlin J.B., Nolan T.M., Carapetis J.R. (2007). Burden of acute sore throat and group A streptococcal pharyngitis in school-aged children and their families in Australia. Pediatrics.

[98] Zimmer C. (2001). Evolution: The Triumph of an Idea.

[99] Stolleman G.H. (1961). Factors determining the attack rate of rheumatic fever. JAMA.

[100] Schneider W.F., Chapman S., Schulz V.B., Krause R.M., Lancefield R.C. (1964). Prevention of streptococcal pharyngitis among military personnel and their civilian dependents by mass prophylaxis. N. Engl. J. Med..

[101] Gray G.C., Callahan J.D., Hawksworth A.W., Fisher C.A., Gaydos J.C. (1999). Respiratory diseases among US military personnel: Countering emerging threats. Emerg. Infect. Dis..

[102] Baker M., McNicholas A., Garrett N., Jones N., Stewart J., Koberstein V., Lennon D. (2000). Household crowding a major risk factor for epidemic meningococcal disease in Auckland children. Pediatric Infect. Dis. J..

[103] Grant C.C., Emery D., Milne T., Coster G., Forrest C.B., Wall C.R., Scragg R., Aickin R., Crengle S., Leversha A. (2012). Risk factors for community-acquired pneumonia in pre-school-aged children. J. Paediatr. Child Health.

[104] Baker M., Das D., Venugopal K., Howden-Chapman P. (2008). Tuberculosis associated with household crowding in a developed country. J. Epidemiol. Community Health.

[105] Ransome O.J., Roode H., Spector I., Reinach S.G. (1983). Pharyngeal carriage of group A beta-haemolytic streptococci in coloured and Indian schoolchildren. S. Afr. Med. J..

[106] Nandi S., Kumar R., Ray P., Vohra H., Ganguly N.K. (2001). Group A streptococcal sore throat in a periurban population of northern India: A one-year prospective study. Bull. World Health Organ..

[107] Tay L., Chay S.O. (1981). A three-year streptococcal survey among Singapore school children. Part I—Carriership of streptococci. Ann. Acad. Med. Singap..

[108] Faruq Q.O., Rashid A.K., Ahmed J., Waiz A., Haque K.M., Rouf M.A., Khan S.M., Khan T.N. (1995). Prevalence of streptococcal sorethroat in the school children of Dhaka. Bangladesh Med. Res. Counc. Bull..

[109] Likitnukul S., Prapphal N., Tatiyakavee K., Nunthapisud P., Chumdermpadetsuk S. (1994). Risk factors of streptococcal colonization in school age children. Southeast Asian J. Trop. Med. Public Health.

[110] Spitzer J., Hennessy E., Neville L. (2001). High group A streptococcal carriage in the Orthodox Jewish community of north Hackney. Br. J. Gen. Pract..

[111] McDonald M.I., Towers R.J., Andrews R., Benger N., Fagan P., Currie B.J., Carapetis J.R. (2008). The dynamic nature of group A streptococcal epidemiology in tropical communities with high rates of rheumatic heart disease. Epidemiol. Infect..

[112] Oliver J.R., Pierse N., Stefanogiannis N., Jackson C., Baker M.G. (2017). Acute rheumatic fever and exposure to poor housing conditions in New Zealand: A descriptive study. J. Paediatr. Child Health.

[113] Kramer A., Schwebke I., Kampf G. (2006). How long do nosocomial pathogens persist on inanimate surfaces? A systematic review. BMC Infect. Dis..

[114] Bygdeman S., Jacobsson E., Myrback K.E., Wallmark G. (1978). Hemolytic streptococci among infants in a maternity department. Report of an outbreak. Scand. J. Infect. Dis..

[115] Claesson B.E., Claesson U.L. (1985). An outbreak of endometritis in a maternity unit caused by spread of group A streptococci from a showerhead. J. Hosp. Infect..

[116] Wagenvoort J.H., Penders R.J., Davies B.I., Lutticken R. (2005). Similar environmental survival patterns of Streptococcus pyogenes strains of different epidemiologic backgrounds and clinical severity. Eur. J. Clin. Microbiol. Infect. Dis..

[117] Luby S.P., Agboatwalla M., Feikin D.R., Painter J., Billhimer W., Altaf A., Hoekstra R.M. (2005). Effect of handwashing on child health: A randomised controlled trial. Lancet.

[118] Carapetis J.R., Johnston F., Nadjamerrek J., Kairupan J. (1995). Skin sores in Aboriginal children. J. Paediatr. Child Health.

[119] Buckett N.R., Marston N.J., Saville-Smith K., Jowett J.H., Jones M.S. (2011). Preliminary BRANZ 2010 House Condition Survey Report.

[120] Rushdy A.A., Cooke R.P., Iversen A.M., Pickering B.J. (1995). Boarding school outbreak of group A streptococcal pharyngitis. Commun. Dis. Rep. CDR Rev..

[121] Shorter C., Crane J., Pierse N., Barnes P., Kang J., Wickens K., Douwes J., Stanley T., Taubel M., Hyvarinen A. (2018). Indoor visible mold and mold odor are associated with new-onset childhood wheeze in a dose-dependent manner. Indoor Air.

[122] Pierse N., Arnold R., Keall M., Howden-Chapman P., Crane J., Cunningham M., Heating H., Health Study Group (2013). Modelling the effects of low indoor temperatures on the lung function of children with asthma. J. Epidemiol. Community Health.

[123] Craig E., Anderson P., Jackson G., Jackson C. (2012). Measuring potentially avoidable and ambulatory care sensitive hospitalisations in New Zealand children using a newly developed tool. N. Z. Med. J..

[124] Vanker A., Gie R.P., Zar H.J. (2017). The association between environmental tobacco smoke exposure and childhood respiratory disease: A review. Expert Rev. Respir. Med..

[125] Brook I. (2011). The impact of smoking on oral and nasopharyngeal bacterial flora. J. Dent. Res..

[126] Greene C.E. (2013). Infectious Diseases of the Dog and Cat.

[127] Biberstein E.L., Brown C., Smith T. (1980). Serogroups and biotypes among beta-hemolytic streptococci of canine origin. J. Clin. Microbiol..

[128] Pichichero M.E., Casey J.R. (2007). Systematic review of factors contributing to penicillin treatment failure in Streptococcus pyogenes pharyngitis. JAMA Otolaryngol. Head Neck Surg..

[129] Falck G. (1997). Group A streptococci in household pets’ eyes—A source of infection in humans?. Scand. J. Infect. Dis..

[130] Wilson K.S., Maroney S.A., Gander R.M. (1995). The family pet as an unlikely source of group A beta-hemolytic streptococcal infection in humans. Pediatric Infect. Dis. J..

[131] Arya R.K. (1992). Awareness about sore-throat, rheumatic fever and rheumatic heart disease in a rural community. Indian J. Public Health.

[132] Iyengar S.D., Grover A., Kumar R., Ganguly N.K., Wahi P.L. (1992). Participation of health workers, school teachers and pupils in the control of rheumatic fever: Evaluation of a training programme. Indian Pediatric.

[133] Harre N., Thomas D., Brown K., Raza F., Lennon D. (2000). Communicating information about sore throats and rheumatic fever to South Auckland high-school students. N. Z. Med. J..

[134] World Health Organization Technical Report Series (2004). Rheumatic Fever and Rheumatic Heart Disease.

[135] Robertson K.A., Volmink J.A., Mayosi B.M. (2006). Towards a uniform plan for the control of rheumatic fever and rheumatic heart disease in Africa—The Awareness Surveillance Advocacy Prevention (A.S.A.P.) Programme. S. Afr. Med. J..

[136] Allen L.B., Allen M., Lesa R.F., Richardson G.E., Eggett D.L. (2011). Rheumatic fever in Samoa: Education as prevention. Pac. Health Dialog.

[137] Jack S.J., Williamson D.A., Galloway Y., Pierse N., Zhang J., Oliver J., Milne R.J., Mackereth G., Jackson C.M., Steer A.C. (2018). Primary prevention of rheumatic fever in the 21st century: Evaluation of a national programme. Int. J. Epidemiol..

[138] Nordet P., Lopez R., Duenas A., Sarmiento L. (2008). Prevention and control of rheumatic fever and rheumatic heart disease: The Cuban experience (1986–1996–2002). Cardiovasc. J. Afr..

[139] Bach J.F., Chalons S., Forier E., Elana G., Jouanelle J., Kayemba S., Delbois D., Mosser A., Saint-Aime C., Berchel C. (1996). 10-year educational programme aimed at rheumatic fever in two French Caribbean islands. Lancet.

[140] Ramsey L.S., Watkins L., Engel M.E. (2013). Health education interventions to raise awareness of rheumatic fever: A systematic review protocol. Syst. Rev..

[141] Chang C. (2012). Cutting edge issues in rheumatic fever. Clin. Rev. Allergy Immunol..

[142] Andrulis D.P. (1998). Access to care is the centerpiece in the elimination of socioeconomic disparities in health. Ann. Intern. Med..

[143] Gordis L. (1973). Effectiveness of comprehensive-care programs in preventing rheumatic fever. N. Engl. J. Med..

[144] Arguedas A., Mohs E. (1992). Prevention of rheumatic fever in Costa Rica. J. Pediatric.

[145] Eltohami E.A., Hajar H.A., Folger G.M. (1997). Acute rheumatic fever in an Arabian Gulf country—Effect of climate, advantageous socioeconomic conditions, and access to medical care. Angiology.

[146] Jatrana S., Crampton P. (2009). Primary health care in New Zealand: Who has access?. Health Policy.

[147] Robertson K.A., Volmink J.A., Mayosi B.M. (2005). Antibiotics for the primary prevention of acute rheumatic fever: A meta-analysis. BMC Cardiovasc. Disord..

[148] Spinks A., Glasziou P.P., Del Mar C.B. (2013). Antibiotics for sore throat. Cochrane Database Syst. Rev..

[149] Lennon D., Anderson P., Kerdemelidis M., Farrell E., Mahi S.C., Percival T., Jansen D., Stewart J. (2017). First Presentation Acute Rheumatic Fever is Preventable in a Community Setting: A School Based Intervention. Pediatric Infect. Dis. J..

[150] el-Daher N.T., Hijazi S.S., Rawashdeh N.M., al-Khalil I.A., Abu-Ektaish F.M., Abdel-Latif D.I. (1991). Immediate vs. delayed treatment of group A beta-hemolytic streptococcal pharyngitis with penicillin V. Pediatric Infect. Dis. J..

[151] Pichichero M.E., Disney F.A., Talpey W.B., Green J.L., Francis A.B., Roghmann K.J., Hoekelman R.A. (1987). Adverse and beneficial effects of immediate treatment of Group A beta-hemolytic streptococcal pharyngitis with penicillin. Pediatric Infect. Dis. J..

[152] Gerber M.A., Randolph M.F., DeMeo K.K., Kaplan E.L. (1990). Lack of impact of early antibiotic therapy for streptococcal pharyngitis on recurrence rates. J. Pediatric.

[153] New Zealand Ministry of Health Progress on the Better Public Services Rheumatic Fever Target. http://www.health.govt.nz/about-ministry/what-we-do/strategic-direction/better-public-services/progress-better-public-services-rheumatic-fever-target.

[154] Currie B.J., McCarthy J.S. (2010). Permethrin and ivermectin for scabies. N. Engl. J. Med..

[155] Tasani M., Tong S.Y., Andrews R.M., Holt D.C., Currie B.J., Carapetis J.R., Bowen A.C. (2016). The Importance of Scabies Coinfection in the Treatment Considerations for Impetigo. Pediatric Infect. Dis. J..

[156] Yeoh D.K., Bowen A.C., Carapetis J.R. (2016). Impetigo and scabies—Disease burden and modern treatment strategies. J. Infect..

[157] Price W.A. (2003). Nutrition and Physical Degeneration: A Comparison of Primitive and Modern Diets and Their Effects.

[158] Burton J.P., Drummond B.K., Chilcott C.N., Tagg J.R., Thomson W.M., Hale J.D.F., Wescombe P. (2013). Influence of the probiotic Streptococcus salivarius strain M18 on indices of dental health in children: A randomized double-blind, placebo-controlled trial. J. Med. Microbiol..

[159] Strom B.L., Abrutyn E., Berlin J.A., Kinman J.L., Feldman R.S., Stolley P.D., Levison M.E., Korzeniowski O.M., Kaye D. (2000). Risk factors for infective endocarditis: Oral hygiene and nondental exposures. Circulation.

[160] He J., Li Y., Cao Y., Xue J., Zhou X. (2015). The oral microbiome diversity and its relation to human diseases. Folia Microbiol..

[161] Saiman L., Prince A., Gersony W.M. (1993). Pediatric infective endocarditis in the modern era. J. Pediatric.

[162] Webb R., Voss L., Roberts S., Hornung T., Rumball E., Lennon D. (2014). Infective endocarditis in New Zealand children 1994–2012. Pediatric Infect. Dis. J..

[163] Thornley S., Sundborn G., Schmidt-Uili S.M. (2014). Rheumatic fever in New Zealand: What are the teeth trying to tell us?. Pac. Health Dialog.

[164] Chassy B.M., Beall J.R., Bielawski R.M., Porter E.V., Donkersloot J.A. (1976). Occurrence and distribution of sucrose-metabolizing enzymes in oral streptococci. Infect. Immun..

[165] Shelburne S.A., Keith D., Horstmann N., Sumby P., Davenport M.T., Graviss E.A., Brennan R.G., Musser J.M. (2008). A direct link between carbohydrate utilization and virulence in the major human pathogen group A Streptococcus. Proc. Natl. Acad. Sci. USA.

[166] Congiu G., Campus G., Luglie P.F. (2014). Early Childhood Caries (ECC) Prevalence and Background Factors: A Review. Oral Health Prev. Dent..

[167] Rafael da Silveira Moreira (2012). Epidemiology of Dental Caries in the World, Oral Health Care—Pediatric, Research, Epidemiology and Clinical Practices.

[168] Carapetis J.R., Steer A.C., Mulholland E.K., Weber M. (2005). The global burden of group A streptococcal diseases. Lancet Infect. Dis..

[169] Hewison M. (2012). Vitamin D and immune function: An overview. Proc. Nutr. Soc..

[170] Nseir W., Mograbi J., Abu-Rahmeh Z., Mahamid M., Abu-Elheja O., Shalata A. (2012). The association between vitamin D levels and recurrent group A streptococcal tonsillopharyngitis in adults. Int. J. Infect. Dis..

[171] Reid D., Morton R., Salkeld L., Bartley J. (2011). Vitamin D and tonsil disease--preliminary observations. Int. J. Pediatric Otorhinolaryngol..

[172] Utter J., Denny S., Teevale T., Peiris-John R., Dyson B. (2015). Prevalence and Recent Trends in Overweight, Obesity, and Severe Obesity among New Zealand Adolescents. Child Obes..

[173] Rajput N., Tuohy P., Mishra S., Smith A., Taylor B. (2015). Overweight and obesity in 4-5-year-old children in New Zealand: Results from the first 4 years (2009–2012) of the B4School Check programme. J. Paediatr. Child Health.

[174] Salmond C., Crampton P., King P., Waldegrave C. (2006). NZiDep: A New Zealand index of socioeconomic deprivation for individuals. Soc. Sci. Med..

[175] Vendsborg P., Hansen L.F., Olesen K.H. (1968). Decreasing incidence of a history of acute rheumatic fever in chronic rheumatic heart disease. Cardiologia.

[176] Gordis L. (1985). The virtual disappearance of rheumatic fever in the United States: Lessons in the rise and fall of disease. T. Duckett Jones memorial lecture. Circulation.

[177] Carapetis J.R. (2008). Rheumatic heart disease in Asia. Circulation.

[178] Omurzakova N.A., Yamano Y., Saatova G.M., Mirzakhanova M.I., Shukurova S.M., Kydyralieva R.B., Jumagulova A.S., Seisenbaev A., Nishioka K., Nakajima T. (2009). High incidence of rheumatic fever and rheumatic heart disease in the republics of Central Asia. Int. J. Rheum. Dis..

[179] White H., Walsh W., Brown A., Riddell T., Tonkin A., Jeremy R., Brieger D., Zeitz C., Kritharides L. (2010). Rheumatic heart disease in indigenous populations. Heart Lung Circ..

[180] Steer A.C., Carapetis J.R., Nolan T.M., Shann F. (2002). Systematic review of rheumatic heart disease prevalence in children in developing countries: The role of environmental factors. J. Paediatr. Child Health.

[181] Stollerman G.H. (1975). Rheumatic Fever and Streptococcal Infection.

[182] Coburn A. (1936). Observations on the mechanism of rheumatic fever. Lancet.

[183] Engel M.E., Stander R., Vogel J., Adeyemo A.A., Mayosi B.M. (2011). Genetic susceptibility to acute rheumatic fever: A systematic review and meta-analysis of twin studies. PLoS ONE.

[184] Stanhope J.M. (1975). New Zealand trends in rheumatic fever: 1885–1971. N. Z. Med. J..

[185] Nepom G.T., Erlich H. (1991). MHC class-II molecules and autoimmunity. Annu. Rev. Immunol..

[186] Padyukov L., Silva C., Stolt P., Alfredsson L., Klareskog L. (2004). A gene-environment interaction between smoking and shared epitope genes in HLA-DR provides a high risk of seropositive rheumatoid arthritis. Arthritis Rheum..

[187] Too C.L., Yahya A., Murad S., Dhaliwal J.S., Larsson P.T., Muhamad N.A., Abdullah N.A., Mustafa A.N., Klareskog L., Alfredsson L. (2012). Smoking interacts with HLA-DRB1 shared epitope in the development of anti-citrullinated protein antibody-positive rheumatoid arthritis: Results from the Malaysian Epidemiological Investigation of Rheumatoid Arthritis (MyEIRA). Arthritis Res. Ther..

[188] Boechat Nde O., Ogusku M.M., Boechat A.L., Sadahiro A. (2012). Interaction between smoking and HLA-DRB1*04 gene is associated with a high cardiovascular risk in Brazilian Amazon patients with rheumatoid arthritis. PLoS ONE.

[189] Fisher B.A., Bang S.Y., Chowdhury M., Lee H.S., Kim J.H., Charles P., Venables P., Bae S.C. (2013). Smoking, the HLA-DRB1 shared epitope and ACPA fine-specificity in Koreans with rheumatoid arthritis: Evidence for more than one pathogenic pathway linking smoking to disease. Ann. Rheum. Dis..

[190] Bang S.Y., Lee H.S., Lee K.W., Bae S.C. (2013). Interaction of HLA-DRB1 *09:01 and *04:05 with Smoking Suggests Distinctive Mechanisms of Rheumatoid Arthritis Susceptibility Beyond the Shared Epitope. J. Rheumatol..

[191] Sorensen H.T., Labouriau R., Jensen E.S., Mortensen P.B., Schonheyder H.C. (2004). Fetal growth, maternal prenatal smoking, and risk of invasive meningococcal disease: A nationwide case-control study. Int. J. Epidemiol..

[192] Melville J.M., Moss T.J. (2013). The immune consequences of preterm birth. Front. Neurosci..

[193] Yuan W., Basso O., Sorensen H.T., Olsen J. (2001). Indicators of fetal growth and infectious disease in childhood—A birth cohort with hospitalization as outcome. Eur. J. Epidemiol..

[194] Selling K.E., Carstensen J., Finnstrom O., Josefsson A., Sydsjo G. (2008). Hospitalizations in adolescence and early adulthood among Swedish men and women born preterm or small for gestational age. Epidemiology.

[195] Mahon B.E., Ehrenstein V., Norgaard M., Pedersen L., Rothman K.J., Sorensen H.T. (2007). Perinatal risk factors for hospitalization for pneumococcal disease in childhood: A population-based cohort study. Pediatrics.

[196] Goldacre M.J., Wotton C.J., Maisonneuve J.J. (2014). Maternal and perinatal factors associated with subsequent meningococcal, Haemophilus or enteroviral meningitis in children: Database study. Epidemiol. Infect..

[197] Cunningham M.W. (2014). Rheumatic fever revisited. Nat. Rev. Cardiol..

[198] Tandon R., Sharma M., Chandrashekhar Y., Kotb M., Yacoub M.H., Narula J. (2013). Revisiting the pathogenesis of rheumatic fever and carditis. Nat. Rev. Cardiol..

[199] Martin W.J., Steer A.C., Smeesters P.R., Keeble J., Inouye M., Carapetis J., Wicks I.P. (2015). Post-infectious group A streptococcal autoimmune syndromes and the heart. Autoimmun. Rev..

[200] Raynes J.M., Frost H.R., Williamson D.A., Young P.G., Baker E.N., Steemson J.D., Loh J.M., Proft T., Dunbar P.R., Atatoa Carr P.E. (2016). Serological Evidence of Immune Priming by Group A Streptococci in Patients with Acute Rheumatic Fever. Front. Microbiol..

[201] Gewitz M.H., Baltimore R.S., Tani L.Y., Sable C.A., Shulman S.T., Carapetis J., Remenyi B., Taubert K.A., Bolger A.F., Beerman L. (2015). Revision of the Jones Criteria for the diagnosis of acute rheumatic fever in the era of Doppler echocardiography: A scientific statement from the American Heart Association. Circulation.

[202] Shulman S.T., Stollerman G., Beall B., Dale J.B., Tanz R.R. (2006). Temporal changes in streptococcal M protein types and the near-disappearance of acute rheumatic fever in the United States. Clin. Infect. Dis..

[203] Shulman S., Bisno A., Bennett J.E., Dolin R., Blaser M.J. (2015). Non Suppurative Post Streptococcal Sequelae. Mandell, Douglas, and Bennett’s Principles and Practice of Infectious Diseases.

[204] Johnson D.R., Stevens D.L., Kaplan E.L. (1992). Epidemiologic analysis of group A streptococcal serotypes associated with severe systemic infections, rheumatic fever, or uncomplicated pharyngitis. J. Infect. Dis..

[205] Carapetis J.R., McDonald M., Wilson N.J. (2005). Acute rheumatic fever. Lancet.

[206] Erdem G., Mizumoto C., Esaki D., Reddy V., Kurahara D., Yamaga K., Abe L., Johnson D., Yamamoto K., Kaplan E.L. (2007). Group A streptococcal isolates temporally associated with acute rheumatic fever in Hawaii: Differences from the continental United States. Clin. Infect. Dis..

[207] Pruksakorn S., Sittisombut N., Phornphutkul C., Pruksachatkunakorn C., Good M.F., Brandt E. (2000). Epidemiological analysis of non-M-typeable group A Streptococcus isolates from a Thai population in northern Thailand. J. Clin. Microbiol..

[208] Williamson D.A., Smeesters P.R., Steer A.C., Morgan J., Davies M., Carter P., Upton A., Tong S.Y., Fraser J., Moreland N.J. (2016). Comparative M-protein analysis of Streptococcus pyogenes from pharyngitis and skin infections in New Zealand: Implications for vaccine development. BMC Infect. Dis..

[209] Williamson D.A., Ritchie S.R., Lennon D., Roberts S.A., Stewart J., Thomas M.G., Baker M.G. (2013). Increasing Incidence and Sociodemographic Variation in Community-Onset Staphylococcus Aureus Skin and Soft Tissue Infections in New Zealand Children. Pediatric Infect. Dis. J..

[210] Olgunturk R., Okur I., Cirak M.Y., Oguz A.D., Akalin N., Turet S., Tunaoglu S. (2011). The role of viral agents in aetiopathogenesis of acute rheumatic fever. Clin. Rheumatol..

[211] Doyle H., Pierse N., Tiatia R., Williamson D., Baker M., Crane J. (2017). The effect of the oral probiotic Streptococcus salivarius (K12) on Group A Streptococcus pharyngitis: A pragmatic trial in schools. Pediatric Infect. Dis. J..

[212] Heart Foundation of New Zealand (2014). New Zealand Guidelines for Rheumatic Fever: Diagnosis, Management and Secondary Prevention of Acute Rheumatic Fever and Rheumatic Heart Disease: 2014 Update.

[213] Jack S., Moreland N.J., Meagher J., Fittock M., Galloway Y., Ralph A.P. (2018). Streptococcal Serology in Acute Rheumatic Fever Patients: Findings from Two High-income, High Burden Settings. Pediatric Infect. Dis. J..

[214] Burke R.J., Chang C. (2014). Diagnostic criteria of acute rheumatic fever. Autoimmun. Rev..

[215] Dajani A.S., Ayoub E., Bierman F.Z. (1992). Guidelines for the diagnosis of rheumatic fever: Jones criteria, 1992 update. JAMA.

[216] Wilson N.J., Voss L., Morreau J., Stewart J., Lennon D. (2013). New Zealand guidelines for the diagnosis of acute rheumatic fever: Small increase in the incidence of definite cases compared to the American Heart Association Jones criteria. N. Z. Med. J..

[217] New Zealand Ministry of Health (2016). Methodology Report 2015/16: New Zealand Health Survey.

[218] New Zealand Ministry of Health (2012). New Zealand Health Survey Methodology Report.

[219] Huang Q.S., Turner N., Baker M.G., Williamson D.A., Wong C., Webby R., Widdowson M.A. (2015). Southern Hemisphere Influenza and Vaccine Effectiveness Research and Surveillance. Influenza Respir. Viruses.

[220] Adolescent Health Research Group Youth’12 National Health and Wellbeing Survey of New Zealand Secondary School Students: Questionnaire. https://www.fmhs.auckland.ac.nz/assets/fmhs/faculty/ahrg/docs/youth12-questionnaire.pdf.

[221] Kelly A., Denning-Kemp G., Geiringer K., Abdulhamid A., Albabtain A., Beard M., Brimble J., Campbell A., Feng S., Haminudin M. (2013). Exposure to harmful housing conditions is common in children admitted to Wellington Hospital. N. Z. Med. J..

[222] Athey K.R., Coleman J.E., Reitman A.P., Tang J. (1960). Two experiments showing the effect of the interviewer’s racial background on responses to questionnaires concerning racial issues. J. Appl. Psychol..

[223] Greenland S., Daniel R., Pearce N. (2016). Outcome modelling strategies in epidemiology: Traditional methods and basic alternatives. Int. J. Epidemiol..

[224] Rockhill B., Newman B., Weinberg C. (1998). Use and misuse of population attributable fractions. Am. J. Public Health.

