# Supplementary material for: Risk Factors for Acute Rheumatic Fever: Literature Review and Protocol for a Case-Control Study in New Zealand
Source: Int J Environ Res Public Health. 2019 Nov 15;16(22):4515. doi: 10.3390/ijerph16224515 (PMC6888501; doi:10.3390/ijerph16224515)
Supplement: Supplementary file 1 [file ijerph-16-04515-s001.pdf]

Risk factors for acute rheumatic fever:
Protocol for a case-control study in New Zealand
Supplementary material

Michael G Baker*, Jason Gurney, Jane Oliver, Nikki Moreland, Deborah Williamson, Nevil Pierse, Nigel Wilson, Tony R Merriman, Teuila Percival, Colleen Murray, Catherine Jackson, Richard Edwards, Lyndie Foster Page, Florina Chan Mow, Angela Chong, Barry Gribben, Diana Lennon

*Corresponding author: Department of Public Health, University of Otago, Wellington

Box 7343 Wellington, New Zealand 6242

Email: michael.baker@otago.ac.nz

Contents of supplement:

Supplementary Table S1: Incidence of ARF hospitalisations (numbers and annual rates per 100,000) by District Health Board, 2010–2014

Supplementary Table S2: Summary of previous ARF and RHD risk factor studies

Supplementary Table S3: Factors investigated in the ARF Risk Factors Study

Supplementary Table S4: Potential risks to study effectiveness and their management

Supplementary Figure S1: Case recruitment algorithm used by clinicians

Supplementary Table S1: Incidence of ARF hospitalisations (numbers and annual rates per 100,000) by District Health Board, 2010–2014

District Health Board	Populat-ion 2013	Populat-ion <20 years	ARF hospital-isations, total no. 2010-14	First ARF hospital-isations, total no. (deduct readmiss ions)	First ARF hospital-isations, <20 years old, total no.	First ARF hospital-isations <20 years, annual average no.	First ARF hospital-isations <20 years % of NZ total	First ARF hospital-isations <20 years, annual average rate (/100,000 children)
Northland*	151692	42378	90	63	47	9.4	8.9	**22.2**
Waitemata*	525555	144942	80	53	42	8.4	8.0	**5.8**
Auckland*	436341	109014	80	65	47	9.4	8.9	**8.6**
Counties Manukau*	469293	150099	377	282	207	41.4	39.2	**27.6**
Waikato*	359310	103344	78	63	50	10	9.5	**9.7**
Lakes*	98187	28773	37	27	16	3.2	3.0	**11.1**
Bay of Plenty*	205995	56508	49	35	25	5	4.7	**8.8**
Tairawhiti*	43653	13782	15	14	10	2	1.9	**14.5**
Hawkes Bay*	151692	43116	24	20	13	2.6	2.5	**6.0**
Taranaki	109752	30015	6	5	2	0.4	0.4	**1.3**
Whanganui	60120	16407	8	6	4	0.8	0.8	**4.9**
Midcentral	162564	44799	12	9	8	1.6	1.5	**3.6**
Wairarapa	41112	10752	2	2	1	0.2	0.2	**1.9**
Hutt Valley*	138378	38517	38	33	23	4.6	4.4	**11.9**
Capital and Coast*	283704	73626	32	29	19	3.8	3.6	**5.2**
**North Island Total**	**3237348**	**906072**	**928**	**706**	**514**	**102.8**	**97.3**	**11.3**
Nelson Marlborough	136995	33891	5	5	3	0.6	0.6	**1.8**
West Coast	32148	7974	2	2	1	0.2	0.2	**2.5**
Canterbury	482178	122784	21	17	8	1.6	1.5	**1.3**
South Canterbury	55626	13506	0	0	0	0	0	**0.0**
Southern	297423	77133	6	6	0	0	0	**0.0**
**South Island Total**	**1004694**	**255324**	**34**	**30**	**12**	**2.4**	**2.3**	**0.9**
Unknown	**-**	**-**	**2**	**2**	**2**	0.4	0.4	**-**
**NZ total**	**4242048**	**1161387**	**964**	**738**	**528**	**105.6**	**100**	**9.1**

* RFRF case-control study = 11 DHBs in North Island

Supplementary Table S2: Summary of previous ARF and RHD risk factor studies

This structured review used the following search strategy:

- Databases searched: Medline/Pubmed, Embase plus manual search of references
- Outcomes: Acute rheumatic fever, Rheumatic heart disease
- Study design: cohort, case-control, cross-sectional
- Exclusion: Ecological studies, Non-English language, No comparison group, No statistical analysis of risk factors

Author, year, ref	Design	Outcome	Place	Population	Age group	N	Risk factors or interventions	Effect : OR (CI), RR (CI) HR (CI), aHR (CI)
**ARF**								
Entine 1949 [1]	Case-control	ARF incident case	USA	Cases: Children diagnosed with ARF attending outpatient and ARF clinics,<br>Controls: healthy children attending a periodontics appointment at Temple University School of Dentistry.	4-17yo	Cases: 100<br>Controls: 100	**Univariate:**<br>Non-suppurative gingivitis<br>Soft white tooth deposit/decalcification<br>Discoloration (orange-brown)<br>Average number of Carries/Restorations/ Extractions (CRE) were higher in ARF cases (13.2) compared with controls (8.1)	 OR 11.82 (4.0-34.93)<br>OR 2.75 (1.42-5.33)<br>OR 9.60 (3.83-24.07)<br>Statistical testing not reported
Grave 1957 [2]	Case-control	ARF incident case	Australia	Sydney children: Cases: diagnosed with ARF, Controls: out-patients with non-rheumatic conditions.	2-12 years	120 cases, 100 controls	**Univariate:**<br>Breadwinner is unskilled labourer/ receiving social service benefit<br>Substandard housing<br>Sleeping space <300 cubic feet per person<br>Damp house<br>Poor sewage/drainage<br>Low family income	 "Stat. significant"<br>"Stat. significant"<br>"Stat. significant"<br>"Stat. significant"<br>"Stat. significant"<br>"Stat. significant"

Author, year, ref	Design	Outcome	Place	Population	Age group	N	Risk factors or interventions	Effect : OR (CI), RR (CI) HR (CI), aHR (CI)
Coburn 1960 [3]	Cross-section	ARF incident case	USA	Families surveyed in the Bridgeport district to identify rheumatic children, cases verified by clinicians.<br>Controls had no history of ARF/RHD.	7-15 years	Cross section 1039, cases 153, controls 886	**Univariate:**<br>Low egg consumption<br>Other dietary factors (low consumption of milk, protein vitamins A&C)	 p<0.01<br>NS
Adanja et al 1988 [4]*	Case-control	ARF incident case	Yugoslavia	Cases: ARF patients identified through reports to health administration within 1 year of their first attack.<br>Controls: Healthy participants matched for age, sex, place of residence	Adults and children (95% participants <20 years old)	148 cases, 444 controls	**Univariate:**<br>Living space <5m2<br>≥ 2 people per room<br>≥ 2 people per bed<br>Deteriorated house<br>Damp house<br>Other poor housing features<br>Low education of mother<br>Change in place of residence in last 5 years<br>History of frequent sore throat<br>Family history of ARF<br>Parental unemployment	 RR 2.83 (1.19 - 6.71)<br>RR 1.72 (1.08 - 2.72)<br>RR 1.65 (1.02 - 2.66)<br>RR 1.83 (1.12 - 2.98)<br>RR 2.48 (1.34 - 4.61)<br>RR 2.58 (1.38-4.83)<br>RR 2.58 (1.38 - 4.83)<br>RR 5.00 (1.52 - 7.93)<br>RR 2.01 (1.41 - 2.89)<br>RR 2.81 (1.68 - 4.69)<br>RR 10.37 (5.31 - 20.24)
Bahr 1989 [5]	Case-control	ARF incident cases	Kuwait	Cases: ARF diagnosis based on the revised (1965) Jones Criteria.<br>Controls: University and hospital staff	Cases: children. Control: Adults	Cases: 39<br>Controls: 90	**Univariate:**<br>Vitamin D-binding protein Gc2 allele (2x more common in cases)	 p = 0.0024 (risk measure not given)
Vlajinac 1989 [6]*	Case-control	ARF incident case	Yugoslavia (same study populatio	Cases: ARF patients identified through reports to health administration	Adults and children (95%	148 cases, 444 controls	**Univariate:**<br>ARF in family<br>Under-nourishment	 RR 2.78 (1.67-4.63)<br>RR 2.18 (1.33-3.58)

Author, year, ref	Design	Outcome	Place	Population	Age group	N	Risk factors or interventions	Effect :<br>OR (CI), RR (CI)<br>HR (CI), aHR (CI)
			n as above: Adanja, 1988)	within 1 year of their first attack.<br>Controls: healthy participants matched for age, sex, place of residence	participants <20 years old)		>2 people per room<br>Sharing bed<br>Home dampness<br>Low education of mother	RR 1.60 (1.05-2.44)<br>RR 1.43 (1.04-2.13)<br>RR 2.70 (1.31-4.28)<br>RR 2.01 (1.18 - 3.41)
Vlajinac 1991 [7]*	Case-control	ARF incident case	Yugoslavia (same study population as above: Adanja, 1988	Cases: ARF patients identified through reports to health administration within 1 year of their first attack.<br>Controls: healthy participants matched for age, sex, place of residence	Adults and children (95% participants <20 years old)	Cases 148, Controls 444	**Multivariate:**<br>Home dampness<br>Change in place of residence in last 5 years<br>Body weight below normal<br>Frequent sore throat<br>Low education of mother<br>Family history for ARF<br>Space per person<5msq<br>>2 people per room<br>Sharing bed<br>Unemployed parents	 RR 2.40 (1.26- 4.58)<br>RR 3.62 (1.15-11.35)\<br>RR 1.42 (1.08- 1.86)<br>RR 2.26 (1.49- 3.39)<br>RR 2.52 (1.29- 4.92)<br>RR 2.98 (1.68- 5.29)<br>RR 1.72 (0.69- 4.25)<br>RR 1.35 (0.61- 3.00)<br>RR 1.04 (0.99- 1.09)<br>RR 2.08 (0.85- 5.09)
Adanja 1991 [8] *	Case-control	ARF incident case	Yugoslavia (same study population as above: Adanja, 1988	Cases: ARF patients identified through reports to health administration within 1 year of their first attack.<br>Controls: healthy participants matched for age, sex, place of residence	Adults and children (95% participants <20 years old)	Cases 148, Controls 444	**Univariate**<br>Food consumption<br>Meat <3 times/wk<br>No milk<br>No cheese<br>No eggs<br>Vegetables <3 times/wk<br>No fruit<br>Body weight:<br>Below normal<br>Above normal<br>**Multivariate:**	 <br>1.10 (0.73-1.66)<br>1.53 (0.91-2.57)<br>1.46 (0.83-2.57)<br>1.83 (0.65-5.13)<br>1.69 (0.56-5.13)<br>1.53 (0.64-3.65)<br><br>1.94 (1.23-3.07)<br>1.01 (0.67-1.53)<br><br>2.23 (1.09-4.53)

Author, year, ref	Design	Outcome	Place	Population	Age group	N	Risk factors or interventions	Effect : OR (CI), RR (CI) HR (CI), aHR (CI)
							Body weight below normal<br>No milk in diet	1.28 (0.97-1.63)
Thomas, 1994 [9]	Cross-section	ARF or scarlet fever history	United Kingdom	Farmworkers: Cases with history of ARF or scarlet fever (8); Controls with no history of these illnesses	16-75 years	Cases: 8 (ARF: 2, Scarlet Fever: 6)<br>Controls: 396	**Univariate:**<br>Exposure to dairy cattle<br>Drinking raw milk	 RR 0.12 (0.02-0.99)<br>RR 0.10 (0.01-0.85)
Zaman 1997 [10] **	Case-control	ARF incident case	Banglades h	Cases with ARF, Controls with recent GAS infection	5-23 years	Cases: 44<br>Controls: 86	**Univariate:**<br>Low income<br>Substandard house<br>Low height for age<br>Low body weight for age<br>Small dwelling space<br>Large family size<br>Age:<br><9 years<br>15-19 years<br>>19 years<br>Female gender<br>**Multivariate:**<br>Substandard house<br>Family size >6 persons<br>Low height for age	 OR 2.37 (1.04-5.46)<br>OR 2.93 (1.23-7.15)<br>OR 2.68 (1.14-6.34)<br>OR 1.36 (0.64-2.99)<br>OR 2.14 [0.93-4.96<br>OR 2.03 (0.94-4.39)<br>OR 1.19 (0.48-2.89)<br>OR 1.52 (0.56-4.01)<br>OR 1.52 (0.35-5.98)<br>OR 0.59 (0.28-1.23)<br>OR 3.18 (1.24-8.44)<br>OR 2.08 [0.88-5.041<br>OR 2.68 (1.06-6.86)
Zaman 1998 [11] **	Case-control	ARF incident case	Banglades h	Cases diagnosed with ARF using Jones Criteria,<br>Controls were hospitalised patients who did not meet the Jones criteria.<br>Both cases and controls showed	5-20 years	Cases: 60<br>Controls: 104	**Univariate:**<br>Higher number of siblings<br>Larger family size<br>Sharing bedroom<br>Higher number of people per room<br>Less parental schooling	 (p=0.001, t-test)<br>(p=0.89, t-test)<br>(p=0.26, t-test)<br>(p=0.58, t-test)<br>(p<0.0001, t-test)<br>(p=0.002, t-test)

Author, year, ref	Design	Outcome	Place	Population	Age group	N	Risk factors or interventions	Effect : OR (CI), RR (CI) HR (CI), aHR (CI)
				evidence of recent β-haemolytic streptococci infection.			Low income<br>Low height for age<br>Low weight for age<br>Small upper arm circumference<br>Food intake low in:<br>Egg<br>Milk<br>Beef<br>Mutton<br>Chicken<br>Fish<br>Pulses<br>Fruits<br>Parana<br>Ruli (bread)<br>Leafy vegetables<br>Other vegetables<br>Soybean<br>**Multivariate:**<br>Low height for age<br>Low weight for age<br>Small upper arm circumference<br>Food intake low in:<br>Egg<br>Milk<br>Beef<br>Mutton<br>Chicken<br>Fish<br>Pulses	OR 3.83 (1.74-8.40)<br>OR 2.41 (1.12-5.57)<br>OR 3.76 (l.87-7.89)<br>OR 3.81 (1.95-7.63)<br>OR 2.60(1.36-5.08)<br>OR 1.67 (0.87-3.20)<br>OR 1.33 (0.68-2.65)<br>OR 2.62 (1.35-5.21)<br>OR 1.07 (0.56-2.06)<br>OR 1.98 (1.03-3.84)<br>OR 2.29 (1.20-4.45)<br>OR 1.37 (0.71-2.66)<br>OR 3.15 (1.61-6.34)<br>OR 1.02 (0.51-2.01)<br>OR 1.71 (0.99-3.27)<br>OR 0.28 (0.12-0.62)<br>OR 2.23 (0.97-5.53)<br>OR 1.47(0.58-3.92)<br>OR 2.40 (1.04-5.77)<br>OR 2.29 (1.01-5.27)<br>OR 1.55 (0.66-3.61)<br>OR 1.11 (0.49-2.48)<br>OR 1.05 (0.46-2.38)<br>OR 1.60 (0.69-3.74)<br>OR 1.08 (0.48-2.47)<br>OR 1.48 (0.67-3.31)<br>OR 1.66 (0.74-3.74)<br>OR 0.81 (0.34-1.88)

Author, year, ref	Design	Outcome	Place	Population	Age group	N	Risk factors or interventions	Effect : OR (CI), RR (CI) HR (CI), aHR (CI)
							Fruits	OR 2.15 (0.96-4.85)
							Parana	OR 0.96 (0.42-2.15)
							Ruli (bread)	OR 1.38 (0.63-3.03)
							Leafy vegetables	OR 0.42(0.15-1.13)
							Other vegetables	
							Soybean	
Zaman 1998 [12] **	Case-control	ARF incident case	Bangladesh	Cases diagnosed with ARF using Jones Criteria,<br>Controls were hospitalised patients who did not meet the Jones criteria.<br>Both cases and controls showed evidence of recent β-haemolytic streptococci infection.	5-20 years	Cases: 44<br>Controls: 44	**Univariate:**<br>Higher total protein<br>Higher albumin<br>Higher cholesterol (total)<br>Higher HDL<br>Higher LDL<br>Higher Triglycerides<br>Higher Haemaglobin<br>Higher Packed cell Volume<br>Higher Iron<br>Higher Total iron binding capacity<br>Higher Transferrin saturation<br>**Multivariate**<br>Higher Total protein<br>Higher Albumin<br>Higher Cholesterol (total)<br>Higher HDL<br>Higher LDL<br>Higher Triglycerides<br>Higher Haemaglobin<br>Higher Packed cell volume<br>Higher Iron	OR 1.06 (0.98 - 1.15)<br>OR 0.72 (0.59 - 0.88)<br>OR 0.80 (0.41 - 1.57)<br>OR 0.05 (0.01 - 0.49)<br>OR 1.44 (0.65 - 3.22)<br>OR 0.50 (0.15 - 1.73)<br>OR 0.94 (0.90 - 0.99)<br>OR 0.73 (0.59 - 0.91)<br>OR 0.81 (0.71 - 0.93)<br>OR 0.95 (0.91 to 1.00)<br>OR 0.89 (0.83 to 0.96)<br>OR 1.02 (0.92 - 1.14)<br>OR 0.75 (0.60 - 0.95)<br>OR1.20 (0.46 - 3.16)<br>OR 0.38 (0.03 - 5.48)<br>OR 1.56 (0.51 - 4.74)<br>OR 0.86 (0.15 - 4.82)<br>OR 0.96 (0.91 - 1.02)<br>OR 0.69 (0.50 - 0.97)<br>OR 0.82 (0.68 - 0.97)<br>OR 0.96 (0.91 - 1.04)<br>OR 0.90 (0.82 - 0.98)

Author, year, ref	Design	Outcome	Place	Population	Age group	N	Risk factors or interventions	Effect : OR (CI), RR (CI) HR (CI), aHR (CI)
							Higher total iron binding capacity<br>Higher transferrin saturation	
Berdeli 2004 [13] #	Case-control	ARF incident case	Turkey	Cases: Caucasian Turkish ARF patients meeting Jones Criteria at a University paediatric clinic. Controls: healthy child volunteers	Cases: 10.98 ±2.9 years, Controls : 8.71±1.3 (mean age ± SD)	Cases: 66, controls: 117	**Univariate:**<br>FcgammaRIIA-R/H-131 polymorphisms:<br>RR genotype (Present in 66.7% cases, 39.3% controls)<br>HR heterozygosity (Present in 21.2% cases, 5.1% controls)	 <br>OR 4.98 (1.81-13.70)<br>OR 3.09 (1.64-5.81)
Berdeli 2005 [14] #	Case-control	ARF incident case	Turkey	Cases: Caucasian Turkish ARF patients meeting Jones Criteria at a University paediatric clinic. Controls: healthy child volunteers.	Cases: 11.16±2.88 years, Controls : 8.71±1.3 (mean age ± SD)	Cases: 61, controls: 91	**Univariate:**<br>Arg753Arg genotype (TLR-2 polymorphism, present in 8.2% cases, 90.2% controls)<br>Arg753Gln genotype (TLR-2 polymorphism, present in 91.8% cases, 9.9% controls)<br>Presence of TLR-2 Gln allele	 OR 0.01 (0.00-0.03)<br>OR 100.00 (32.00–320.00)<br>OR 16.0 (7.6–35.00)
Berdeli 2006 [15] #	Case-control	ARF incident case	Turkey	Cases: Caucasian Turkish ARF patients meeting Jones Criteria at a University paediatric clinic. Controls: healthy adult volunteers	Cases: 10.98 ±2.9 years, Controls : 27.86±6.8 years (mean age ± SD)	Cases: 66, controls: 107	**Univariate:**<br>TNFalpha polymorphisms at -308	 No association

Author, year, ref	Design	Outcome	Place	Population	Age group	N	Risk factors or interventions	Effect : OR (CI), RR (CI) HR (CI), aHR (CI)
Kurahara 2006 [16]	Case-control	ARF incident case	Hawaii	Cases: ARF cases Controls: Other heart condition at Paediatric cardiology clinic.<br>All qualitied for Medicare	NS	Cases 26, Controls 41	**Univariate:**<br>Polynesian ethnicity<br>Polynesian and higher number of children per household<br>More children in case bedroom<br>Low parental education	 OR 4.80 (1.65-15.63)<br>OR 6.53 (1.90-24.10)<br>NS<br>NS
Hounie 2007 [17]	Case-control	ARF incident case	Brazil	Cases: ARF outpatients attending a clinic and first degree relatives, Controls: orthopaedic outpatients attending a clinic and first degree relatives. University of Sao Paulo Medical School.	Case probands: Mean age 14.36 years (±4.60),<br>Control probands: Mean age 11.51 ±3.29	Cases: 310, controls: 177	**Univariate:**<br>Obsessive-compulsive spectrum disorders (ARF cases and first degree relatives)	 OR 2.21 (1.09-4.49)
Seixas 2008 [18]	Case-control	ARF incidence in family	Brazil	Cases: ARF cases or first degree family members presenting to RF Outpatient Clinic,<br>Controls: patients or first degree family members presenting to Orthopedic Outpatient Clinic.	≥16 years old	Cases: 188 Controls:96	**Univariate:**<br>Generalised anxiety disorder	 1.71 ($p<0.05$)<br>95%CI not supplied
Messias-Reason 2009 [19]	Case-control	ARF and RHD prevalent case	Brazil	Cases: History of ARF or RHD attending out-patients clinic<br>Controls: healthy subjects from the	8-76 years	Cases: 244 (ARF=82, RHD=162)<br>Controls: 420	**Univariate:**<br>FCN2 haplotype AGA	 OR: 0.32 (0.13–0.77)

Author, year, ref	Design	Outcome	Place	Population	Age group	N	Risk factors or interventions	Effect : OR (CI), RR (CI) HR (CI), aHR (CI)
				same geographic region and socioeconomic background (not age matched).				
Walker 2011 [20]	Case-control	ARF incident case	South Africa	Cases: Index cases of ARF<br>Controls: recruited from outpatient clinics at the time of venesection for other indications	Not stated in paper	Cases: 40<br>Controls: 47	**Univariate:**<br>B-cells expressing D8/17	Mean difference between cases and controls cell % (not OR): 0.23 (95% CI: 0.02–0.43, p<0.05). Risk measure not given.
Col-Araz 2012 [21]	Case-control	ARF incident case	Turkey	Cases: ARF patients followed in the Pediatric Cardiology Clinic,<br>Controls: healthy children referring to the Well-Child Outpatient Clinic for routine health checkups	5-15 years	Cases: 38, controls:40	**Univariate:**<br>IFN- γ (+874) TT genotype<br>IFN- γ (+874) gene T allele<br>IFN- γ (+874) gene A allele	 OR 8.10 (2.41–27.27)<br>OR 3.02 (1.57–5.79)<br>OR 0.33 (0.17–0.64)
Riaz 2013 [22]	Case-control	ARF incident cases and RHD prevalent cases (echocardiography)	Bangladesh	Cases: Patients referred to National Centre for Control of Rheumatic Fever and Heart Disease, Controls: from same center or patients at Shaheed Suhrawardy Medical College Hospital with no ARF or RHD.	Not stated, no age limits implied	RF 103, RHD 102, Controls 207	**ARF risk factors:**<br>**Multivariate:**<br>Age >19 yo<br>Sex (female)<br>Urban residence<br>Wall material (brick)<br>Family size (>5 people)<br>>2 siblings<br>Higher family income<br>Maternal illiteracy<br>Maternal employment<br>>3 people sharing living room<br>Water supply (tubewell)	 <br>OR 0.1 (0.03-0.1)<br>OR 2.2 (1.1-4.3)<br>OR 3.1 (1.2-8.4)<br>OR 3.6 (1.6-8.1)<br>OR 0.3 (0.2-0.7)<br>OR 3.1 (1.5-6.3)<br>OR 0.9 (0.4-1.8)<br>OR 2.6 (1.2-5.8)<br>OR 7.0 (2.0-24.2)<br>OR 1.2 (0.6-2.4)

Author, year, ref	Design	Outcome	Place	Population	Age group	N	Risk factors or interventions	Effect : OR (CI), RR (CI) HR (CI), aHR (CI)
							Bed (floor)	OR 1.1 (0.4-2.6)
							Using toothpaste	OR 0.9 (0.3-3.4)
							Toothbrushing ≤1/day	OR 0.6 (0.2-1.7)
							Not tooth brushing after a meal	OR 0.9 (0.4-1.7)
								OR 2.5 (1.0-6.3)
							**RHD risk factors** (non-rheumatic controls):	
							**Multivariate:**	
							Age >19 yo	OR 0.1 (0.1-0.3)
							Sex (female)	OR 2.2 (1.2-4.2)
							Urban residence	OR 2.0 (1.2-7.0)
							Wall material (brick)	OR 2.8 (1.3-5.3)
							Family size (>5 people)	OR 0.5 (0.2-0.9)
							>2 siblings	OR 4.4 (2.2-8.7)
							Maternal illiteracy	OR 2.5 (1.2, 4.9)
							Maternal employment	OR 6.2 (2.1,18.4)
							>3 persons sharing living room	OR 1.9 (1.0-3.4)
							Water supply (tubewell)	OR 1.6 (0.7-3.6)
							Bed (floor)	OR 1.5 (0.5-4.1)
							Toothpaste	OR 0.7 (0.2-1.8)
							Toothbrushing ≤1/day	OR 1.3 (0.7-2.4)
							Not tooth brushing after a meal	OR 1.5 (0.6-3.9)
Thornley 2016 [23] ^	Cohort	ARF or RHD incident case	Auckland, NZ	20,333 children in Auckland who were free of RHD at enrolment were followed for a mean of 5 years, cases hospitalised with ARF or RHD, controls remained free of ARF and RHD.	Participants aged 5-6 years at first dental visit.	Cases 96, Controls 20,237	**Multivariate:** 5+ primary teeth affected by caries	aHR 1.57 (1.20 -2.06)

Author, year, ref	Design	Outcome	Place	Population	Age group	N	Risk factors or interventions	Effect : OR (CI), RR (CI) HR (CI), aHR (CI)
Thornley 2018 [24] ^	Cohort	ARF or RHD incident case	Auckland, NZ	213,957 children free of RHD at baseline, mean follow-up time 5.1 years. Cases ARF or RHD, controls remained free of ARF and RHD.	Participants aged 3-12 years at first dental Visit.	Cases 440, controls 213,517	**Multivariate**<br>Children diagnosed with scabies during hospital admissions	aHR 8.98 (6.33 -20.2)
**RHD**								
Caughey 1975 [25]	Case-control	ARF or RHD prevalent case	NZ	Cases: 50 Maori and 50 Europeans with ARF or RHD<br>Controls: 75 Maori and 514 European disease-free blood donors		50 Maori and 50 Europeans with ARF/RHD compared with each control group	**Univariate:**<br>European case:<br>HL-A28 reduced<br>HL-A17 increased<br>Maori cases: minor differences in frequency of HL-A3 increased<br>HL-A8 increased<br>HL-A10 decreased	 <br>RR 0.05 (0.00-0.90)<br>RR 4.55 (2.12-9.77)<br><br>RR 7.78 (0.37-165.63)<br>RR 6.43 (0.70-59.37)<br>RR 0.09 (0.01-0.70)
McLaren 1975 [26]	Cross sectional	RHD prevalent case (clinical)	South Africa	Pre-school and school children (Soweto).<br>Cases: RHD diagnosed using clinical auscultation screening,<br>Controls had no RHD on clinical auscultation.	2-18 years	Cross section 12,050 total children, RHD cases: 80, controls: 11,970	**Univariate:**<br>Siblings >3<br>Language group<br>School area<br>Local area prevalence of pharyngeal GAS carriage<br>Socioeconomic status<br>No. people sharing bedroom	 p<0.05<br>p>0.05<br>p>0.05<br>p>0.05<br><br>p>0.05<br>p=0.05
Anabwani 1989 [27]	Cross-sectional	RHD prevalent case	Kenya	School children, semi-rural area (Emuhaya). Cases: Identified as having RHD using	5-15 yo	Cross section 3631, RHD 6, Controls 3625	**Univariate**<br>None identified (including socioeconomic status, family size, no. children per bedroom).	 Nil

Author, year, ref	Design	Outcome	Place	Population	Age group	N	Risk factors or interventions	Effect : OR (CI), RR (CI) HR (CI), aHR (CI)
				echocardiography screening, Control: No RHD on echocardiography screening.				
Coggon 1993 [28]	Cohort (retro-spective)	RHD mortality during 1951 to 1989	UK	Chesterfield township address in 1936 housing survey and 1939 census. Cases identified in mortality records using ICD-9 coding to identify RHD as cause of death, Controls: RHD not listed as cause of death	No age limits implied	Cases: 76 Controls: 8,062	**Univariate** 1.5-2.49 persons per bedroom (compared with <1.5) >2.49 persons per bedroom (compared with <1.5)	RR 0.9 (0.5-1.6) RR 0.7 (0.3-1.6)
Longo-Mbenza 1998 [29]	Cross-sectional	RHD prevalent case (echocardiography)	Democratic Republic of Congo	Children living in Kinshasa town and adjoining slums. Cases: Identified as having RHD using echocardiography screening, Control: No RHD on echocardiography screening.	5-16 years	Cross section: 4848 children; RHD cases 68, controls 4780.	**Multivariate:** Birth in rainy season (vs. dry season) Low birthweight Low SES vs higher SES Low BMI >8 in household Migrant (vs Kongo native)	RR 2.2 (0.97–4.90) RR 1.81 (1.04–3.15) RR 2.68 (1.43–5.01) RR 2.64 (1.48–4.70) RR 4.10 (1.70–9.85) RR 4.79 (2.14–10.68)
Oli 1999 [30]	Cross-sectional	RHD prevalent case	Ethiopia	School children in an urban area. Cases: Identified as having RHD using echocardiography screening, Control: No RHD on echocardiography screening.	10-15yo	Cross section 9378, RHD cases 60, Controls: 9,318	**Univariate:** Sex (female) Low SES and Crowding conditions at home, in schools and in the bedrooms were not associated with risk of RHD after adjusting for confounders	OR 1.76 (1.01 – 3.06)

Author, year, ref	Design	Outcome	Place	Population	Age group	N	Risk factors or interventions	Effect : OR (CI), RR (CI) HR (CI), aHR (CI)
Rizvi 2004 [31]	Cross-sectional	RHD prevalent case	Pakistan	Rural population (Rahim Yaar Khan district)<br>Cases: Identified as having RHD using echocardiography screening,<br>Control: No RHD on echocardiography screening.	Not stated, no age limits implied, 50% pop. <15 yo.	Cross section 9430 screened, RHD cases 54, Controls: 9,376	**Univariate:**<br>Older mean age (29.5 years cases/20.9 years controls)<br>Female gender<br>Home crowding (people per room)<br>House construction<br>House standard<br>Lower SES<br>Case education level	p<0.05<br>RR 1.86 (1.07-3.24)<br>NS<br>NS<br>NS<br>NS<br>NS
Chou 2004 [32]	Case-control	RHD prevalent case	Taiwan	Cases: RHD confirmed by echocardiography<br>Controls: age- and sex-matched unrelated healthy volunteers	26-80 years	Cases: 115, controls: 100	**Univariate:**<br>ACE I/D II genotype<br>ACE I allele	OR 2.12 (1.21-3.71)<br>OR 1.50 (1.02-2.21).
Messias-Reason 2009 [19]	Case-control	RHD prevalent case	Brazil	Cases: Valvular RHD lesions confirmed on echocardiography in out-patients from Children's Cardiologic Unit and Cardiology Out-patient Clinic<br>Controls: healthy subjects from the same geographic region and socioeconomic background.	8-76 years	Cases: 106<br>Controls: 210	**Univariate:**<br>FCN2 haplotype GGA<br>FCN2 haplotype AGA<br>FCN2 haplotype AGG<br>(compared with haplotype AAA)	OR 1.56 (1.10–2.30)<br>OR 0.32 (0.13-0.77)<br>OR 0.44 (0.23-0.82)

Author, year, ref	Design	Outcome	Place	Population	Age group	N	Risk factors or interventions	Effect : OR (CI), RR (CI) HR (CI), aHR (CI)
Steer 2009 [33]	Cross-sectional	RHD prevalent case	Fiji	Cases: RHD confirmed by echocardiography, Controls: No RHD on echocardiography.	5-15 years	Cross section 3462, RHD cases 359, Controls: 3103	**Univariate**<br>Indigenous Fijian ethnicity<br>Rural school<br>Lower weight for age<br>Lower height for age<br>Lower BMI for age<br>Older age<br>**Multivariate**<br>Female gender<br>Indigenous Fijian ethnicity<br>Rural school<br>Impetigo<br>Scabies	RR 2.3 (0.97-5.6)<br>RR 1.8 (1.0-3.1)<br>p>0.05<br>p>0.05<br>p>0.05<br>p>0.05<br>RR 1.6 (0.8-3.2)<br>RR 2.0 (0.8-5.1)<br>RR 1.3 (0.7-2.4)<br>RR 1.7 (0.7-4.2)<br>RR 1.0 (0.4-2.4)
Azevedo 2010 [34]	Case-control	RHD prevalent case	Brazil	Cases: ARF patients who fulfilled the Jones criteria with features of RHD Controls: race-matched, healthy blood donors	Cases: 7-41 years, controls > 35 years old.	Cases 84, controls 84	**Univariate:**<br>Polymorphism of the IL-1ra gene is a relevant factor for RHD severity	Allele 1 and genotype A1/A1 less frequent among patients with severe carditis compared with patients without this manifestation (OR=0.11, p=0.031; OR=0.092, p=0.017)
Saxena 2011 [35]	Cross-sectional	RHD prevalent case	India	School children living in rural Northern India. Cases: Identified as having RHD using echocardiography screening, Control: No RHD on echocardiography screening.	5-15 years	Cross section: 6270, RHD cases 128, Controls 6142	**Univariate:**<br>Older age<br>Female gender<br>Studying in government funded school<br>Substandard house<br>Home crowding<br>Height<br>Weight <25th percentile, Low BMI	p<0.001<br>p<0.001<br>p<0.001<br>p<0.005<br>p<0.005<br>p<0.05<br>p<0.05<br>p<0.05

Author, year, ref	Design	Outcome	Place	Population	Age group	N	Risk factors or interventions	Effect : OR (CI), RR (CI) HR (CI), aHR (CI)
							Waist circumference<br>History of joint pain<br>Auscultatory abnormalities<br>**Multivariate:**<br>Older age<br>Female gender<br>Government school<br>Substandard house<br>Home crowding	p<0.05<br>p<0.05<br>p<0.05<br>OR 1.93 (1.29-2.88)<br>OR 1.84 (1.25 -2.72)<br>OR 1.55 (1.02 - 2.34)<br>OR 1.34 (0.84 – 2.17)<br>OR 1.16 (0.75 - 1.78)
Ba-Saddik 2011 [36]	Cross-sectional	RHD prevalent case	Yemen	School children.<br>Cases: Identified as having RHD using echocardiography screening,<br>Control: No RHD on echocardiography screening	5-16 yo	Cross section 6000, RHD cases 219, controls 5781	**Univariate:**<br>Older age<br>Home crowding (>4 per bedroom)<br>Low household income<br>Poor housing conditions (ie. home not constructed with stone and lacked water supply)	RRs not calc. but association tested<br>p=0.001<br>p<0.001<br>p<0.001<br>p<0.001
Dobson 2012 [37]	Case-control (nested in cross-sectional study)	RHD prevalent case (echocarid iogram)	Fiji	School children in rural and urban settings who participated in a previous screening study. Cases with documented RHD, Controls age & sex matched without RHD	5-15yo	Cases 80, Controls 80	**Univariate:**<br>Trend toward increased risk of RHD and poor-quality housing, lower SES (but not stat. sig.)<br>People in home<br>People per bedroom<br>Children per classroom<br>Maternal unemployment<br>Maternal education	NS<br>NS<br>NS<br>OR 2.6 (1.2–5.8)<br>NS
Okello 2012 [38]	Case-control	RHD prevalent case	Uganda	Patients seen at the Mulago National Referral Hospital: cases were diagnosed with	5-60 years	Cases 243, Controls 243	**Univariate:**<br>People per house >8<br>Space per person < 90 feet^2	OR 1.98 (1.4–2.5)<br>OR 8.3 (6.1–10.4)

Author, year, ref	Design	Outcome	Place	Population	Age group	N	Risk factors or interventions	Effect : OR (CI), RR (CI) HR (CI), aHR (CI)
				RHD, controls had normal echocardiograms.			Longer distance to nearest health unit<br>Education level (compared to <primary):<br>Primary<br>Secondary<br>Vocational<br>University<br>Unemployed<br>Income (compared with <25USD)<br>25-49.5USD<br>50-99.5USD<br>100USD+<br>Male gender<br>**Multivariate:**<br>Space per person < 90 square feet<br>Longer distance to nearest health unit<br>Higher number of people living in the house<br>Education level (compared to <primary):<br>Primary<br>Secondary<br>Vocational<br>University<br>Unemployed	OR 1.48 (0.2–3.2)<br>OR 0.38 (0.17–0.83)<br>OR 1.28 (0.59–2.74)<br>OR 1.47 (0.45–4.79)<br>OR 2.19 (0.95–5.09)<br>OR 3.09 (2.04–4.72)<br>OR 1.95 (0.9–4.2)<br>OR 2.19 (0.96–4.98)<br>OR 14.7 (5.96–36.1)<br>OR 1.26 (0.82–1.95)<br>OR 1.35 (1.10–1.56)<br>OR 0.70 (0.61–0.87)<br>NS<br>OR 0.57 (0.22–1.43)<br>OR 1.91 (0.75–4.88)<br>OR 1.8 (0.45–7.21)<br>OR 2.93 (1.04–8.19)<br>OR 1.71 (1.05–8.19)
Rehman 2013 [39]	Case-control	RHD prevalent case	Pakistan	Cases: patients with RHD, Controls: healthy individuals from similar ethnic groups and	Cases: mean age 30 ± 14.5 years,	150 RHD cases, 204 controls	TNF-alpha(-308):<br>G/G genotype<br>G/A genotype<br>A/A genotype	OR 0.39 (0.20-0.76)<br>OR 1.97 (0.98–3.97)

Author, year, ref	Design	Outcome	Place	Population	Age group	N	Risk factors or interventions	Effect :<br>OR (CI), RR (CI)<br>HR (CI), aHR (CI)
				geographic area as cases<br>[aim to investigate the role of cytokine gene polymorphisms and their potential usefulness as biomarkers in RHD].	controls: 45 ± 12.7 years.		G allele<br>A allele<br>IL-6-174:<br>G/G genotype<br>G/C genotype<br>C/C genotype<br>G allele<br>C allele<br>IL-10–1082:<br>G/G genotype<br>G/A genotype<br>A/A genotype<br>G allele<br>A allele<br>IL-Ra VNTR:<br>A1/A2 genotype<br>A1/A3 genotype<br>A2/A3 genotype<br>A1 allele<br>A2 allele<br>A3 allele	OR 9.94 (1.21–217.31)<br>OR 0.35 (0.20–0.64)<br>OR 2.81 (1.55–5.14)<br>OR 2.6 (1.17–5.85)<br>OR 0.92 (0.56–1.52)<br>OR 0.76 (0.48–1.20)<br>OR 1.5 (1.04–2.16)<br>OR 0.67 (0.46–0.96)<br>OR 1.06 (0.66–1.71)<br>OR 1.30 (0.81–2.07)<br>OR 0.73 (0.46–1.17)<br>OR 1.18 (0.87–1.61)<br>OR 0.85 (0.62–1.15)<br>OR 1.72 (0.92–3.24)<br>OR 1.37 (0.34–5.58)<br>Not calculable<br>OR 0.52 (0.31–0.86)<br>OR 1.50 (0.61–3.83)<br>OR 0.0 (0.0–5.54)
Eriksson 2013 [40]	Cohort (retro-spective)	RHD hospitalisa tions and death	Helsinki, Finland,	Births in Helsinki maternity hospitals 1924-1944, cases identified using hospital and mortality records.<br>Cases: Received a diagnosis of RHD,<br>Controls: Not diagnosed with RHD.	25-80 yo	Cohort 20,431, RHD cases 101, Controls 20330	**Univariate:**<br>Long umbilical cord (risk found for RHD mitral disease only)<br>No. people living in home (compared with ≤3 people):<br>4 people<br>5 people<br>≥6 people<br>People per room	 HR 1.23 (1.04-1.45)<br><br>HR 2.1 (1.1-4.0)<br>HR 2.1 (1.1-4.2)<br>HR 2.1 (1.1-4.3)<br>NS

Author, year, ref	Design	Outcome	Place	Population	Age group	N	Risk factors or interventions	Effect : OR (CI), RR (CI) HR (CI), aHR (CI)
Mirabel 2015 [41]	Cohort (prospective)	RHD persistence in an RHD cohort compared with controls	New Caledonia	4th grade school children, RHD cases diagnosed using echocardiography, controls selected randomly from classmates without RHD, matched for ethnicity and classroom	9-10 years	RHD cases 114, Controls 227	**Univariate**<br>Number of siblings<br>House construction<br>Mother's education<br>Maternal employment<br>Male gender<br>Oceanic ethnicity<br>Lived >1year out of household<br>Usual mode of transport: private car<br>Number of people per bedroom >2<br>**Multivariate :**<br>≥3 per bedroom<br>Mother's education:	p=0.2<br>p=0.9<br>p=0.048<br>p=0.4<br>p=0.3<br>p=0.9<br>p=0.2<br>p=0.4<br>p=0.003<br>OR 8.27 (1.67–41.08)<br>OR 1.97 (0.69–5.64)
Azevedo 2016 [42]	Case-control	RHD prevalent case	NZ	Cases: confirmed RHD and of Māori and Pacific ancestry, controls: Polynesian/Maori ancestry and no autoimmune condition	Cases aged 3-32 years old at first ARF presentation, control age range not stated	204 RHD cases, 116 controls	**Univariate:**<br>Variant of IL6 promoter (rs1800797 (-597G/A)) with RHD IL1RN variant (rs447713) with the severity of carditis	GG genotype 6.09 (CI 1.23; 30.23) times more likely to develop RHD than carriers of AA genotype (P=0.027).<br>G allele (GG plus AG genotype) for IL1RN rs447713 SNP had 2.36 times (CI 1.00; 5.56) more severe carditis than those without this allele (the AA genotype) (P=0.049)
Parks 2017 [43]	GWAS meta-analyses	RHD prevalent case	Eight Oceanian countries	Melanesians (607 cases and 1,229 controls);	Age limits not	2,852 individuals.	**GWAS meta-analysis:**<br>Immunoglobulin heavy chain locus - IGHV4-61	Each copy of IGHV4-61*02 associated with a

Author, year, ref	Design	Outcome	Place	Population	Age group	N	Risk factors or interventions	Effect : OR (CI), RR (CI) HR (CI), aHR (CI)
				Polynesians, South Asians and Mixed or other populations (399 cases and 617 controls)	stated (none implied)		gene segment (IGHV4-61*02 allele)	1.4-fold increase in the risk of RHD (OR 1.43, 1.27-1.61, P=4.1 x 10-9)
Gray 2017 [44]	GWAS	RHD prevalent case	Australia	Aboriginal population	≥18 years old	1263 total, 398 RHD cases; 865 controls	**GWAS analysis:** HLA-DQ locus was strongest genetic marker associated with RHD, eg HLA-DQA1*0101_ DQB1*0503	OR = 1.44, CI = 1.09–1.90, P = 9.56×10^{-3}

The following groups of papers all drew on the same or overlapping study populations:

* Adanja 1988, Vlajinac 1989, Vlajinac 1991, Adanja 1991

** Zaman 1997, Zaman 1998, Zaman 1998

Berdeli 2004, Berdeli 2005, Berdeli 2006

^ Thornley 2016, Thornley 2018

Abbreviations: NS=Not Significant/No association

OR=Odds Ratio, RR=Relative Risk, HR=Hazard Ratio

GWAS=Genome-Wide Association Study

Table S3: Factors investigated in the ARF Risk Factors Study

Category of risk and protective factors	Examples of variables including in the study	Variables included in the primary analysis
**A. Preceding infection**		
Potential GAS infections	• Preceding sore throat<br>• Preceding skin infection<br>• Preceding scabies<br>• Skin cuts, grazes, wounds, insect bites	• Not included as these infections are considered a direct cause of ARF
**B. Environmental risk factors**		
High levels of social contact	• Attendance at social gatherings outside own home<br>• School size (number of pupils)<br>• Overseas travel and contact with overseas visitors	• Social gatherings outside home (composite of 9 Qu.)
Household crowding	• Occupancy (people per house)<br>• Density (people per room, people per 100m^2)<br>• Bedroom deficit (Canadian National Occupancy Standard (CNOS))<br>• Self-assessed 'overcrowding' and bedroom deficit	• Structural household crowding (people per room)
Functional crowding and bedroom crowding	• Functional crowding (eg sleeping in same room as others just to keep warm)<br>• Sleeping in communal area (eg living room)<br>• Sleeping with excess (≥ 2people) in bedroom	• Functional crowding (eg sleeping in same room as others just to keep warm)
Bed sharing and lack of standard bed	• Bed sharing with one or more others<br>• 'Hot bedding' (ie using bed others have slept in)<br>• Sleeping on floor or couch	• Bed sharing
Exposure to others with potential GAS	• Others in household with sore throat, cough, skin infection, scabies	• Not included

Household washing and laundry resources	• Lack of hot water for washing (delaying or having cold/lukewarm bath/shower)<br>• Availability and use of bath or shower<br>• Soap, own towel<br>• Laundry facilities, frequency bedding changed<br>• Regular swimming	• Lack of hot water for washing (composite of 2 Qu.)
House conditions	• Self-assessed house condition (eg poor or very poor)<br>• Age of house (eg build prior to 1980)	• Not included
House indoor environment, including cold, damp, mould and contributing factors	• Self-assessed cold<br>• Self-assessed damp, mould<br>• Insulation, Heating<br>• Power disconnections<br>• Ventilation, unflued gas heaters	• Cold (composite of 4 Qu.)<br>• Damp and mould (composite of 3 Qu.)
Tobacco smoke exposure	• Living with smokers (mother, other household members)<br>• Exposure in house/car<br>• Active smoker<br>• Hair nicotine levels	• Living with a smoker
Exposure to animals and fleas	• Domestic animals (cats, dogs)<br>• Fleas	• Not included
**C. Healthcare assess**		
Healthcare access	• Access barriers (timeliness, cost, transport, childcare)<br>• Has a usual GP or medical centre	• Barriers to accessing primary healthcare (composite of 5 Qu)
Dental healthcare access	• Regular dental checks, timely visits, access problems<br>• Has dental care provider<br>• Frequency of teeth brushing<br>• Personal toothbrush	• Not included
Sore throat and skin infection treatment	• Access to school-based RF Prevention Programme (RFPP)	• Attending school that provides RFPP

	• Consultation and treatment of recent sore throat / skin infection	
Healthcare knowledge (health literacy)	• Knowledge of ARF causes and consequences<br>• Knowledge and reported management of key conditions (sore throat, skin infection)	• Not included
**D. Health status and nutrition**		
Personal health history and status	• Self-assessed general health<br>• Asthma, eczema<br>• Tonsils and adenoids removed<br>• Frequency of previous hospitalisations, including infectious diseases (linked data)	• Not included
Oral health history and status	• Decayed, missing, and filled teeth (dmft/DMFT)(linked data)<br>• Self-assessed oral health status<br>• History of teeth filling and removal<br>• Gum disease (bleeding after brushing)	• Decayed, missing, and filled teeth (dmft of primary teeth/DMFT of permanent teeth)
Dietary intake	• Sugar-sweetened drink intake<br>• Fruit and vegetable intake<br>• Takeaway food intake	• Sugar-sweetened drink intake
Nutrient status	• Vitamin D levels<br>• Irons stores (Ferritin)	• Not included (only available on sample)
Body mass index (BMI)	• Height, weight, BMI	• Not included
**E. Host socio-economic determinants**		
Socioeconomic factors	• Area-based deprivation (NZDep)*<br>• Caregiver Individual deprivation (NZiDep)<br>• Caregiver education level<br>• Household income	• NZDep*<br>• NZiDep (caregiver)

Housing tenure and residential mobility	• Tenure (rented or owned)<br>• Short housing duration (<1year)<br>• Number of schools attended	• Tenure (rented or owned)
Location	• District Health Board (DHB)	• DHB*
**F Predisposing host factors**		
Demographic factors	• Age*, Sex*	• Age*, Sex*
Ancestry and genetic factors	• Ethnicity (prioritised)*<br>• Ancestry (ethnicity of grandparents)<br>• Born in a Pacific Island country<br>• Specific genetic markers (eg HLA-DRB1)	• Ethnicity*
Family history of ARF	• Family history of ARF	• Family history of ARF
Immunological factors	• Family size, Birth order<br>• Specific immunological markers	• Not included
Pregnancy and neonatal factors	• Pre-term delivery, Low birth weight<br>• Low APGAR score	• Not included
Breast feeding	• History of breast feeding,	• Not included
**F. Organism factors**		
Characteristics of GAS organisms	• Characterisation (*emm* typing, whole genome sequence) of GAS organisms isolated from throat	• Not included (only available on sample)
Exposure to infectious cofactors	• Nasal detection of *S. aureus*	• Not included (only available on sample)

*Used for matched controls

Supplementary Table S4: Potential risks to study effectiveness and their management

Potential Risk	Approach to Risk Management
1. **Missing key exposures from study design** - partly because of the complex nature of ARF as 2-stage autoimmune disease and lack of knowledge about its pathophysiology	• Extensive review of existing literature on likely risk factors for ARF<br>• Knowledge of the biology of Streptococcal bacteria and their modes of transmission<br>• Active review of study questionnaire and design by the multi-disciplinary research team
2. **Missing data** – at conclusion of study	• Direct data entry during interviews.<br>• Built in systems for monitoring data completeness to identify and correct data gaps and improve processes<br>• Optimise data collection and review completeness regularly, identify any items with high degree of missing data and take steps to improve completeness of collection<br>• Consider analytic approaches to missing data.
3. **Problems accessing dental records** - .	• Early communication and engagement with appropriate dental service contacts.<br>• Identification of data sources and logistics required to access records.<br>• Careful statement of project objectives, confidentiality measures and ethical consent when approaching dental health providers.
4. **Difficulties with data analysis** -	• Establish a detailed data analysis plan.<br>• Liaise with experienced epidemiologists.<br>• Keep record of methods and steps in analysis including log of analytical code used

5.	**Insufficient cases** -	• Maximise study recruitment through engagement with clinicians and utilising public health services as back-up to avoid missing cases<br>• Ensure study includes all DHBs with a moderate numbers of cases (eg 5+ expected during 2-year study period)<br>• Extend study period and/or increase number of matched controls per case if required
6.	**Selection bias of cases** ie ARF cases included in study are not representative of ARF cases generally in terms of exposures	• Should be low as ARF is uncommon and all diagnosed cases are hospitalised, notified, and intensely scrutinised, and we anticipate high participation rates among identified cases.<br>• Review case recruitment and if high proportion of eligible cases not participating investigate reasons and take steps to increase participation.<br>• Collect data on non-participating eligible cases and compare characteristics of consenting cases with non-consenting case to see if there are any systematic differences.
7.	**Selection bias of controls** ie controls included in study are not representative of source population in terms of important exposures eg population controls recruited for NZHS may be a relatively more 'stable' population than is typical; matched controls may be somewhat atypical as they are recruited from NZHS participants who agree to being re-contacted, who are still	• NZHS control population is recruited by CBG using a "probability-proportional-to-size (PPS) sampling design" that aims to give a sample population that is representative of the NZ population. Characteristics of the NZHS control population will be compared with the NZ Census population to see if there are any systematic differences (in terms of characteristics such as ethnicity and deprivation).<br>• Matched control population will be recruited from NZHS re-contact population in such a way as to have similar characteristics to cases for matched variables (age group, ethnicity, DHB, month of interview). Characteristics of these

	contactable, and who agree to being re-interviewed	matched controls will be compared with similar subjects in the original NZHS population to see if they differ in terms of important exposures (eg household crowding levels).<br>• Consider use of quantitative bias analysis techniques if there is an indication of systematic selection bias in either control group.
8.	**Information bias– case/control misclassification** eg cases incorrectly diagnosed as ARF, controls incorrectly diagnosed as non-cases	• Because ARF is a serious illness with long-term consequences, considerable effort goes into establishing the diagnosis when suspected cases are hospitalised. As noted in the Protocol, all cases will undergo expert clinical review to assign them to a case category. Only 'definite' and 'probable' cases will be retained in the analysis, and a sensitivity analysis will assess the effect of excluding 'probable' cases.<br>• Control misclassification as cases is unlikely as ARF is a rare disease, even in its sub-clinical forms. Controls will be specifically asked whether they have had ARF and will be excluded if they have.
9.	**Information bias– Poor recall** of some exposures by cases and controls (non-differential) especially early childhood exposures) or social desirability (eg smoking near children)	• Generally we will avoid asking about exposures that took places more than a few weeks previously.<br>• Generally focus on asking about 'usual' exposures as recall is likely to be more accurate than asking about exposures at a specific time<br>• Rely on linked health records for maternal and perinatal exposures
**10.**	**Information bias– Non reporting of some exposures**	• As above.

	that are considered undesirable (eg smoking near children)	• Use laboratory testing to help validate recall (eg hair nicotine testing) in subset of subjects.
11.	**Information bias – language difficulties and cultural differences** creating non-differential recall of exposures by cases and controls	• Using ethnically appropriate interviewers (i.e. matched ethnicity for Māori and Pacific participants)<br>• Selection and training of interviewers to improve their interview technique
12.	**Information bias - Differential recall** of exposures by cases and controls (eg differences in impact of case views on disease cause)	• Use identical questions and data collection methods for cases and controls<br>• Minimise number of questions that ask about exposures prior to illness (for cases), which is a different period to controls (but inevitable for a small set of specific exposures)<br>• Specifically ask subjects about their views on disease causality and use responses in sensitivity analysis (e.g. perform analyses that exclude cases who think a specific exposure is causal)
**13.**	**Information bias – Differential interview processes** for cases and NZHS controls, eg cases/parents will usually be interviewed in hospital, but controls will be interviewed in their homes	• For children, specify a consistent approach to identifying interview subject (proxy) so that the type of person interviewed is similar across both groups (for children this will usually be the main caregiver in all instances).<br>• Standard data collection methods and interviewer training about consistency of approach to cases and control regardless of setting.
14.	**Information bias – Differential effort by interviewer** to identify exposures for cases and controls	• Interviewers cannot be blinded to case/control status<br>• Use carefully constructed questions that focus on usual exposures

Supplementary Figure S1: Case recruitment algorithm used by clinicians

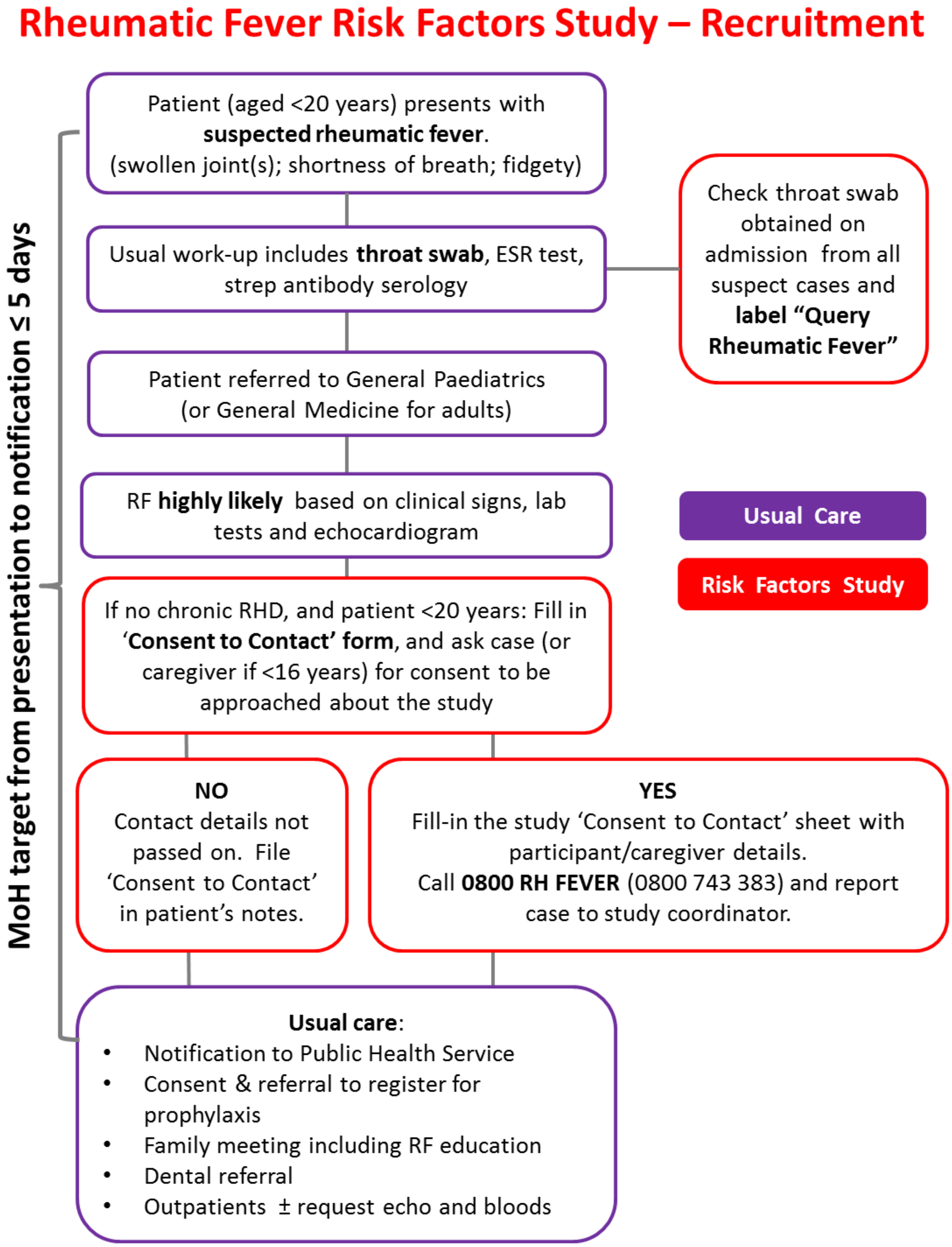

References

1. Entine, M., A survey of dental diseases as a diagnostic aid in rheumatic fever. *JAMA* **1949,** 38, (3), 303-8.
2. Grave, P. E., Social and environmental factors in the aetiology of rheumatic fever. *Med. J. Aust.* **1957,** 44, (18), 602-8.
3. Coburn, A. F., The concept of egg yolk as a dietary inhibitor to rheumatic susceptibility. *Lancet* **1960,** 1, (7129), 867-70.
4. Adanja, B.; Vlajinac, H.; Jarebinski, M., Socioeconomic factors in the etiology of rheumatic fever. *J. Hyg. Epidemiol. Microbiol. Immunol.* **1988,** 32, (3), 329-35.
5. Bahr, G. M.; Eales, L. J.; Nye, K. E.; Majeed, H. A.; Yousof, A. M.; Behbehani, K.; Rook, G. A., An association between Gc (vitamin D-binding protein) alleles and susceptibility to rheumatic fever. *Immunology* **1989,** 67, (1), 126-8.
6. Vlajinac, H.; Adanja, B.; Jarebinski, M., Socio-economic factors and rheumatic fever occurrence. Differences between patients with and without frequent sore throat. *J. Hyg. Epidemiol. Microbiol. Immunol.* **1989,** 33, (4), 471-6.
7. Vlajinac, H.; Adanja, B.; Marinkovic, J.; Jarebinski, M., Influence of socio-economic and other factors on rheumatic fever occurrence. *Eur. J. Epidemiol.* **1991,** 7, (6), 702-704.
8. Adanja, B. J.; Vlajinac, H. D.; Marinkovic, J. P.; Jarebinski, M. S., Rheumatic fever and diet. *Isr. J. Med. Sci.* **1991,** 27, (3), 161-3.
9. Thomas, D. R.; Salmon, R. L.; Kench, S. M.; Meadows, D.; Coleman, T. J.; Morgan-Capner, P.; Morgan, K. L., Zoonotic illness--determining risks and measuring effects: association between current animal exposure and a history of illness in a well characterised rural population in the UK. *J. Epidemiol. Community Health* **1994,** 48, (2), 151-5.
10. Zaman, M. M.; Yoshiike, N.; Chowdhury, A. H.; Jalil, M. Q.; Mahmud, R. S.; Faruque, G. M.; Rouf, M. A.; Haque, K. M.; Tanaka, H., Socio-economic deprivation associated with acute rheumatic fever. A hospital-based case-control study in Bangladesh. *Paediatr. Perinat. Epidemiol.* **1997,** 11, (3), 322-32.
11. Zaman, M. M.; Yoshiike, N.; Chowdhury, A. H.; Nakayama, T.; Yokoyama, T.; Faruque, G. M.; Rouf, M. A.; Haque, S.; Tanaka, H., Nutritional factors associated with rheumatic fever. *J. Trop. Pediatr.* **1998,** 44, (3), 142-7.
12. Zaman, M. M.; Yoshiike, N.; Rouf, M. A.; Haque, S.; Chowdhury, A. H.; Nakayama, T.; Tanaka, H., Association of rheumatic fever with serum albumin concentration and body iron stores in Bangladeshi children: case-control study. *BMJ* **1998,** 317, (7168), 1287-8.
13. Berdeli, A.; Celik, H. A.; Ozyurek, R.; Aydin, H. H., Involvement of immunoglobulin FcgammaRIIA and FcgammaRIIIB gene polymorphisms in susceptibility to rheumatic fever. *Clin. Biochem.* **2004,** 37, (10), 925-9.
14. Berdeli, A.; Celik, H. A.; Ozyurek, R.; Dogrusoz, B.; Aydin, H. H., TLR-2 gene Arg753Gln polymorphism is strongly associated with acute rheumatic fever in children. *J. Mol. Med. (Berl.)* **2005,** 83, (7), 535-41.
15. Berdeli, A.; Tabel, Y.; Celik, H. A.; Ozyurek, R.; Dogrusoz, B.; Aydin, H. H., Lack of association between TNFalpha gene polymorphism at position -308 and risk of acute rheumatic fever in Turkish patients. *Scand. J. Rheumatol.* **2006,** 35, (1), 44-7.
16. Kurahara, D. K.; Grandinetti, A.; Galario, J.; Reddy, D. V.; Tokuda, A.; Langan, S.; Tanabe, B.; Yamamoto, K. S.; Yamaga, K. M.; Kurahara, D. K.; Grandinetti, A.; Galario, J.; Reddy, D. V.; Tokuda, A.; Langan, S.; Tanabe, B.; Yamamoto, K. S.; Yamaga, K. M., Ethnic differences for developing rheumatic fever in a low-income group living in Hawaii. *Ethn. Dis.* **2006,** 16, (2), 357-61.
17. Hounie, A. G.; Pauls, D. L.; do Rosario-Campos, M. C.; Mercadante, M. T.; Diniz, J. B.; De Mathis, M. A.; De Mathis, M. E.; Chacon, P.; Shavitt, R. G.; Curi, M.; Guilherme, L.; Miguel, E.

C., Obsessive-compulsive spectrum disorders and rheumatic fever: a family study. *Biol. Psychiatry* **2007,** 61, (3), 266-72.

18. Seixas, A. A.; Hounie, A. G.; Fossaluza, V.; Curi, M.; Alvarenga, P. G.; De Mathis, M. A.; De Mathis, M. E.; Vallada, H.; Pauls, D.; de Braganca Pereira, C. A.; Miguel, E. C., Anxiety disorders and rheumatic Fever: is there an association? *CNS Spectr* **2008,** 13, (12), 1039-46.
19. Messias-Reason, I. J.; Schafranski, M. D.; Kremsner, P. G.; Kun, J. F., Ficolin 2 (FCN2) functional polymorphisms and the risk of rheumatic fever and rheumatic heart disease. *Clin. Exp. Immunol.* **2009,** 157, (3), 395-9.
20. Walker, K. G.; Cooper, M.; McCabe, K.; Hughes, J.; Mathiassen, W.; Lawrenson, J.; Wilmshurst, J. M., Markers of susceptibility to acute rheumatic fever: the B-cell antigen D8/17 is not robust as a marker in South Africa. *Cardiol. Young* **2011,** 21, (3), 328-33.
21. Col-Araz, N.; Pehlivan, S.; Baspinar, O.; Oguzkan-Balci, S.; Sever, T.; Balat, A., Role of cytokine gene (IFN-gamma, TNF-alpha, TGF-beta1, IL-6, and IL-10) polymorphisms in pathogenesis of acute rheumatic fever in Turkish children. *Eur. J. Pediatr.* **2012,** 171, (7), 1103-8.
22. Riaz, B. K.; Selim, S.; Karim, M. N.; Chowdhury, K. N.; Chowdhury, S. H.; Rahman, M. R., Risk factors of rheumatic heart disease in Bangladesh: a case-control study. *J. Health Popul. Nutr.* **2013,** 31, (1), 70-7.
23. Thornley, S.; Marshall, R. J.; Bach, K.; Koopu, P.; Reynolds, G.; Sundborn, G.; Ei, W. L., Sugar, dental caries and the incidence of acute rheumatic fever: a cohort study of Maori and Pacific children. *J. Epidemiol. Community Health* **2016**.
24. Thornley, S.; Marshall, R.; Jarrett, P.; Sundborn, G.; Reynolds, E.; Schofield, G., Scabies is strongly associated with acute rheumatic fever in a cohort study of Auckland children. *J. Paediatr. Child Health* **2018,** 54, (6), 625-632.
25. Caughey, D. E.; Douglas, R.; Wilson, W.; Hassall, I. B., HL-A antigens in Europeans and Maoris with rheumatic fever and rheumatic heart disease. *J. Rheumatol.* **1975,** 2, (3), 319-22.
26. McLaren, M. J.; Hawkins, D. M.; Koornhof, H. J.; Bloom, K. R.; Bramwell-Jones, D. M.; Cohen, E.; Gale, G. E.; Kanarek, K.; Lachman, A. S.; Lakier, J. B.; Pocock, W. A.; Barlow, J. B., Epidemiology of rheumatic heart disease in black shcoolchildren of Soweto, Johannesburg. *BMJ* **1975,** 3, (5981), 474-8.
27. Anabwani, G. M.; Amoa, A. B.; Muita, A. K., Epidemiology of rheumatic heart disease among primary school children in western Kenya. *Int. J. Cardiol.* **1989,** 23, (2), 249-52.
28. Coggon, D.; Barker, D. J.; Inskip, H.; Wield, G., Housing in early life and later mortality. *J. Epidemiol. Community Health* **1993,** 47, (5), 345-8.
29. Longo-Mbenza, B.; Bayekula, M.; Ngiyulu, R.; Kintoki, V. E.; Bikangi, N. F.; Seghers, K. V.; Lukoki, L. E.; Mandundu, M. F.; Manzanza, M.; Nlandu, Y., Survey of rheumatic heart disease in school children of Kinshasa town. *Int. J. Cardiol.* **1998,** 63, (3), 287-94.
30. Oli, K.; Porteous, J., Prevalence of rheumatic heart disease among school children in Addis Ababa. *East Afr. Med. J.* **1999,** 76, (11), 601-5.
31. Rizvi, S. F.; Khan, M. A.; Kundi, A.; Marsh, D. R.; Samad, A.; Pasha, O., Status of rheumatic heart disease in rural Pakistan. *Heart* **2004,** 90, (4), 394-9.
32. Chou, H. T.; Tsai, C. H.; Tsai, F. J., Association between angiotensin I-converting enzyme gene insertion/deletion polymorphism and risk of rheumatic heart disease. *Jpn. Heart J.* **2004,** 45, (6), 949-57.
33. Steer, A. C.; Kado, J.; Wilson, N.; Tuiketei, T.; Batzloff, M.; Waqatakirewa, L.; Mulholland, E. K.; Carapetis, J. R., High prevalence of rheumatic heart disease by clinical and echocardiographic screening among children in Fiji. *J. Heart Valve Dis.* **2009,** 18, (3), 327-336.
34. Azevedo, P. M.; Bauer, R.; Caparbo Vde, F.; Silva, C. A.; Bonfa, E.; Pereira, R. M., Interleukin-1 receptor antagonist gene (IL1RN) polymorphism possibly associated to severity of rheumatic carditis in a Brazilian cohort. *Cytokine* **2010,** 49, (1), 109-13.

35. Saxena, A.; Ramakrishnan, S.; Roy, A.; Seth, S.; Krishnan, A.; Misra, P.; Kalaivani, M.; Bhargava, B.; Flather, M. D.; Poole-Wilson, P. P., Prevalence and outcome of subclinical rheumatic heart disease in India: the RHEUMATIC (Rheumatic Heart Echo Utilisation and Monitoring Actuarial Trends in Indian Children) study. *Heart* **2011,** 97, (24), 2018-22.
36. Ba-Saddik, I. A.; Munibari, A. A.; Al-Naqeeb, M. S.; Parry, C. M.; Hart, C. A.; Cuevas, L. E.; Coulter, J. B. S., Prevalence of rheumatic heart disease among school-children in Aden, Yemen. *Ann Trop Paediatrics* **2011,** 31, (1), 37-46.
37. Dobson, J.; Steer, A. C.; Colquhoun, S.; Kado, J., Environmental factors and rheumatic heart disease in Fiji. *Pediatr. Cardiol.* **2012,** 33, (2), 332-6.
38. Okello, E.; Kakande, B.; Sebatta, E.; Kayima, J.; Kuteesa, M.; Mutatina, B.; Nyakoojo, W.; Lwabi, P.; Mondo, C. K.; Odoi-Adome, R.; Juergen, F., Socioeconomic and environmental risk factors among rheumatic heart disease patients in Uganda. *PLoS One* **2012,** 7, (8), e43917.
39. Rehman, S.; Akhtar, N.; Saba, N.; Munir, S.; Ahmed, W.; Mohyuddin, A.; Khanum, A., A study on the association of TNF-alpha(-308), IL-6(-174), IL-10(-1082) and IL-1Ra(VNTR) gene polymorphisms with rheumatic heart disease in Pakistani patients. *Cytokine* **2013,** 61, (2), 527-31.
40. Eriksson, J. G.; Kajantie, E.; Phillips, D. I.; Osmond, C.; Thornburg, K. L.; Barker, D. J., The developmental origins of chronic rheumatic heart disease. *Am. J. Hum. Biol.* **2013,** 25, (5), 655-8.
41. Mirabel, M.; Fauchier, T.; Bacquelin, R.; Tafflet, M.; Germain, A.; Robillard, C.; Rouchon, B.; Marijon, E.; Jouven, X., Echocardiography screening to detect rheumatic heart disease: A cohort study of schoolchildren in French Pacific Islands. *Int. J. Cardiol.* **2015,** 188, 89-95.
42. Azevedo, P. M.; Merriman, T. R.; Topless, R. K.; Wilson, N. J.; Crengle, S.; Lennon, D. R., Association study involving polymorphisms in IL-6, IL-1RA, and CTLA4 genes and rheumatic heart disease in New Zealand population of Maori and Pacific ancestry. *Cytokine* **2016,** 85, 201-6.
43. Parks, T.; Mirabel, M. M.; Kado, J.; Auckland, K.; Nowak, J.; Rautanen, A.; Mentzer, A. J.; Marijon, E.; Jouven, X.; Perman, M. L.; Cua, T.; Kauwe, J. K.; Allen, J. B.; Taylor, H.; Robson, K. J.; Deane, C. M.; Steer, A. C.; Hill, A. V. S.; Pacific Islands Rheumatic Heart Disease Genetics, N., Association between a common immunoglobulin heavy chain allele and rheumatic heart disease risk in Oceania. *Nat Commun* **2017,** 8, 14946.
44. Gray, L. A.; D'Antoine, H. A.; Tong, S. Y. C.; McKinnon, M.; Bessarab, D.; Brown, N.; Remenyi, B.; Steer, A.; Syn, G.; Blackwell, J. M.; Inouye, M.; Carapetis, J. R., Genome-wide analysis of genetic risk factors for rheumatic heart disease in Aboriginal Australians provides support for pathogenic molecular mimicry. *J. Infect. Dis.* **2017**.
